# Supplementary material for: Genome-wide identification, characterization and gene expression of BES1 transcription factor family in grapevine (Vitis vinifera L.)
Source: Sci Rep. 2023 Jan 5;13:240. doi: 10.1038/s41598-022-24407-y (PMC9816167; doi:10.1038/s41598-022-24407-y)
Supplement: Supplementary file 3 — Supplementary Information. [file 41598_2022_24407_MOESM3_ESM.zip › Vvi_Vvi/Vitis_vinifera.PN40024.v4.dna_sm.toplevel.fa.vs.Vitis_vinifera.PN40024.v4.dna_sm.toplevel.fa.html/14.html]

|  |  |  |  |  |  |  |  |  |  |  |  |  |  |
| --- | --- | --- | --- | --- | --- | --- | --- | --- | --- | --- | --- | --- | --- |
| Duplication depth | Reference chromosome | Collinear blocks | | | | | | | | | | | |
| 1 | Vitvi14g00026\_t001 |  | Vitvi05g01726\_t001 |  |  |  |  |  |  |  |  |  |  |
| 1 | Vitvi14g00028\_t001 |  | \| \| |  |  |  |  |  |  |  |  |  |  |
| 1 | Vitvi14g00029\_t001 |  | Vitvi05g00266\_t001 |  |  |  |  |  |  |  |  |  |  |
| 1 | Vitvi14g04000\_t001 |  | \| \| |  |  |  |  |  |  |  |  |  |  |
| 1 | Vitvi14g00030\_t002 |  | \| \| |  |  |  |  |  |  |  |  |  |  |
| 1 | Vitvi14g00031\_t001 |  | \| \| |  |  |  |  |  |  |  |  |  |  |
| 1 | Vitvi14g00033\_t001 |  | \| \| |  |  |  |  |  |  |  |  |  |  |
| 1 | Vitvi14g04001\_t001 |  | \| \| |  |  |  |  |  |  |  |  |  |  |
| 1 | Vitvi14g04002\_t001 |  | \| \| |  |  |  |  |  |  |  |  |  |  |
| 1 | Vitvi14g00034\_t001 |  | Vitvi05g01832\_t001 |  |  |  |  |  |  |  |  |  |  |
| 1 | Vitvi14g00035\_t001 |  | Vitvi05g00253\_t001 |  |  |  |  |  |  |  |  |  |  |
| 1 | Vitvi14g04003\_t001 |  | \| \| |  |  |  |  |  |  |  |  |  |  |
| 1 | Vitvi14g00036\_t001 |  | \| \| |  |  |  |  |  |  |  |  |  |  |
| 2 | Vitvi14g00037\_t001 |  | \| \| |  | Vitvi07g00495\_t001 |  |  |  |  |  |  |  |  |
| 2 | Vitvi14g00038\_t001 |  | Vitvi05g00250\_t001 |  | \| \| |  |  |  |  |  |  |  |  |
| 2 | Vitvi14g00039\_t001 |  | \| \| |  | Vitvi07g00494\_t001 |  |  |  |  |  |  |  |  |
| 2 | Vitvi14g00040\_t001 |  | \| \| |  | \| \| |  |  |  |  |  |  |  |  |
| 2 | Vitvi14g00041\_t001 |  | \| \| |  | \| \| |  |  |  |  |  |  |  |  |
| 2 | Vitvi14g00042\_t001 |  | Vitvi05g00247\_t001 |  | \| \| |  |  |  |  |  |  |  |  |
| 2 | Vitvi14g00043\_t001 |  | Vitvi05g00243\_t002 |  | Vitvi07g00488\_t001 |  |  |  |  |  |  |  |  |
| 2 | Vitvi14g02444\_t001 |  | Vitvi05g00242\_t001 |  | Vitvi07g02247\_t001 |  |  |  |  |  |  |  |  |
| 1 | Vitvi14g02445\_t001 |  |  |  | \| \| |  |  |  |  |  |  |  |  |
| 1 | Vitvi14g02446\_t001 |  |  |  | \| \| |  |  |  |  |  |  |  |  |
| 1 | Vitvi14g02447\_t001 |  |  |  | Vitvi07g00478\_t001 |  |  |  |  |  |  |  |  |
| 1 | Vitvi14g02448\_t001 |  |  |  | \| \| |  |  |  |  |  |  |  |  |
| 1 | Vitvi14g02451\_t001 |  |  |  | \| \| |  |  |  |  |  |  |  |  |
| 1 | Vitvi14g00047\_t002 |  |  |  | \| \| |  |  |  |  |  |  |  |  |
| 1 | Vitvi14g00049\_t001 |  |  |  | \| \| |  |  |  |  |  |  |  |  |
| 1 | Vitvi14g00050\_t002 |  |  |  | \| \| |  |  |  |  |  |  |  |  |
| 1 | Vitvi14g00051\_t001 |  |  |  | \| \| |  |  |  |  |  |  |  |  |
| 1 | Vitvi14g04004\_t001 |  |  |  | \| \| |  |  |  |  |  |  |  |  |
| 1 | Vitvi14g00052\_t003 |  |  |  | \| \| |  |  |  |  |  |  |  |  |
| 1 | Vitvi14g04005\_t001 |  |  |  | \| \| |  |  |  |  |  |  |  |  |
| 1 | Vitvi14g00053\_t003 |  |  |  | Vitvi07g00472\_t002 |  |  |  |  |  |  |  |  |
| 1 | Vitvi14g02452\_t001 |  |  |  | \| \| |  |  |  |  |  |  |  |  |
| 1 | Vitvi14g04006\_t001 |  |  |  | \| \| |  |  |  |  |  |  |  |  |
| 1 | Vitvi14g00055\_t001 |  |  |  | Vitvi07g00471\_t001 |  |  |  |  |  |  |  |  |
| 1 | Vitvi14g00056\_t001 |  |  |  | \| \| |  |  |  |  |  |  |  |  |
| 1 | Vitvi14g04007\_t001 |  |  |  | \| \| |  |  |  |  |  |  |  |  |
| 1 | Vitvi14g00057\_t002 |  |  |  | \| \| |  |  |  |  |  |  |  |  |
| 1 | Vitvi14g02453\_t001 |  |  |  | \| \| |  |  |  |  |  |  |  |  |
| 1 | Vitvi14g04008\_t001 |  |  |  | \| \| |  |  |  |  |  |  |  |  |
| 1 | Vitvi14g00058\_t001 |  |  |  | \| \| |  |  |  |  |  |  |  |  |
| 1 | Vitvi14g00059\_t001 |  |  |  | Vitvi07g04113\_t001 |  |  |  |  |  |  |  |  |
| 1 | Vitvi14g04009\_t001 |  |  |  | Vitvi07g00464\_t001 |  |  |  |  |  |  |  |  |
| 1 | Vitvi14g02455\_t001 |  |  |  | \| \| |  |  |  |  |  |  |  |  |
| 1 | Vitvi14g00061\_t001 |  |  |  | \| \| |  |  |  |  |  |  |  |  |
| 1 | Vitvi14g02456\_t001 |  |  |  | Vitvi07g00458\_t001 |  |  |  |  |  |  |  |  |
| 1 | Vitvi14g00062\_t001 |  |  |  | \| \| |  |  |  |  |  |  |  |  |
| 1 | Vitvi14g02457\_t001 |  |  |  | \| \| |  |  |  |  |  |  |  |  |
| 1 | Vitvi14g02458\_t001 |  |  |  | Vitvi07g00457\_t001 |  |  |  |  |  |  |  |  |
| 1 | Vitvi14g04010\_t001 |  |  |  | \| \| |  |  |  |  |  |  |  |  |
| 1 | Vitvi14g00063\_t001 |  |  |  | \| \| |  |  |  |  |  |  |  |  |
| 1 | Vitvi14g04011\_t001 |  |  |  | \| \| |  |  |  |  |  |  |  |  |
| 1 | Vitvi14g04012\_t001 |  |  |  | \| \| |  |  |  |  |  |  |  |  |
| 1 | Vitvi14g00064\_t001 |  |  |  | \| \| |  |  |  |  |  |  |  |  |
| 1 | Vitvi14g04013\_t001 |  |  |  | \| \| |  |  |  |  |  |  |  |  |
| 1 | Vitvi14g00065\_t001 |  |  |  | \| \| |  |  |  |  |  |  |  |  |
| 1 | Vitvi14g04014\_t001 |  |  |  | \| \| |  |  |  |  |  |  |  |  |
| 1 | Vitvi14g04015\_t001 |  |  |  | \| \| |  |  |  |  |  |  |  |  |
| 1 | Vitvi14g00066\_t001 |  |  |  | \| \| |  |  |  |  |  |  |  |  |
| 1 | Vitvi14g00067\_t001 |  |  |  | Vitvi07g00456\_t001 |  |  |  |  |  |  |  |  |
| 1 | Vitvi14g00068\_t001 |  |  |  | \| \| |  |  |  |  |  |  |  |  |
| 1 | Vitvi14g00069\_t001 |  |  |  | \| \| |  |  |  |  |  |  |  |  |
| 1 | Vitvi14g00070\_t001 |  |  |  | \| \| |  |  |  |  |  |  |  |  |
| 1 | Vitvi14g00071\_t001 |  |  |  | \| \| |  |  |  |  |  |  |  |  |
| 1 | Vitvi14g02462\_t001 |  |  |  | Vitvi07g02234\_t001 |  |  |  |  |  |  |  |  |
| 1 | Vitvi14g00072\_t001 |  |  |  | Vitvi07g00446\_t001 |  |  |  |  |  |  |  |  |
| 1 | Vitvi14g02463\_t001 |  |  |  | \| \| |  |  |  |  |  |  |  |  |
| 1 | Vitvi14g00073\_t001 |  |  |  | \| \| |  |  |  |  |  |  |  |  |
| 1 | Vitvi14g00074\_t001 |  |  |  | \| \| |  |  |  |  |  |  |  |  |
| 1 | Vitvi14g00075\_t001 |  |  |  | \| \| |  |  |  |  |  |  |  |  |
| 1 | Vitvi14g04016\_t001 |  |  |  | \| \| |  |  |  |  |  |  |  |  |
| 1 | Vitvi14g00076\_t001 |  |  |  | \| \| |  |  |  |  |  |  |  |  |
| 1 | Vitvi14g04017\_t001 |  |  |  | \| \| |  |  |  |  |  |  |  |  |
| 1 | Vitvi14g00078\_t001 |  |  |  | \| \| |  |  |  |  |  |  |  |  |
| 1 | Vitvi14g00079\_t001 |  |  |  | \| \| |  |  |  |  |  |  |  |  |
| 1 | Vitvi14g02465\_t001 |  |  |  | Vitvi07g00439\_t001 |  |  |  |  |  |  |  |  |
| 1 | Vitvi14g00080\_t001 |  |  |  | Vitvi07g00437\_t001 |  |  |  |  |  |  |  |  |
| 1 | Vitvi14g02466\_t001 |  |  |  | \| \| |  |  |  |  |  |  |  |  |
| 1 | Vitvi14g04018\_t001 |  |  |  | \| \| |  |  |  |  |  |  |  |  |
| 1 | Vitvi14g04019\_t001 |  |  |  | \| \| |  |  |  |  |  |  |  |  |
| 1 | Vitvi14g02469\_t001 |  |  |  | \| \| |  |  |  |  |  |  |  |  |
| 1 | Vitvi14g00082\_t001 |  |  |  | \| \| |  |  |  |  |  |  |  |  |
| 1 | Vitvi14g02470\_t001 |  |  |  | \| \| |  |  |  |  |  |  |  |  |
| 1 | Vitvi14g04020\_t001 |  |  |  | \| \| |  |  |  |  |  |  |  |  |
| 1 | Vitvi14g00083\_t001 |  |  |  | \| \| |  |  |  |  |  |  |  |  |
| 1 | Vitvi14g00084\_t001 |  |  |  | \| \| |  |  |  |  |  |  |  |  |
| 1 | Vitvi14g00086\_t001 |  |  |  | \| \| |  |  |  |  |  |  |  |  |
| 1 | Vitvi14g00087\_t001 |  |  |  | \| \| |  |  |  |  |  |  |  |  |
| 1 | Vitvi14g04021\_t001 |  |  |  | \| \| |  |  |  |  |  |  |  |  |
| 1 | Vitvi14g00088\_t001 |  |  |  | \| \| |  |  |  |  |  |  |  |  |
| 1 | Vitvi14g00089\_t001 |  |  |  | Vitvi07g00427\_t001 |  |  |  |  |  |  |  |  |
| 1 | Vitvi14g04022\_t001 |  |  |  | \| \| |  |  |  |  |  |  |  |  |
| 2 | Vitvi14g00090\_t001 |  | Vitvi05g00114\_t001 |  | Vitvi07g00423\_t001.3.6037826e |  |  |  |  |  |  |  |  |
| 2 | Vitvi14g00091\_t001 |  | \| \| |  | \| \| |  |  |  |  |  |  |  |  |
| 2 | Vitvi14g00092\_t002 |  | \| \| |  | \| \| |  |  |  |  |  |  |  |  |
| 2 | Vitvi14g02473\_t001 |  | \| \| |  | Vitvi07g02224\_t001 |  |  |  |  |  |  |  |  |
| 2 | Vitvi14g00093\_t001 |  | \| \| |  | \| \| |  |  |  |  |  |  |  |  |
| 2 | Vitvi14g02474\_t001 |  | \| \| |  | \| \| |  |  |  |  |  |  |  |  |
| 2 | Vitvi14g02475\_t001 |  | \| \| |  | \| \| |  |  |  |  |  |  |  |  |
| 2 | Vitvi14g00094\_t001 |  | Vitvi05g00108\_t001 |  | Vitvi07g00413\_t001 |  |  |  |  |  |  |  |  |
| 2 | Vitvi14g04023\_t001 |  | \| \| |  | \| \| |  |  |  |  |  |  |  |  |
| 2 | Vitvi14g04024\_t001 |  | \| \| |  | \| \| |  |  |  |  |  |  |  |  |
| 2 | Vitvi14g00095\_t001 |  | \| \| |  | \| \| |  |  |  |  |  |  |  |  |
| 2 | Vitvi14g00096\_t001 |  | \| \| |  | \| \| |  |  |  |  |  |  |  |  |
| 2 | Vitvi14g04025\_t001 |  | \| \| |  | \| \| |  |  |  |  |  |  |  |  |
| 2 | Vitvi14g00097\_t001 |  | \| \| |  | \| \| |  |  |  |  |  |  |  |  |
| 2 | Vitvi14g00098\_t001 |  | \| \| |  | \| \| |  |  |  |  |  |  |  |  |
| 2 | Vitvi14g00099\_t001 |  | \| \| |  | \| \| |  |  |  |  |  |  |  |  |
| 2 | Vitvi14g00100\_t001 |  | \| \| |  | \| \| |  |  |  |  |  |  |  |  |
| 2 | Vitvi14g00101\_t001 |  | \| \| |  | \| \| |  |  |  |  |  |  |  |  |
| 2 | Vitvi14g00102\_t001 |  | \| \| |  | Vitvi07g00408\_t001 |  |  |  |  |  |  |  |  |
| 2 | Vitvi14g02476\_t001 |  | \| \| |  | Vitvi07g00404\_t001 |  |  |  |  |  |  |  |  |
| 2 | Vitvi14g04026\_t002 |  | \| \| |  | \| \| |  |  |  |  |  |  |  |  |
| 2 | Vitvi14g00104\_t001 |  | Vitvi05g00100\_t001 |  | \| \| |  |  |  |  |  |  |  |  |
| 2 | Vitvi14g04027\_t001 |  | \| \| |  | \| \| |  |  |  |  |  |  |  |  |
| 2 | Vitvi14g04028\_t002 |  | Vitvi05g00097\_t001 |  | Vitvi07g00402\_t001 |  |  |  |  |  |  |  |  |
| 2 | Vitvi14g00108\_t001 |  | \| \| |  | \| \| |  |  |  |  |  |  |  |  |
| 2 | Vitvi14g02477\_t001 |  | Vitvi05g01767\_t001 |  | \| \| |  |  |  |  |  |  |  |  |
| 2 | Vitvi14g04029\_t001 |  | \| \| |  | \| \| |  |  |  |  |  |  |  |  |
| 2 | Vitvi14g00109\_t001 |  | \| \| |  | \| \| |  |  |  |  |  |  |  |  |
| 2 | Vitvi14g00110\_t001 |  | \| \| |  | \| \| |  |  |  |  |  |  |  |  |
| 2 | Vitvi14g00111\_t001 |  | \| \| |  | \| \| |  |  |  |  |  |  |  |  |
| 2 | Vitvi14g00112\_t001 |  | \| \| |  | \| \| |  |  |  |  |  |  |  |  |
| 2 | Vitvi14g02478\_t001 |  | \| \| |  | \| \| |  |  |  |  |  |  |  |  |
| 2 | Vitvi14g00113\_t001 |  | \| \| |  | Vitvi07g00390\_t001 |  |  |  |  |  |  |  |  |
| 2 | Vitvi14g00114\_t001 |  | Vitvi05g00083\_t001 |  | \| \| |  |  |  |  |  |  |  |  |
| 2 | Vitvi14g04030\_t001 |  | \| \| |  | \| \| |  |  |  |  |  |  |  |  |
| 2 | Vitvi14g00115\_t001 |  | \| \| |  | \| \| |  |  |  |  |  |  |  |  |
| 2 | Vitvi14g02479\_t001 |  | Vitvi05g01766\_t001 |  | Vitvi07g02216\_t001 |  |  |  |  |  |  |  |  |
| 2 | Vitvi14g00116\_t001 |  | \| \| |  | \| \| |  |  |  |  |  |  |  |  |
| 2 | Vitvi14g00117\_t001 |  | \| \| |  | \| \| |  |  |  |  |  |  |  |  |
| 2 | Vitvi14g00118\_t001 |  | \| \| |  | \| \| |  |  |  |  |  |  |  |  |
| 2 | Vitvi14g04031\_t001 |  | \| \| |  | \| \| |  |  |  |  |  |  |  |  |
| 2 | Vitvi14g04032\_t001 |  | \| \| |  | \| \| |  |  |  |  |  |  |  |  |
| 2 | Vitvi14g00119\_t001 |  | \| \| |  | \| \| |  |  |  |  |  |  |  |  |
| 2 | Vitvi14g00121\_t001 |  | \| \| |  | \| \| |  |  |  |  |  |  |  |  |
| 2 | Vitvi14g00122\_t001 |  | Vitvi05g01765\_t001 |  | Vitvi07g00382\_t001 |  |  |  |  |  |  |  |  |
| 2 | Vitvi14g04033\_t001 |  | \| \| |  | \| \| |  |  |  |  |  |  |  |  |
| 2 | Vitvi14g00123\_t001 |  | Vitvi05g00077\_t001 |  | Vitvi07g02214\_t001 |  |  |  |  |  |  |  |  |
| 2 | Vitvi14g00124\_t001 |  | Vitvi05g04016\_t001 |  | \| \| |  |  |  |  |  |  |  |  |
| 2 | Vitvi14g04034\_t001 |  | \| \| |  | \| \| |  |  |  |  |  |  |  |  |
| 2 | Vitvi14g00125\_t001 |  | \| \| |  | Vitvi07g00373\_t001 |  |  |  |  |  |  |  |  |
| 2 | Vitvi14g00126\_t001 |  | Vitvi05g01762\_t001 |  | Vitvi07g04089\_t001 |  |  |  |  |  |  |  |  |
| 1 | Vitvi14g02481\_t001 |  |  |  | \| \| |  |  |  |  |  |  |  |  |
| 1 | Vitvi14g04035\_t001 |  |  |  | \| \| |  |  |  |  |  |  |  |  |
| 1 | Vitvi14g00127\_t001 |  |  |  | Vitvi07g00368\_t001 |  |  |  |  |  |  |  |  |
| 1 | Vitvi14g00128\_t003 |  |  |  | \| \| |  |  |  |  |  |  |  |  |
| 1 | Vitvi14g00129\_t001 |  |  |  | \| \| |  |  |  |  |  |  |  |  |
| 1 | Vitvi14g00131\_t001 |  |  |  | \| \| |  |  |  |  |  |  |  |  |
| 1 | Vitvi14g00132\_t001 |  |  |  | \| \| |  |  |  |  |  |  |  |  |
| 1 | Vitvi14g02484\_t001 |  |  |  | \| \| |  |  |  |  |  |  |  |  |
| 1 | Vitvi14g04036\_t001 |  |  |  | \| \| |  |  |  |  |  |  |  |  |
| 1 | Vitvi14g04037\_t001 |  |  |  | \| \| |  |  |  |  |  |  |  |  |
| 1 | Vitvi14g04038\_t001 |  |  |  | \| \| |  |  |  |  |  |  |  |  |
| 1 | Vitvi14g04039\_t001 |  |  |  | \| \| |  |  |  |  |  |  |  |  |
| 1 | Vitvi14g00137\_t001 |  |  |  | \| \| |  |  |  |  |  |  |  |  |
| 1 | Vitvi14g00138\_t001 |  |  |  | Vitvi07g00362\_t001 |  |  |  |  |  |  |  |  |
| 1 | Vitvi14g00141\_t001 |  |  |  | \| \| |  |  |  |  |  |  |  |  |
| 1 | Vitvi14g00142\_t002 |  |  |  | \| \| |  |  |  |  |  |  |  |  |
| 1 | Vitvi14g00143\_t001 |  |  |  | \| \| |  |  |  |  |  |  |  |  |
| 1 | Vitvi14g00144\_t001 |  |  |  | \| \| |  |  |  |  |  |  |  |  |
| 1 | Vitvi14g02487\_t001 |  |  |  | \| \| |  |  |  |  |  |  |  |  |
| 1 | Vitvi14g04040\_t001 |  |  |  | \| \| |  |  |  |  |  |  |  |  |
| 1 | Vitvi14g04041\_t001 |  |  |  | \| \| |  |  |  |  |  |  |  |  |
| 1 | Vitvi14g00146\_t001 |  |  |  | \| \| |  |  |  |  |  |  |  |  |
| 1 | Vitvi14g02489\_t001 |  |  |  | \| \| |  |  |  |  |  |  |  |  |
| 1 | Vitvi14g02490\_t001 |  |  |  | \| \| |  |  |  |  |  |  |  |  |
| 1 | Vitvi14g02491\_t001 |  |  |  | \| \| |  |  |  |  |  |  |  |  |
| 1 | Vitvi14g04042\_t001 |  |  |  | \| \| |  |  |  |  |  |  |  |  |
| 1 | Vitvi14g04043\_t001 |  |  |  | \| \| |  |  |  |  |  |  |  |  |
| 1 | Vitvi14g02493\_t001 |  |  |  | \| \| |  |  |  |  |  |  |  |  |
| 1 | Vitvi14g02494\_t001 |  |  |  | \| \| |  |  |  |  |  |  |  |  |
| 1 | Vitvi14g02495\_t001 |  |  |  | \| \| |  |  |  |  |  |  |  |  |
| 1 | Vitvi14g04044\_t001 |  |  |  | \| \| |  |  |  |  |  |  |  |  |
| 1 | Vitvi14g02497\_t001 |  |  |  | \| \| |  |  |  |  |  |  |  |  |
| 1 | Vitvi14g04045\_t001 |  |  |  | \| \| |  |  |  |  |  |  |  |  |
| 1 | Vitvi14g00147\_t001 |  |  |  | \| \| |  |  |  |  |  |  |  |  |
| 1 | Vitvi14g04046\_t001 |  |  |  | \| \| |  |  |  |  |  |  |  |  |
| 1 | Vitvi14g00148\_t001 |  |  |  | \| \| |  |  |  |  |  |  |  |  |
| 1 | Vitvi14g04047\_t001 |  |  |  | \| \| |  |  |  |  |  |  |  |  |
| 1 | Vitvi14g00149\_t001 |  |  |  | \| \| |  |  |  |  |  |  |  |  |
| 1 | Vitvi14g00150\_t002 |  |  |  | \| \| |  |  |  |  |  |  |  |  |
| 1 | Vitvi14g00151\_t001 |  |  |  | Vitvi07g00346\_t001 |  |  |  |  |  |  |  |  |
| 1 | Vitvi14g00152\_t001 |  |  |  | \| \| |  |  |  |  |  |  |  |  |
| 1 | Vitvi14g00153\_t001 |  |  |  | \| \| |  |  |  |  |  |  |  |  |
| 1 | Vitvi14g02500\_t001 |  |  |  | \| \| |  |  |  |  |  |  |  |  |
| 1 | Vitvi14g02501\_t001 |  |  |  | \| \| |  |  |  |  |  |  |  |  |
| 1 | Vitvi14g04048\_t001 |  |  |  | \| \| |  |  |  |  |  |  |  |  |
| 1 | Vitvi14g00155\_t001 |  |  |  | \| \| |  |  |  |  |  |  |  |  |
| 1 | Vitvi14g00156\_t002 |  |  |  | \| \| |  |  |  |  |  |  |  |  |
| 1 | Vitvi14g00157\_t001 |  |  |  | \| \| |  |  |  |  |  |  |  |  |
| 1 | Vitvi14g02502\_t001 |  |  |  | \| \| |  |  |  |  |  |  |  |  |
| 1 | Vitvi14g00158\_t001 |  |  |  | \| \| |  |  |  |  |  |  |  |  |
| 1 | Vitvi14g00159\_t001 |  |  |  | Vitvi07g00330\_t001 |  |  |  |  |  |  |  |  |
| 1 | Vitvi14g00160\_t001 |  |  |  | \| \| |  |  |  |  |  |  |  |  |
| 1 | Vitvi14g02504\_t001 |  |  |  | \| \| |  |  |  |  |  |  |  |  |
| 1 | Vitvi14g00161\_t003 |  |  |  | \| \| |  |  |  |  |  |  |  |  |
| 1 | Vitvi14g00162\_t001 |  |  |  | \| \| |  |  |  |  |  |  |  |  |
| 1 | Vitvi14g00163\_t001 |  |  |  | \| \| |  |  |  |  |  |  |  |  |
| 1 | Vitvi14g00164\_t001 |  |  |  | \| \| |  |  |  |  |  |  |  |  |
| 1 | Vitvi14g00165\_t001 |  |  |  | \| \| |  |  |  |  |  |  |  |  |
| 1 | Vitvi14g00166\_t001 |  |  |  | \| \| |  |  |  |  |  |  |  |  |
| 1 | Vitvi14g00167\_t001 |  |  |  | \| \| |  |  |  |  |  |  |  |  |
| 1 | Vitvi14g00168\_t001 |  |  |  | \| \| |  |  |  |  |  |  |  |  |
| 1 | Vitvi14g02506\_t002 |  |  |  | \| \| |  |  |  |  |  |  |  |  |
| 1 | Vitvi14g00169\_t001 |  |  |  | \| \| |  |  |  |  |  |  |  |  |
| 1 | Vitvi14g00171\_t001 |  |  |  | \| \| |  |  |  |  |  |  |  |  |
| 1 | Vitvi14g04049\_t001 |  |  |  | \| \| |  |  |  |  |  |  |  |  |
| 1 | Vitvi14g00172\_t001 |  |  |  | \| \| |  |  |  |  |  |  |  |  |
| 1 | Vitvi14g00173\_t002 |  |  |  | \| \| |  |  |  |  |  |  |  |  |
| 1 | Vitvi14g00174\_t001 |  |  |  | \| \| |  |  |  |  |  |  |  |  |
| 1 | Vitvi14g04050\_t001 |  |  |  | \| \| |  |  |  |  |  |  |  |  |
| 1 | Vitvi14g00175\_t001 |  |  |  | \| \| |  |  |  |  |  |  |  |  |
| 1 | Vitvi14g00176\_t001 |  |  |  | \| \| |  |  |  |  |  |  |  |  |
| 1 | Vitvi14g00178\_t001 |  |  |  | \| \| |  |  |  |  |  |  |  |  |
| 1 | Vitvi14g00179\_t001 |  |  |  | \| \| |  |  |  |  |  |  |  |  |
| 1 | Vitvi14g00180\_t003 |  |  |  | Vitvi07g02202\_t001 |  |  |  |  |  |  |  |  |
| 1 | Vitvi14g02509\_t001 |  |  |  | Vitvi07g00316\_t001 |  |  |  |  |  |  |  |  |
| 0 | Vitvi14g04051\_t001 |  |  |  |  |  |  |  |  |  |  |  |  |
| 0 | Vitvi14g04052\_t001 |  |  |  |  |  |  |  |  |  |  |  |  |
| 0 | Vitvi14g02510\_t001 |  |  |  |  |  |  |  |  |  |  |  |  |
| 0 | Vitvi14g00182\_t001 |  |  |  |  |  |  |  |  |  |  |  |  |
| 0 | Vitvi14g00183\_t001 |  |  |  |  |  |  |  |  |  |  |  |  |
| 0 | Vitvi14g00184\_t001 |  |  |  |  |  |  |  |  |  |  |  |  |
| 0 | Vitvi14g00185\_t001 |  |  |  |  |  |  |  |  |  |  |  |  |
| 0 | Vitvi14g00186\_t001 |  |  |  |  |  |  |  |  |  |  |  |  |
| 0 | Vitvi14g04053\_t001 |  |  |  |  |  |  |  |  |  |  |  |  |
| 0 | Vitvi14g00189\_t001 |  |  |  |  |  |  |  |  |  |  |  |  |
| 0 | Vitvi14g00190\_t001 |  |  |  |  |  |  |  |  |  |  |  |  |
| 0 | Vitvi14g00191\_t001 |  |  |  |  |  |  |  |  |  |  |  |  |
| 0 | Vitvi14g00193\_t001 |  |  |  |  |  |  |  |  |  |  |  |  |
| 0 | Vitvi14g00194\_t001 |  |  |  |  |  |  |  |  |  |  |  |  |
| 0 | Vitvi14g00195\_t001 |  |  |  |  |  |  |  |  |  |  |  |  |
| 0 | Vitvi14g00196\_t001 |  |  |  |  |  |  |  |  |  |  |  |  |
| 0 | Vitvi14g02511\_t001 |  |  |  |  |  |  |  |  |  |  |  |  |
| 0 | Vitvi14g00197\_t001 |  |  |  |  |  |  |  |  |  |  |  |  |
| 0 | Vitvi14g00198\_t001 |  |  |  |  |  |  |  |  |  |  |  |  |
| 0 | Vitvi14g04054\_t001 |  |  |  |  |  |  |  |  |  |  |  |  |
| 0 | Vitvi14g02513\_t001 |  |  |  |  |  |  |  |  |  |  |  |  |
| 0 | Vitvi14g02514\_t001 |  |  |  |  |  |  |  |  |  |  |  |  |
| 0 | Vitvi14g04055\_t001 |  |  |  |  |  |  |  |  |  |  |  |  |
| 0 | Vitvi14g04056\_t001 |  |  |  |  |  |  |  |  |  |  |  |  |
| 0 | Vitvi14g04057\_t001 |  |  |  |  |  |  |  |  |  |  |  |  |
| 0 | Vitvi14g04058\_t001 |  |  |  |  |  |  |  |  |  |  |  |  |
| 0 | Vitvi14g04059\_t001 |  |  |  |  |  |  |  |  |  |  |  |  |
| 0 | Vitvi14g02516\_t001 |  |  |  |  |  |  |  |  |  |  |  |  |
| 0 | Vitvi14g04060\_t001 |  |  |  |  |  |  |  |  |  |  |  |  |
| 0 | Vitvi14g04061\_t001 |  |  |  |  |  |  |  |  |  |  |  |  |
| 0 | Vitvi14g04062\_t001 |  |  |  |  |  |  |  |  |  |  |  |  |
| 0 | Vitvi14g04063\_t001 |  |  |  |  |  |  |  |  |  |  |  |  |
| 0 | Vitvi14g04064\_t001 |  |  |  |  |  |  |  |  |  |  |  |  |
| 0 | Vitvi14g00206\_t001 |  |  |  |  |  |  |  |  |  |  |  |  |
| 0 | Vitvi14g04065\_t001 |  |  |  |  |  |  |  |  |  |  |  |  |
| 0 | Vitvi14g04066\_t001 |  |  |  |  |  |  |  |  |  |  |  |  |
| 0 | Vitvi14g00207\_t001 |  |  |  |  |  |  |  |  |  |  |  |  |
| 0 | Vitvi14g04067\_t001 |  |  |  |  |  |  |  |  |  |  |  |  |
| 0 | Vitvi14g04068\_t001 |  |  |  |  |  |  |  |  |  |  |  |  |
| 0 | Vitvi14g04069\_t001 |  |  |  |  |  |  |  |  |  |  |  |  |
| 0 | Vitvi14g04070\_t001 |  |  |  |  |  |  |  |  |  |  |  |  |
| 0 | Vitvi14g04071\_t001 |  |  |  |  |  |  |  |  |  |  |  |  |
| 0 | Vitvi14g04072\_t001 |  |  |  |  |  |  |  |  |  |  |  |  |
| 0 | Vitvi14g04073\_t001 |  |  |  |  |  |  |  |  |  |  |  |  |
| 0 | Vitvi14g04074\_t001 |  |  |  |  |  |  |  |  |  |  |  |  |
| 0 | Vitvi14g02521\_t001 |  |  |  |  |  |  |  |  |  |  |  |  |
| 0 | Vitvi14g02522\_t001 |  |  |  |  |  |  |  |  |  |  |  |  |
| 0 | Vitvi14g02523\_t001 |  |  |  |  |  |  |  |  |  |  |  |  |
| 0 | Vitvi14g02524\_t001 |  |  |  |  |  |  |  |  |  |  |  |  |
| 0 | Vitvi14g00215\_t002 |  |  |  |  |  |  |  |  |  |  |  |  |
| 0 | Vitvi14g00216\_t001 |  |  |  |  |  |  |  |  |  |  |  |  |
| 0 | Vitvi14g00217\_t001 |  |  |  |  |  |  |  |  |  |  |  |  |
| 0 | Vitvi14g02526\_t001 |  |  |  |  |  |  |  |  |  |  |  |  |
| 0 | Vitvi14g04075\_t001 |  |  |  |  |  |  |  |  |  |  |  |  |
| 0 | Vitvi14g04076\_t001 |  |  |  |  |  |  |  |  |  |  |  |  |
| 0 | Vitvi14g04077\_t001 |  |  |  |  |  |  |  |  |  |  |  |  |
| 0 | Vitvi14g04078\_t001 |  |  |  |  |  |  |  |  |  |  |  |  |
| 0 | Vitvi14g04079\_t001 |  |  |  |  |  |  |  |  |  |  |  |  |
| 0 | Vitvi14g04080\_t001 |  |  |  |  |  |  |  |  |  |  |  |  |
| 0 | Vitvi14g04081\_t001 |  |  |  |  |  |  |  |  |  |  |  |  |
| 0 | Vitvi14g00229\_t001 |  |  |  |  |  |  |  |  |  |  |  |  |
| 0 | Vitvi14g00230\_t001 |  |  |  |  |  |  |  |  |  |  |  |  |
| 0 | Vitvi14g00231\_t001 |  |  |  |  |  |  |  |  |  |  |  |  |
| 0 | Vitvi14g00232\_t001 |  |  |  |  |  |  |  |  |  |  |  |  |
| 0 | Vitvi14g00233\_t001 |  |  |  |  |  |  |  |  |  |  |  |  |
| 0 | Vitvi14g04082\_t001 |  |  |  |  |  |  |  |  |  |  |  |  |
| 1 | Vitvi14g00234\_t004 |  | Vitvi05g00472\_t001 |  |  |  |  |  |  |  |  |  |  |
| 1 | Vitvi14g04083\_t001 |  | \| \| |  |  |  |  |  |  |  |  |  |  |
| 1 | Vitvi14g00235\_t001 |  | \| \| |  |  |  |  |  |  |  |  |  |  |
| 1 | Vitvi14g00236\_t001 |  | \| \| |  |  |  |  |  |  |  |  |  |  |
| 1 | Vitvi14g00237\_t001 |  | \| \| |  |  |  |  |  |  |  |  |  |  |
| 1 | Vitvi14g00238\_t001 |  | \| \| |  |  |  |  |  |  |  |  |  |  |
| 1 | Vitvi14g04084\_t001 |  | Vitvi05g00463\_t001 |  |  |  |  |  |  |  |  |  |  |
| 1 | Vitvi14g02541\_t001 |  | \| \| |  |  |  |  |  |  |  |  |  |  |
| 1 | Vitvi14g00239\_t001 |  | \| \| |  |  |  |  |  |  |  |  |  |  |
| 1 | Vitvi14g00240\_t001 |  | \| \| |  |  |  |  |  |  |  |  |  |  |
| 1 | Vitvi14g04085\_t001 |  | \| \| |  |  |  |  |  |  |  |  |  |  |
| 1 | Vitvi14g04086\_t001 |  | \| \| |  |  |  |  |  |  |  |  |  |  |
| 1 | Vitvi14g04087\_t001 |  | \| \| |  |  |  |  |  |  |  |  |  |  |
| 1 | Vitvi14g04088\_t001 |  | \| \| |  |  |  |  |  |  |  |  |  |  |
| 1 | Vitvi14g02557\_t001 |  | \| \| |  |  |  |  |  |  |  |  |  |  |
| 1 | Vitvi14g00242\_t001 |  | \| \| |  |  |  |  |  |  |  |  |  |  |
| 1 | Vitvi14g00244\_t001 |  | \| \| |  |  |  |  |  |  |  |  |  |  |
| 1 | Vitvi14g00245\_t001 |  | \| \| |  |  |  |  |  |  |  |  |  |  |
| 1 | Vitvi14g00246\_t001 |  | \| \| |  |  |  |  |  |  |  |  |  |  |
| 1 | Vitvi14g02558\_t001 |  | \| \| |  |  |  |  |  |  |  |  |  |  |
| 1 | Vitvi14g02559\_t001 |  | \| \| |  |  |  |  |  |  |  |  |  |  |
| 1 | Vitvi14g00247\_t001 |  | \| \| |  |  |  |  |  |  |  |  |  |  |
| 1 | Vitvi14g00248\_t001 |  | Vitvi05g00444\_t001 |  |  |  |  |  |  |  |  |  |  |
| 1 | Vitvi14g00249\_t001 |  | \| \| |  |  |  |  |  |  |  |  |  |  |
| 1 | Vitvi14g00250\_t001 |  | \| \| |  |  |  |  |  |  |  |  |  |  |
| 1 | Vitvi14g00251\_t001 |  | \| \| |  |  |  |  |  |  |  |  |  |  |
| 1 | Vitvi14g00253\_t001 |  | Vitvi05g00443\_t001 |  |  |  |  |  |  |  |  |  |  |
| 1 | Vitvi14g00254\_t001 |  | \| \| |  |  |  |  |  |  |  |  |  |  |
| 1 | Vitvi14g00255\_t001 |  | \| \| |  |  |  |  |  |  |  |  |  |  |
| 1 | Vitvi14g00256\_t001 |  | \| \| |  |  |  |  |  |  |  |  |  |  |
| 1 | Vitvi14g02560\_t001 |  | Vitvi05g01879\_t001 |  |  |  |  |  |  |  |  |  |  |
| 1 | Vitvi14g00257\_t001 |  | \| \| |  |  |  |  |  |  |  |  |  |  |
| 1 | Vitvi14g00258\_t001 |  | \| \| |  |  |  |  |  |  |  |  |  |  |
| 1 | Vitvi14g00259\_t001 |  | \| \| |  |  |  |  |  |  |  |  |  |  |
| 1 | Vitvi14g00260\_t001 |  | \| \| |  |  |  |  |  |  |  |  |  |  |
| 1 | Vitvi14g04089\_t001 |  | \| \| |  |  |  |  |  |  |  |  |  |  |
| 1 | Vitvi14g00261\_t001 |  | \| \| |  |  |  |  |  |  |  |  |  |  |
| 1 | Vitvi14g00262\_t001 |  | \| \| |  |  |  |  |  |  |  |  |  |  |
| 1 | Vitvi14g02562\_t001 |  | \| \| |  |  |  |  |  |  |  |  |  |  |
| 1 | Vitvi14g00263\_t001 |  | \| \| |  |  |  |  |  |  |  |  |  |  |
| 1 | Vitvi14g00264\_t001 |  | Vitvi05g00435\_t001 |  |  |  |  |  |  |  |  |  |  |
| 1 | Vitvi14g04090\_t001 |  | \| \| |  |  |  |  |  |  |  |  |  |  |
| 1 | Vitvi14g00265\_t001 |  | \| \| |  |  |  |  |  |  |  |  |  |  |
| 1 | Vitvi14g00266\_t001 |  | \| \| |  |  |  |  |  |  |  |  |  |  |
| 1 | Vitvi14g00267\_t001 |  | Vitvi05g00433\_t001 |  |  |  |  |  |  |  |  |  |  |
| 1 | Vitvi14g00268\_t001 |  | \| \| |  |  |  |  |  |  |  |  |  |  |
| 1 | Vitvi14g00269\_t001 |  | Vitvi05g00431\_t001 |  |  |  |  |  |  |  |  |  |  |
| 1 | Vitvi14g04091\_t001 |  | \| \| |  |  |  |  |  |  |  |  |  |  |
| 1 | Vitvi14g00270\_t001 |  | Vitvi05g00426\_t001 |  |  |  |  |  |  |  |  |  |  |
| 1 | Vitvi14g00271\_t001 |  | Vitvi05g00425\_t002 |  |  |  |  |  |  |  |  |  |  |
| 0 | Vitvi14g00272\_t002 |  |  |  |  |  |  |  |  |  |  |  |  |
| 0 | Vitvi14g00273\_t001 |  |  |  |  |  |  |  |  |  |  |  |  |
| 0 | Vitvi14g00274\_t001 |  |  |  |  |  |  |  |  |  |  |  |  |
| 0 | Vitvi14g00275\_t001 |  |  |  |  |  |  |  |  |  |  |  |  |
| 0 | Vitvi14g00277\_t001 |  |  |  |  |  |  |  |  |  |  |  |  |
| 0 | Vitvi14g04092\_t001 |  |  |  |  |  |  |  |  |  |  |  |  |
| 0 | Vitvi14g00281\_t001 |  |  |  |  |  |  |  |  |  |  |  |  |
| 0 | Vitvi14g00278\_t001 |  |  |  |  |  |  |  |  |  |  |  |  |
| 0 | Vitvi14g00282\_t001 |  |  |  |  |  |  |  |  |  |  |  |  |
| 0 | Vitvi14g00283\_t001 |  |  |  |  |  |  |  |  |  |  |  |  |
| 0 | Vitvi14g02563\_t001 |  |  |  |  |  |  |  |  |  |  |  |  |
| 0 | Vitvi14g00284\_t001 |  |  |  |  |  |  |  |  |  |  |  |  |
| 0 | Vitvi14g02564\_t001 |  |  |  |  |  |  |  |  |  |  |  |  |
| 0 | Vitvi14g02565\_t001 |  |  |  |  |  |  |  |  |  |  |  |  |
| 0 | Vitvi14g00285\_t001 |  |  |  |  |  |  |  |  |  |  |  |  |
| 0 | Vitvi14g00286\_t001 |  |  |  |  |  |  |  |  |  |  |  |  |
| 0 | Vitvi14g00288\_t001 |  |  |  |  |  |  |  |  |  |  |  |  |
| 0 | Vitvi14g02566\_t001 |  |  |  |  |  |  |  |  |  |  |  |  |
| 0 | Vitvi14g04093\_t001 |  |  |  |  |  |  |  |  |  |  |  |  |
| 0 | Vitvi14g04094\_t001 |  |  |  |  |  |  |  |  |  |  |  |  |
| 0 | Vitvi14g00289\_t001 |  |  |  |  |  |  |  |  |  |  |  |  |
| 0 | Vitvi14g02568\_t001 |  |  |  |  |  |  |  |  |  |  |  |  |
| 0 | Vitvi14g00291\_t001 |  |  |  |  |  |  |  |  |  |  |  |  |
| 0 | Vitvi14g00292\_t001 |  |  |  |  |  |  |  |  |  |  |  |  |
| 0 | Vitvi14g04095\_t001 |  |  |  |  |  |  |  |  |  |  |  |  |
| 0 | Vitvi14g02569\_t001 |  |  |  |  |  |  |  |  |  |  |  |  |
| 0 | Vitvi14g04096\_t001 |  |  |  |  |  |  |  |  |  |  |  |  |
| 0 | Vitvi14g04097\_t001 |  |  |  |  |  |  |  |  |  |  |  |  |
| 0 | Vitvi14g04098\_t001 |  |  |  |  |  |  |  |  |  |  |  |  |
| 0 | Vitvi14g02571\_t001 |  |  |  |  |  |  |  |  |  |  |  |  |
| 0 | Vitvi14g04099\_t001 |  |  |  |  |  |  |  |  |  |  |  |  |
| 0 | Vitvi14g02572\_t001 |  |  |  |  |  |  |  |  |  |  |  |  |
| 0 | Vitvi14g00297\_t001 |  |  |  |  |  |  |  |  |  |  |  |  |
| 0 | Vitvi14g04100\_t001 |  |  |  |  |  |  |  |  |  |  |  |  |
| 0 | Vitvi14g02573\_t001 |  |  |  |  |  |  |  |  |  |  |  |  |
| 0 | Vitvi14g04101\_t001 |  |  |  |  |  |  |  |  |  |  |  |  |
| 0 | Vitvi14g04102\_t001 |  |  |  |  |  |  |  |  |  |  |  |  |
| 0 | Vitvi14g04103\_t001 |  |  |  |  |  |  |  |  |  |  |  |  |
| 0 | Vitvi14g00300\_t001 |  |  |  |  |  |  |  |  |  |  |  |  |
| 1 | Vitvi14g00301\_t001 |  | Vitvi07g00199\_t001 |  |  |  |  |  |  |  |  |  |  |
| 1 | Vitvi14g02576\_t001 |  | \| \| |  |  |  |  |  |  |  |  |  |  |
| 1 | Vitvi14g02577\_t001 |  | \| \| |  |  |  |  |  |  |  |  |  |  |
| 1 | Vitvi14g00303\_t001 |  | \| \| |  |  |  |  |  |  |  |  |  |  |
| 1 | Vitvi14g00304\_t001 |  | \| \| |  |  |  |  |  |  |  |  |  |  |
| 1 | Vitvi14g00305\_t001 |  | \| \| |  |  |  |  |  |  |  |  |  |  |
| 1 | Vitvi14g04105\_t001 |  | \| \| |  |  |  |  |  |  |  |  |  |  |
| 1 | Vitvi14g02578\_t001 |  | \| \| |  |  |  |  |  |  |  |  |  |  |
| 1 | Vitvi14g02579\_t001 |  | \| \| |  |  |  |  |  |  |  |  |  |  |
| 1 | Vitvi14g04106\_t001 |  | \| \| |  |  |  |  |  |  |  |  |  |  |
| 1 | Vitvi14g02580\_t001 |  | \| \| |  |  |  |  |  |  |  |  |  |  |
| 1 | Vitvi14g02581\_t001 |  | \| \| |  |  |  |  |  |  |  |  |  |  |
| 1 | Vitvi14g02582\_t001 |  | \| \| |  |  |  |  |  |  |  |  |  |  |
| 1 | Vitvi14g04107\_t001 |  | \| \| |  |  |  |  |  |  |  |  |  |  |
| 1 | Vitvi14g00311\_t001 |  | \| \| |  |  |  |  |  |  |  |  |  |  |
| 1 | Vitvi14g00312\_t001 |  | \| \| |  |  |  |  |  |  |  |  |  |  |
| 1 | Vitvi14g00314\_t001 |  | \| \| |  |  |  |  |  |  |  |  |  |  |
| 1 | Vitvi14g04108\_t001 |  | Vitvi07g00207\_t001 |  |  |  |  |  |  |  |  |  |  |
| 1 | Vitvi14g04109\_t001 |  | \| \| |  |  |  |  |  |  |  |  |  |  |
| 1 | Vitvi14g02586\_t001 |  | \| \| |  |  |  |  |  |  |  |  |  |  |
| 1 | Vitvi14g00315\_t001 |  | Vitvi07g00208\_t001 |  |  |  |  |  |  |  |  |  |  |
| 1 | Vitvi14g02587\_t001 |  | \| \| |  |  |  |  |  |  |  |  |  |  |
| 1 | Vitvi14g00316\_t001 |  | Vitvi07g00209\_t001 |  |  |  |  |  |  |  |  |  |  |
| 1 | Vitvi14g04110\_t001 |  | \| \| |  |  |  |  |  |  |  |  |  |  |
| 1 | Vitvi14g00317\_t001 |  | Vitvi07g00210\_t001 |  |  |  |  |  |  |  |  |  |  |
| 1 | Vitvi14g00318\_t001 |  | Vitvi07g00211\_t001 |  |  |  |  |  |  |  |  |  |  |
| 1 | Vitvi14g00319\_t001 |  | \| \| |  |  |  |  |  |  |  |  |  |  |
| 1 | Vitvi14g04111\_t001 |  | \| \| |  |  |  |  |  |  |  |  |  |  |
| 1 | Vitvi14g04112\_t001 |  | \| \| |  |  |  |  |  |  |  |  |  |  |
| 1 | Vitvi14g00321\_t001 |  | \| \| |  |  |  |  |  |  |  |  |  |  |
| 1 | Vitvi14g00322\_t001 |  | Vitvi07g00217\_t001 |  |  |  |  |  |  |  |  |  |  |
| 1 | Vitvi14g00323\_t001 |  | Vitvi07g00219\_t001 |  |  |  |  |  |  |  |  |  |  |
| 0 | Vitvi14g04113\_t001 |  |  |  |  |  |  |  |  |  |  |  |  |
| 0 | Vitvi14g04114\_t001 |  |  |  |  |  |  |  |  |  |  |  |  |
| 0 | Vitvi14g00325\_t003 |  |  |  |  |  |  |  |  |  |  |  |  |
| 0 | Vitvi14g02588\_t001 |  |  |  |  |  |  |  |  |  |  |  |  |
| 0 | Vitvi14g00326\_t001 |  |  |  |  |  |  |  |  |  |  |  |  |
| 0 | Vitvi14g02589\_t001 |  |  |  |  |  |  |  |  |  |  |  |  |
| 0 | Vitvi14g04115\_t001 |  |  |  |  |  |  |  |  |  |  |  |  |
| 0 | Vitvi14g04116\_t001 |  |  |  |  |  |  |  |  |  |  |  |  |
| 0 | Vitvi14g00327\_t001 |  |  |  |  |  |  |  |  |  |  |  |  |
| 0 | Vitvi14g02593\_t001 |  |  |  |  |  |  |  |  |  |  |  |  |
| 0 | Vitvi14g04117\_t001 |  |  |  |  |  |  |  |  |  |  |  |  |
| 0 | Vitvi14g04118\_t001 |  |  |  |  |  |  |  |  |  |  |  |  |
| 0 | Vitvi14g04119\_t001 |  |  |  |  |  |  |  |  |  |  |  |  |
| 0 | Vitvi14g04120\_t001 |  |  |  |  |  |  |  |  |  |  |  |  |
| 0 | Vitvi14g02597\_t001 |  |  |  |  |  |  |  |  |  |  |  |  |
| 0 | Vitvi14g04121\_t001 |  |  |  |  |  |  |  |  |  |  |  |  |
| 0 | Vitvi14g02599\_t001 |  |  |  |  |  |  |  |  |  |  |  |  |
| 0 | Vitvi14g04122\_t001 |  |  |  |  |  |  |  |  |  |  |  |  |
| 0 | Vitvi14g04123\_t001 |  |  |  |  |  |  |  |  |  |  |  |  |
| 0 | Vitvi14g04124\_t001 |  |  |  |  |  |  |  |  |  |  |  |  |
| 0 | Vitvi14g04125\_t001 |  |  |  |  |  |  |  |  |  |  |  |  |
| 0 | Vitvi14g04126\_t001 |  |  |  |  |  |  |  |  |  |  |  |  |
| 0 | Vitvi14g04127\_t001 |  |  |  |  |  |  |  |  |  |  |  |  |
| 0 | Vitvi14g02601\_t001 |  |  |  |  |  |  |  |  |  |  |  |  |
| 0 | Vitvi14g04128\_t001 |  |  |  |  |  |  |  |  |  |  |  |  |
| 0 | Vitvi14g02603\_t001 |  |  |  |  |  |  |  |  |  |  |  |  |
| 0 | Vitvi14g00334\_t001 |  |  |  |  |  |  |  |  |  |  |  |  |
| 0 | Vitvi14g04129\_t001 |  |  |  |  |  |  |  |  |  |  |  |  |
| 0 | Vitvi14g00335\_t001 |  |  |  |  |  |  |  |  |  |  |  |  |
| 0 | Vitvi14g00336\_t002 |  |  |  |  |  |  |  |  |  |  |  |  |
| 0 | Vitvi14g00337\_t001 |  |  |  |  |  |  |  |  |  |  |  |  |
| 0 | Vitvi14g00338\_t001 |  |  |  |  |  |  |  |  |  |  |  |  |
| 0 | Vitvi14g00339\_t001 |  |  |  |  |  |  |  |  |  |  |  |  |
| 0 | Vitvi14g00340\_t001 |  |  |  |  |  |  |  |  |  |  |  |  |
| 0 | Vitvi14g00343\_t001 |  |  |  |  |  |  |  |  |  |  |  |  |
| 0 | Vitvi14g00345\_t001 |  |  |  |  |  |  |  |  |  |  |  |  |
| 0 | Vitvi14g00346\_t001 |  |  |  |  |  |  |  |  |  |  |  |  |
| 0 | Vitvi14g02605\_t001 |  |  |  |  |  |  |  |  |  |  |  |  |
| 0 | Vitvi14g00347\_t001 |  |  |  |  |  |  |  |  |  |  |  |  |
| 0 | Vitvi14g00348\_t001 |  |  |  |  |  |  |  |  |  |  |  |  |
| 0 | Vitvi14g00349\_t001 |  |  |  |  |  |  |  |  |  |  |  |  |
| 0 | Vitvi14g04130\_t001 |  |  |  |  |  |  |  |  |  |  |  |  |
| 0 | Vitvi14g00352\_t001 |  |  |  |  |  |  |  |  |  |  |  |  |
| 0 | Vitvi14g04131\_t001 |  |  |  |  |  |  |  |  |  |  |  |  |
| 0 | Vitvi14g02607\_t005 |  |  |  |  |  |  |  |  |  |  |  |  |
| 1 | Vitvi14g02608\_t001 |  | Vitvi14g04296\_t001 |  |  |  |  |  |  |  |  |  |  |
| 1 | Vitvi14g04132\_t001 |  | \| \| |  |  |  |  |  |  |  |  |  |  |
| 1 | Vitvi14g04133\_t001 |  | \| \| |  |  |  |  |  |  |  |  |  |  |
| 1 | Vitvi14g04134\_t001 |  | \| \| |  |  |  |  |  |  |  |  |  |  |
| 1 | Vitvi14g04135\_t001 |  | \| \| |  |  |  |  |  |  |  |  |  |  |
| 1 | Vitvi14g04136\_t001 |  | \| \| |  |  |  |  |  |  |  |  |  |  |
| 1 | Vitvi14g04137\_t001 |  | \| \| |  |  |  |  |  |  |  |  |  |  |
| 1 | Vitvi14g00359\_t001 |  | \| \| |  |  |  |  |  |  |  |  |  |  |
| 1 | Vitvi14g02614\_t001 |  | \| \| |  |  |  |  |  |  |  |  |  |  |
| 1 | Vitvi14g02615\_t001 |  | \| \| |  |  |  |  |  |  |  |  |  |  |
| 1 | Vitvi14g04138\_t001 |  | \| \| |  |  |  |  |  |  |  |  |  |  |
| 1 | Vitvi14g02617\_t001 |  | Vitvi14g04299\_t001 |  |  |  |  |  |  |  |  |  |  |
| 1 | Vitvi14g04139\_t001 |  | \| \| |  |  |  |  |  |  |  |  |  |  |
| 1 | Vitvi14g00363\_t001 |  | Vitvi14g04300\_t001 |  |  |  |  |  |  |  |  |  |  |
| 1 | Vitvi14g04140\_t001 |  | \| \| |  |  |  |  |  |  |  |  |  |  |
| 1 | Vitvi14g04141\_t001 |  | \| \| |  |  |  |  |  |  |  |  |  |  |
| 1 | Vitvi14g04142\_t001 |  | \| \| |  |  |  |  |  |  |  |  |  |  |
| 1 | Vitvi14g04143\_t001 |  | \| \| |  |  |  |  |  |  |  |  |  |  |
| 2 | Vitvi14g00366\_t001 |  | \| \| |  | Vitvi14g04308\_t001 |  |  |  |  |  |  |  |  |
| 2 | Vitvi14g00367\_t001 |  | \| \| |  | \| \| |  |  |  |  |  |  |  |  |
| 2 | Vitvi14g04144\_t001 |  | \| \| |  | \| \| |  |  |  |  |  |  |  |  |
| 2 | Vitvi14g04145\_t001 |  | \| \| |  | \| \| |  |  |  |  |  |  |  |  |
| 2 | Vitvi14g04146\_t001 |  | \| \| |  | \| \| |  |  |  |  |  |  |  |  |
| 2 | Vitvi14g02626\_t001 |  | \| \| |  | \| \| |  |  |  |  |  |  |  |  |
| 2 | Vitvi14g00369\_t001 |  | \| \| |  | \| \| |  |  |  |  |  |  |  |  |
| 2 | Vitvi14g04147\_t001 |  | \| \| |  | \| \| |  |  |  |  |  |  |  |  |
| 2 | Vitvi14g04148\_t001 |  | \| \| |  | Vitvi14g02762\_t001 |  |  |  |  |  |  |  |  |
| 2 | Vitvi14g04149\_t001 |  | \| \| |  | \| \| |  |  |  |  |  |  |  |  |
| 2 | Vitvi14g00370\_t001 |  | \| \| |  | \| \| |  |  |  |  |  |  |  |  |
| 2 | Vitvi14g00372\_t001 |  | \| \| |  | \| \| |  |  |  |  |  |  |  |  |
| 2 | Vitvi14g04150\_t001 |  | \| \| |  | \| \| |  |  |  |  |  |  |  |  |
| 2 | Vitvi14g04151\_t001 |  | Vitvi14g00729\_t001 |  | Vitvi14g00729\_t001 |  |  |  |  |  |  |  |  |
| 2 | Vitvi14g00374\_t001 |  | \| \| |  | Vitvi14g02758\_t001 |  |  |  |  |  |  |  |  |
| 2 | Vitvi14g04152\_t001 |  | \| \| |  | \| \| |  |  |  |  |  |  |  |  |
| 2 | Vitvi14g04153\_t001 |  | Vitvi14g02761\_t001 |  | Vitvi14g04301\_t001 |  |  |  |  |  |  |  |  |
| 2 | Vitvi14g02633\_t001 |  | \| \| |  | Vitvi14g04300\_t001 |  |  |  |  |  |  |  |  |
| 1 | Vitvi14g00377\_t001 |  | Vitvi14g04308\_t001 |  |  |  |  |  |  |  |  |  |  |
| 0 | Vitvi14g04154\_t001 |  |  |  |  |  |  |  |  |  |  |  |  |
| 0 | Vitvi14g04155\_t001 |  |  |  |  |  |  |  |  |  |  |  |  |
| 0 | Vitvi14g04156\_t001 |  |  |  |  |  |  |  |  |  |  |  |  |
| 0 | Vitvi14g00381\_t001 |  |  |  |  |  |  |  |  |  |  |  |  |
| 0 | Vitvi14g04157\_t001 |  |  |  |  |  |  |  |  |  |  |  |  |
| 0 | Vitvi14g00382\_t002 |  |  |  |  |  |  |  |  |  |  |  |  |
| 0 | Vitvi14g00383\_t002 |  |  |  |  |  |  |  |  |  |  |  |  |
| 0 | Vitvi14g00385\_t001 |  |  |  |  |  |  |  |  |  |  |  |  |
| 0 | Vitvi14g04158\_t001 |  |  |  |  |  |  |  |  |  |  |  |  |
| 0 | Vitvi14g00386\_t002 |  |  |  |  |  |  |  |  |  |  |  |  |
| 0 | Vitvi14g04159\_t001 |  |  |  |  |  |  |  |  |  |  |  |  |
| 0 | Vitvi14g04160\_t001 |  |  |  |  |  |  |  |  |  |  |  |  |
| 0 | Vitvi14g00387\_t001 |  |  |  |  |  |  |  |  |  |  |  |  |
| 0 | Vitvi14g02637\_t001 |  |  |  |  |  |  |  |  |  |  |  |  |
| 0 | Vitvi14g00388\_t001 |  |  |  |  |  |  |  |  |  |  |  |  |
| 0 | Vitvi14g00389\_t002 |  |  |  |  |  |  |  |  |  |  |  |  |
| 0 | Vitvi14g00391\_t001 |  |  |  |  |  |  |  |  |  |  |  |  |
| 0 | Vitvi14g00392\_t001 |  |  |  |  |  |  |  |  |  |  |  |  |
| 0 | Vitvi14g00393\_t001 |  |  |  |  |  |  |  |  |  |  |  |  |
| 0 | Vitvi14g04161\_t001 |  |  |  |  |  |  |  |  |  |  |  |  |
| 0 | Vitvi14g00395\_t001 |  |  |  |  |  |  |  |  |  |  |  |  |
| 0 | Vitvi14g00396\_t001 |  |  |  |  |  |  |  |  |  |  |  |  |
| 0 | Vitvi14g04162\_t001 |  |  |  |  |  |  |  |  |  |  |  |  |
| 0 | Vitvi14g04163\_t001 |  |  |  |  |  |  |  |  |  |  |  |  |
| 0 | Vitvi14g02640\_t001 |  |  |  |  |  |  |  |  |  |  |  |  |
| 0 | Vitvi14g04164\_t001 |  |  |  |  |  |  |  |  |  |  |  |  |
| 0 | Vitvi14g02642\_t001 |  |  |  |  |  |  |  |  |  |  |  |  |
| 0 | Vitvi14g02643\_t001 |  |  |  |  |  |  |  |  |  |  |  |  |
| 0 | Vitvi14g00397\_t001 |  |  |  |  |  |  |  |  |  |  |  |  |
| 0 | Vitvi14g00398\_t001 |  |  |  |  |  |  |  |  |  |  |  |  |
| 0 | Vitvi14g00399\_t001 |  |  |  |  |  |  |  |  |  |  |  |  |
| 0 | Vitvi14g00400\_t001 |  |  |  |  |  |  |  |  |  |  |  |  |
| 0 | Vitvi14g04165\_t001 |  |  |  |  |  |  |  |  |  |  |  |  |
| 0 | Vitvi14g04166\_t001 |  |  |  |  |  |  |  |  |  |  |  |  |
| 0 | Vitvi14g02645\_t001 |  |  |  |  |  |  |  |  |  |  |  |  |
| 0 | Vitvi14g04167\_t001 |  |  |  |  |  |  |  |  |  |  |  |  |
| 0 | Vitvi14g04168\_t001 |  |  |  |  |  |  |  |  |  |  |  |  |
| 0 | Vitvi14g04169\_t001 |  |  |  |  |  |  |  |  |  |  |  |  |
| 0 | Vitvi14g02646\_t001 |  |  |  |  |  |  |  |  |  |  |  |  |
| 0 | Vitvi14g02647\_t001 |  |  |  |  |  |  |  |  |  |  |  |  |
| 0 | Vitvi14g00402\_t001 |  |  |  |  |  |  |  |  |  |  |  |  |
| 0 | Vitvi14g04170\_t001 |  |  |  |  |  |  |  |  |  |  |  |  |
| 0 | Vitvi14g00404\_t004 |  |  |  |  |  |  |  |  |  |  |  |  |
| 0 | Vitvi14g00405\_t001 |  |  |  |  |  |  |  |  |  |  |  |  |
| 0 | Vitvi14g00406\_t001 |  |  |  |  |  |  |  |  |  |  |  |  |
| 0 | Vitvi14g00407\_t001 |  |  |  |  |  |  |  |  |  |  |  |  |
| 0 | Vitvi14g00409\_t001 |  |  |  |  |  |  |  |  |  |  |  |  |
| 0 | Vitvi14g00410\_t001 |  |  |  |  |  |  |  |  |  |  |  |  |
| 0 | Vitvi14g04171\_t001 |  |  |  |  |  |  |  |  |  |  |  |  |
| 0 | Vitvi14g00413\_t001 |  |  |  |  |  |  |  |  |  |  |  |  |
| 0 | Vitvi14g00415\_t001 |  |  |  |  |  |  |  |  |  |  |  |  |
| 0 | Vitvi14g04172\_t001 |  |  |  |  |  |  |  |  |  |  |  |  |
| 0 | Vitvi14g04173\_t001 |  |  |  |  |  |  |  |  |  |  |  |  |
| 0 | Vitvi14g00416\_t001 |  |  |  |  |  |  |  |  |  |  |  |  |
| 0 | Vitvi14g00417\_t001 |  |  |  |  |  |  |  |  |  |  |  |  |
| 0 | Vitvi14g00418\_t001 |  |  |  |  |  |  |  |  |  |  |  |  |
| 0 | Vitvi14g00419\_t001 |  |  |  |  |  |  |  |  |  |  |  |  |
| 0 | Vitvi14g00420\_t001 |  |  |  |  |  |  |  |  |  |  |  |  |
| 0 | Vitvi14g00421\_t001 |  |  |  |  |  |  |  |  |  |  |  |  |
| 0 | Vitvi14g00422\_t001 |  |  |  |  |  |  |  |  |  |  |  |  |
| 0 | Vitvi14g02653\_t003 |  |  |  |  |  |  |  |  |  |  |  |  |
| 0 | Vitvi14g00423\_t001 |  |  |  |  |  |  |  |  |  |  |  |  |
| 0 | Vitvi14g00424\_t001 |  |  |  |  |  |  |  |  |  |  |  |  |
| 0 | Vitvi14g02655\_t001 |  |  |  |  |  |  |  |  |  |  |  |  |
| 0 | Vitvi14g00425\_t001 |  |  |  |  |  |  |  |  |  |  |  |  |
| 0 | Vitvi14g04174\_t001 |  |  |  |  |  |  |  |  |  |  |  |  |
| 0 | Vitvi14g00427\_t001 |  |  |  |  |  |  |  |  |  |  |  |  |
| 1 | Vitvi14g00428\_t001 |  | Vitvi05g00541\_t001 |  |  |  |  |  |  |  |  |  |  |
| 1 | Vitvi14g00429\_t001 |  | Vitvi05g00543\_t001 |  |  |  |  |  |  |  |  |  |  |
| 1 | Vitvi14g00430\_t001 |  | \| \| |  |  |  |  |  |  |  |  |  |  |
| 1 | Vitvi14g00431\_t001 |  | \| \| |  |  |  |  |  |  |  |  |  |  |
| 1 | Vitvi14g00432\_t002 |  | \| \| |  |  |  |  |  |  |  |  |  |  |
| 1 | Vitvi14g00433\_t001 |  | \| \| |  |  |  |  |  |  |  |  |  |  |
| 1 | Vitvi14g04175\_t001 |  | \| \| |  |  |  |  |  |  |  |  |  |  |
| 1 | Vitvi14g00434\_t001 |  | \| \| |  |  |  |  |  |  |  |  |  |  |
| 1 | Vitvi14g00437\_t001 |  | Vitvi05g00549\_t001 |  |  |  |  |  |  |  |  |  |  |
| 1 | Vitvi14g00438\_t001 |  | Vitvi05g00553\_t001 |  |  |  |  |  |  |  |  |  |  |
| 1 | Vitvi14g04176\_t001 |  | \| \| |  |  |  |  |  |  |  |  |  |  |
| 1 | Vitvi14g00439\_t001 |  | \| \| |  |  |  |  |  |  |  |  |  |  |
| 1 | Vitvi14g00440\_t002 |  | \| \| |  |  |  |  |  |  |  |  |  |  |
| 1 | Vitvi14g00441\_t001 |  | \| \| |  |  |  |  |  |  |  |  |  |  |
| 1 | Vitvi14g02657\_t001 |  | \| \| |  |  |  |  |  |  |  |  |  |  |
| 1 | Vitvi14g00445\_t001 |  | \| \| |  |  |  |  |  |  |  |  |  |  |
| 1 | Vitvi14g00446\_t001 |  | \| \| |  |  |  |  |  |  |  |  |  |  |
| 1 | Vitvi14g04177\_t001 |  | \| \| |  |  |  |  |  |  |  |  |  |  |
| 1 | Vitvi14g04178\_t001 |  | \| \| |  |  |  |  |  |  |  |  |  |  |
| 1 | Vitvi14g04179\_t001 |  | \| \| |  |  |  |  |  |  |  |  |  |  |
| 1 | Vitvi14g04180\_t001 |  | \| \| |  |  |  |  |  |  |  |  |  |  |
| 1 | Vitvi14g02659\_t001 |  | Vitvi05g00565\_t001 |  |  |  |  |  |  |  |  |  |  |
| 1 | Vitvi14g04181\_t001 |  | \| \| |  |  |  |  |  |  |  |  |  |  |
| 1 | Vitvi14g04182\_t001 |  | \| \| |  |  |  |  |  |  |  |  |  |  |
| 1 | Vitvi14g04183\_t001 |  | \| \| |  |  |  |  |  |  |  |  |  |  |
| 1 | Vitvi14g04184\_t001 |  | \| \| |  |  |  |  |  |  |  |  |  |  |
| 1 | Vitvi14g04185\_t001 |  | \| \| |  |  |  |  |  |  |  |  |  |  |
| 1 | Vitvi14g02663\_t001 |  | \| \| |  |  |  |  |  |  |  |  |  |  |
| 1 | Vitvi14g04186\_t001 |  | \| \| |  |  |  |  |  |  |  |  |  |  |
| 1 | Vitvi14g00456\_t001 |  | \| \| |  |  |  |  |  |  |  |  |  |  |
| 1 | Vitvi14g02660\_t001 |  | \| \| |  |  |  |  |  |  |  |  |  |  |
| 2 | Vitvi14g00458\_t001 |  | Vitvi05g00585\_t001 |  | Vitvi07g00071\_t001 |  |  |  |  |  |  |  |  |
| 2 | Vitvi14g00459\_t001 |  | \| \| |  | \| \| |  |  |  |  |  |  |  |  |
| 2 | Vitvi14g04187\_t001 |  | \| \| |  | \| \| |  |  |  |  |  |  |  |  |
| 2 | Vitvi14g00461\_t001 |  | Vitvi05g00588\_t001 |  | \| \| |  |  |  |  |  |  |  |  |
| 2 | Vitvi14g04188\_t001 |  | \| \| |  | \| \| |  |  |  |  |  |  |  |  |
| 2 | Vitvi14g00465\_t001 |  | \| \| |  | \| \| |  |  |  |  |  |  |  |  |
| 2 | Vitvi14g00466\_t001 |  | \| \| |  | \| \| |  |  |  |  |  |  |  |  |
| 2 | Vitvi14g00468\_t001 |  | Vitvi05g00605\_t001 |  | \| \| |  |  |  |  |  |  |  |  |
| 2 | Vitvi14g00469\_t001 |  | \| \| |  | Vitvi07g00065\_t001 |  |  |  |  |  |  |  |  |
| 2 | Vitvi14g00470\_t001 |  | \| \| |  | \| \| |  |  |  |  |  |  |  |  |
| 2 | Vitvi14g00471\_t002 |  | Vitvi05g00614\_t003 |  | \| \| |  |  |  |  |  |  |  |  |
| 2 | Vitvi14g00472\_t001 |  | \| \| |  | \| \| |  |  |  |  |  |  |  |  |
| 2 | Vitvi14g04189\_t001 |  | \| \| |  | \| \| |  |  |  |  |  |  |  |  |
| 2 | Vitvi14g00473\_t001 |  | \| \| |  | Vitvi07g00049\_t001 |  |  |  |  |  |  |  |  |
| 2 | Vitvi14g00475\_t001 |  | \| \| |  | \| \| |  |  |  |  |  |  |  |  |
| 2 | Vitvi14g00476\_t001 |  | \| \| |  | \| \| |  |  |  |  |  |  |  |  |
| 2 | Vitvi14g00477\_t001 |  | Vitvi05g00624\_t001 |  | Vitvi07g00046\_t001 |  |  |  |  |  |  |  |  |
| 2 | Vitvi14g00478\_t001 |  | \| \| |  | \| \| |  |  |  |  |  |  |  |  |
| 2 | Vitvi14g00479\_t001 |  | \| \| |  | \| \| |  |  |  |  |  |  |  |  |
| 2 | Vitvi14g04190\_t001 |  | \| \| |  | \| \| |  |  |  |  |  |  |  |  |
| 2 | Vitvi14g04191\_t001 |  | \| \| |  | \| \| |  |  |  |  |  |  |  |  |
| 2 | Vitvi14g00480\_t001 |  | \| \| |  | \| \| |  |  |  |  |  |  |  |  |
| 2 | Vitvi14g02666\_t001 |  | \| \| |  | \| \| |  |  |  |  |  |  |  |  |
| 2 | Vitvi14g00481\_t001 |  | \| \| |  | \| \| |  |  |  |  |  |  |  |  |
| 2 | Vitvi14g00482\_t001 |  | \| \| |  | \| \| |  |  |  |  |  |  |  |  |
| 2 | Vitvi14g00483\_t001 |  | \| \| |  | Vitvi07g00042\_t001 |  |  |  |  |  |  |  |  |
| 2 | Vitvi14g00484\_t001 |  | Vitvi05g00630\_t001 |  | \| \| |  |  |  |  |  |  |  |  |
| 2 | Vitvi14g00487\_t001 |  | \| \| |  | \| \| |  |  |  |  |  |  |  |  |
| 2 | Vitvi14g00488\_t001 |  | \| \| |  | \| \| |  |  |  |  |  |  |  |  |
| 2 | Vitvi14g00489\_t001 |  | \| \| |  | Vitvi07g00035\_t001 |  |  |  |  |  |  |  |  |
| 2 | Vitvi14g00490\_t001 |  | Vitvi05g00634\_t001 |  | Vitvi07g00031\_t001 |  |  |  |  |  |  |  |  |
| 2 | Vitvi14g00491\_t001 |  | \| \| |  | \| \| |  |  |  |  |  |  |  |  |
| 2 | Vitvi14g02667\_t001 |  | Vitvi05g01906\_t001 |  | Vitvi07g00030\_t001 |  |  |  |  |  |  |  |  |
| 2 | Vitvi14g04192\_t001 |  | \| \| |  | \| \| |  |  |  |  |  |  |  |  |
| 2 | Vitvi14g00493\_t001 |  | \| \| |  | \| \| |  |  |  |  |  |  |  |  |
| 2 | Vitvi14g00495\_t001 |  | Vitvi05g00656\_t001 |  | \| \| |  |  |  |  |  |  |  |  |
| 2 | Vitvi14g04193\_t001 |  | \| \| |  | \| \| |  |  |  |  |  |  |  |  |
| 2 | Vitvi14g04194\_t001 |  | \| \| |  | \| \| |  |  |  |  |  |  |  |  |
| 2 | Vitvi14g04195\_t001 |  | \| \| |  | \| \| |  |  |  |  |  |  |  |  |
| 2 | Vitvi14g02670\_t001 |  | \| \| |  | \| \| |  |  |  |  |  |  |  |  |
| 2 | Vitvi14g00497\_t001 |  | \| \| |  | \| \| |  |  |  |  |  |  |  |  |
| 2 | Vitvi14g00499\_t001 |  | \| \| |  | \| \| |  |  |  |  |  |  |  |  |
| 2 | Vitvi14g04196\_t001 |  | \| \| |  | \| \| |  |  |  |  |  |  |  |  |
| 2 | Vitvi14g00500\_t001 |  | \| \| |  | \| \| |  |  |  |  |  |  |  |  |
| 2 | Vitvi14g02674\_t001 |  | \| \| |  | \| \| |  |  |  |  |  |  |  |  |
| 2 | Vitvi14g02675\_t001 |  | Vitvi05g04163\_t001 |  | \| \| |  |  |  |  |  |  |  |  |
| 1 | Vitvi14g02676\_t001 |  |  |  | \| \| |  |  |  |  |  |  |  |  |
| 1 | Vitvi14g04197\_t001 |  |  |  | \| \| |  |  |  |  |  |  |  |  |
| 1 | Vitvi14g04198\_t001 |  |  |  | \| \| |  |  |  |  |  |  |  |  |
| 1 | Vitvi14g04199\_t001 |  |  |  | \| \| |  |  |  |  |  |  |  |  |
| 1 | Vitvi14g04200\_t001 |  |  |  | \| \| |  |  |  |  |  |  |  |  |
| 1 | Vitvi14g04201\_t001 |  |  |  | \| \| |  |  |  |  |  |  |  |  |
| 1 | Vitvi14g04202\_t001 |  |  |  | \| \| |  |  |  |  |  |  |  |  |
| 1 | Vitvi14g00503\_t001 |  |  |  | \| \| |  |  |  |  |  |  |  |  |
| 1 | Vitvi14g04203\_t001 |  |  |  | \| \| |  |  |  |  |  |  |  |  |
| 1 | Vitvi14g04204\_t001 |  |  |  | \| \| |  |  |  |  |  |  |  |  |
| 1 | Vitvi14g02679\_t001 |  |  |  | \| \| |  |  |  |  |  |  |  |  |
| 1 | Vitvi14g02680\_t001 |  |  |  | Vitvi07g00020\_t001 |  |  |  |  |  |  |  |  |
| 1 | Vitvi14g00507\_t001 |  |  |  | \| \| |  |  |  |  |  |  |  |  |
| 2 | Vitvi14g00508\_t001 |  | Vitvi07g00013\_t001 |  | \| \| |  |  |  |  |  |  |  |  |
| 2 | Vitvi14g04205\_t001 |  | \| \| |  | \| \| |  |  |  |  |  |  |  |  |
| 2 | Vitvi14g00509\_t001 |  | Vitvi07g00017\_t001 |  | Vitvi07g00017\_t001 |  |  |  |  |  |  |  |  |
| 1 | Vitvi14g00510\_t001 |  | \| \| |  |  |  |  |  |  |  |  |  |  |
| 1 | Vitvi14g00511\_t001 |  | \| \| |  |  |  |  |  |  |  |  |  |  |
| 1 | Vitvi14g04206\_t001 |  | \| \| |  |  |  |  |  |  |  |  |  |  |
| 1 | Vitvi14g04207\_t001 |  | \| \| |  |  |  |  |  |  |  |  |  |  |
| 1 | Vitvi14g04208\_t001 |  | \| \| |  |  |  |  |  |  |  |  |  |  |
| 1 | Vitvi14g04209\_t001 |  | \| \| |  |  |  |  |  |  |  |  |  |  |
| 1 | Vitvi14g00518\_t001 |  | \| \| |  |  |  |  |  |  |  |  |  |  |
| 1 | Vitvi14g04210\_t001 |  | \| \| |  |  |  |  |  |  |  |  |  |  |
| 1 | Vitvi14g04211\_t001 |  | \| \| |  |  |  |  |  |  |  |  |  |  |
| 1 | Vitvi14g04212\_t001 |  | \| \| |  |  |  |  |  |  |  |  |  |  |
| 1 | Vitvi14g00519\_t001 |  | \| \| |  |  |  |  |  |  |  |  |  |  |
| 1 | Vitvi14g02690\_t001 |  | \| \| |  |  |  |  |  |  |  |  |  |  |
| 1 | Vitvi14g00521\_t001 |  | Vitvi07g00018\_t001 |  |  |  |  |  |  |  |  |  |  |
| 1 | Vitvi14g00528\_t002 |  | \| \| |  |  |  |  |  |  |  |  |  |  |
| 1 | Vitvi14g02691\_t001 |  | \| \| |  |  |  |  |  |  |  |  |  |  |
| 1 | Vitvi14g00535\_t001 |  | \| \| |  |  |  |  |  |  |  |  |  |  |
| 1 | Vitvi14g00536\_t001 |  | \| \| |  |  |  |  |  |  |  |  |  |  |
| 1 | Vitvi14g00537\_t001 |  | Vitvi07g00024\_t001 |  |  |  |  |  |  |  |  |  |  |
| 1 | Vitvi14g04213\_t001 |  | \| \| |  |  |  |  |  |  |  |  |  |  |
| 1 | Vitvi14g04214\_t001 |  | \| \| |  |  |  |  |  |  |  |  |  |  |
| 1 | Vitvi14g02049\_t001 |  | Vitvi07g02066\_t001 |  |  |  |  |  |  |  |  |  |  |
| 1 | Vitvi14g00539\_t001 |  | \| \| |  |  |  |  |  |  |  |  |  |  |
| 1 | Vitvi14g02693\_t001 |  | \| \| |  |  |  |  |  |  |  |  |  |  |
| 1 | Vitvi14g02695\_t001 |  | \| \| |  |  |  |  |  |  |  |  |  |  |
| 1 | Vitvi14g00540\_t001 |  | Vitvi07g00026\_t001 |  |  |  |  |  |  |  |  |  |  |
| 0 | Vitvi14g00541\_t001 |  |  |  |  |  |  |  |  |  |  |  |  |
| 0 | Vitvi14g02696\_t001 |  |  |  |  |  |  |  |  |  |  |  |  |
| 0 | Vitvi14g04215\_t001 |  |  |  |  |  |  |  |  |  |  |  |  |
| 0 | Vitvi14g00544\_t001 |  |  |  |  |  |  |  |  |  |  |  |  |
| 0 | Vitvi14g00545\_t001 |  |  |  |  |  |  |  |  |  |  |  |  |
| 0 | Vitvi14g00546\_t001 |  |  |  |  |  |  |  |  |  |  |  |  |
| 0 | Vitvi14g00547\_t001 |  |  |  |  |  |  |  |  |  |  |  |  |
| 0 | Vitvi14g02697\_t001 |  |  |  |  |  |  |  |  |  |  |  |  |
| 0 | Vitvi14g04216\_t001 |  |  |  |  |  |  |  |  |  |  |  |  |
| 0 | Vitvi14g04217\_t001 |  |  |  |  |  |  |  |  |  |  |  |  |
| 0 | Vitvi14g00551\_t001 |  |  |  |  |  |  |  |  |  |  |  |  |
| 0 | Vitvi14g00555\_t001 |  |  |  |  |  |  |  |  |  |  |  |  |
| 0 | Vitvi14g00556\_t001 |  |  |  |  |  |  |  |  |  |  |  |  |
| 0 | Vitvi14g00557\_t001 |  |  |  |  |  |  |  |  |  |  |  |  |
| 0 | Vitvi14g00558\_t001 |  |  |  |  |  |  |  |  |  |  |  |  |
| 0 | Vitvi14g00560\_t001 |  |  |  |  |  |  |  |  |  |  |  |  |
| 0 | Vitvi14g00561\_t001 |  |  |  |  |  |  |  |  |  |  |  |  |
| 0 | Vitvi14g00562\_t001 |  |  |  |  |  |  |  |  |  |  |  |  |
| 0 | Vitvi14g00564\_t001 |  |  |  |  |  |  |  |  |  |  |  |  |
| 0 | Vitvi14g02699\_t001 |  |  |  |  |  |  |  |  |  |  |  |  |
| 0 | Vitvi14g04218\_t001 |  |  |  |  |  |  |  |  |  |  |  |  |
| 0 | Vitvi14g02700\_t001 |  |  |  |  |  |  |  |  |  |  |  |  |
| 0 | Vitvi14g04219\_t001 |  |  |  |  |  |  |  |  |  |  |  |  |
| 0 | Vitvi14g04220\_t001 |  |  |  |  |  |  |  |  |  |  |  |  |
| 0 | Vitvi14g04221\_t001 |  |  |  |  |  |  |  |  |  |  |  |  |
| 0 | Vitvi14g04222\_t001 |  |  |  |  |  |  |  |  |  |  |  |  |
| 0 | Vitvi14g04223\_t001 |  |  |  |  |  |  |  |  |  |  |  |  |
| 0 | Vitvi14g02703\_t001 |  |  |  |  |  |  |  |  |  |  |  |  |
| 0 | Vitvi14g04224\_t001 |  |  |  |  |  |  |  |  |  |  |  |  |
| 0 | Vitvi14g04225\_t001 |  |  |  |  |  |  |  |  |  |  |  |  |
| 0 | Vitvi14g00569\_t001 |  |  |  |  |  |  |  |  |  |  |  |  |
| 0 | Vitvi14g04226\_t001 |  |  |  |  |  |  |  |  |  |  |  |  |
| 0 | Vitvi14g04227\_t001 |  |  |  |  |  |  |  |  |  |  |  |  |
| 0 | Vitvi14g02706\_t001 |  |  |  |  |  |  |  |  |  |  |  |  |
| 0 | Vitvi14g04228\_t001 |  |  |  |  |  |  |  |  |  |  |  |  |
| 0 | Vitvi14g04229\_t001 |  |  |  |  |  |  |  |  |  |  |  |  |
| 0 | Vitvi14g04230\_t001 |  |  |  |  |  |  |  |  |  |  |  |  |
| 0 | Vitvi14g04231\_t001 |  |  |  |  |  |  |  |  |  |  |  |  |
| 0 | Vitvi14g02707\_t001 |  |  |  |  |  |  |  |  |  |  |  |  |
| 0 | Vitvi14g04232\_t001 |  |  |  |  |  |  |  |  |  |  |  |  |
| 0 | Vitvi14g04233\_t001 |  |  |  |  |  |  |  |  |  |  |  |  |
| 0 | Vitvi14g04234\_t001 |  |  |  |  |  |  |  |  |  |  |  |  |
| 0 | Vitvi14g00574\_t001 |  |  |  |  |  |  |  |  |  |  |  |  |
| 0 | Vitvi14g02710\_t001 |  |  |  |  |  |  |  |  |  |  |  |  |
| 0 | Vitvi14g02711\_t001 |  |  |  |  |  |  |  |  |  |  |  |  |
| 0 | Vitvi14g02712\_t001 |  |  |  |  |  |  |  |  |  |  |  |  |
| 0 | Vitvi14g02714\_t001 |  |  |  |  |  |  |  |  |  |  |  |  |
| 0 | Vitvi14g04235\_t001 |  |  |  |  |  |  |  |  |  |  |  |  |
| 0 | Vitvi14g04236\_t001 |  |  |  |  |  |  |  |  |  |  |  |  |
| 0 | Vitvi14g02719\_t001 |  |  |  |  |  |  |  |  |  |  |  |  |
| 0 | Vitvi14g00576\_t001 |  |  |  |  |  |  |  |  |  |  |  |  |
| 0 | Vitvi14g04237\_t001 |  |  |  |  |  |  |  |  |  |  |  |  |
| 0 | Vitvi14g04238\_t001 |  |  |  |  |  |  |  |  |  |  |  |  |
| 0 | Vitvi14g02720\_t001 |  |  |  |  |  |  |  |  |  |  |  |  |
| 0 | Vitvi14g02721\_t001 |  |  |  |  |  |  |  |  |  |  |  |  |
| 0 | Vitvi14g02722\_t001 |  |  |  |  |  |  |  |  |  |  |  |  |
| 0 | Vitvi14g04239\_t001 |  |  |  |  |  |  |  |  |  |  |  |  |
| 0 | Vitvi14g04240\_t001 |  |  |  |  |  |  |  |  |  |  |  |  |
| 0 | Vitvi14g04241\_t001 |  |  |  |  |  |  |  |  |  |  |  |  |
| 0 | Vitvi14g04242\_t001 |  |  |  |  |  |  |  |  |  |  |  |  |
| 0 | Vitvi14g00579\_t001 |  |  |  |  |  |  |  |  |  |  |  |  |
| 0 | Vitvi14g00580\_t001 |  |  |  |  |  |  |  |  |  |  |  |  |
| 0 | Vitvi14g04243\_t001 |  |  |  |  |  |  |  |  |  |  |  |  |
| 0 | Vitvi14g00581\_t001 |  |  |  |  |  |  |  |  |  |  |  |  |
| 0 | Vitvi14g04244\_t001 |  |  |  |  |  |  |  |  |  |  |  |  |
| 0 | Vitvi14g00584\_t001 |  |  |  |  |  |  |  |  |  |  |  |  |
| 0 | Vitvi14g04245\_t001 |  |  |  |  |  |  |  |  |  |  |  |  |
| 0 | Vitvi14g04246\_t001 |  |  |  |  |  |  |  |  |  |  |  |  |
| 0 | Vitvi14g04247\_t001 |  |  |  |  |  |  |  |  |  |  |  |  |
| 0 | Vitvi14g04248\_t001 |  |  |  |  |  |  |  |  |  |  |  |  |
| 0 | Vitvi14g04249\_t001 |  |  |  |  |  |  |  |  |  |  |  |  |
| 0 | Vitvi14g04250\_t001 |  |  |  |  |  |  |  |  |  |  |  |  |
| 0 | Vitvi14g00589\_t001 |  |  |  |  |  |  |  |  |  |  |  |  |
| 0 | Vitvi14g04251\_t001 |  |  |  |  |  |  |  |  |  |  |  |  |
| 0 | Vitvi14g04252\_t001 |  |  |  |  |  |  |  |  |  |  |  |  |
| 0 | Vitvi14g04253\_t001 |  |  |  |  |  |  |  |  |  |  |  |  |
| 0 | Vitvi14g00592\_t001 |  |  |  |  |  |  |  |  |  |  |  |  |
| 0 | Vitvi14g00593\_t001 |  |  |  |  |  |  |  |  |  |  |  |  |
| 0 | Vitvi14g04254\_t001 |  |  |  |  |  |  |  |  |  |  |  |  |
| 0 | Vitvi14g04255\_t001 |  |  |  |  |  |  |  |  |  |  |  |  |
| 0 | Vitvi14g02729\_t001 |  |  |  |  |  |  |  |  |  |  |  |  |
| 0 | Vitvi14g00598\_t001 |  |  |  |  |  |  |  |  |  |  |  |  |
| 0 | Vitvi14g04256\_t001 |  |  |  |  |  |  |  |  |  |  |  |  |
| 0 | Vitvi14g00599\_t001 |  |  |  |  |  |  |  |  |  |  |  |  |
| 0 | Vitvi14g00600\_t003 |  |  |  |  |  |  |  |  |  |  |  |  |
| 0 | Vitvi14g04257\_t001 |  |  |  |  |  |  |  |  |  |  |  |  |
| 0 | Vitvi14g04258\_t001 |  |  |  |  |  |  |  |  |  |  |  |  |
| 0 | Vitvi14g00605\_t001 |  |  |  |  |  |  |  |  |  |  |  |  |
| 0 | Vitvi14g04259\_t001 |  |  |  |  |  |  |  |  |  |  |  |  |
| 0 | Vitvi14g00608\_t001 |  |  |  |  |  |  |  |  |  |  |  |  |
| 0 | Vitvi14g04260\_t001 |  |  |  |  |  |  |  |  |  |  |  |  |
| 0 | Vitvi14g04261\_t001 |  |  |  |  |  |  |  |  |  |  |  |  |
| 0 | Vitvi14g04262\_t001 |  |  |  |  |  |  |  |  |  |  |  |  |
| 0 | Vitvi14g00614\_t001 |  |  |  |  |  |  |  |  |  |  |  |  |
| 0 | Vitvi14g00615\_t001 |  |  |  |  |  |  |  |  |  |  |  |  |
| 0 | Vitvi14g04263\_t001 |  |  |  |  |  |  |  |  |  |  |  |  |
| 0 | Vitvi14g00618\_t002 |  |  |  |  |  |  |  |  |  |  |  |  |
| 0 | Vitvi14g00619\_t003 |  |  |  |  |  |  |  |  |  |  |  |  |
| 0 | Vitvi14g00620\_t001 |  |  |  |  |  |  |  |  |  |  |  |  |
| 0 | Vitvi14g04264\_t001 |  |  |  |  |  |  |  |  |  |  |  |  |
| 0 | Vitvi14g00623\_t001 |  |  |  |  |  |  |  |  |  |  |  |  |
| 0 | Vitvi14g00626\_t001 |  |  |  |  |  |  |  |  |  |  |  |  |
| 0 | Vitvi14g04265\_t001 |  |  |  |  |  |  |  |  |  |  |  |  |
| 0 | Vitvi14g00627\_t001 |  |  |  |  |  |  |  |  |  |  |  |  |
| 0 | Vitvi14g00628\_t001 |  |  |  |  |  |  |  |  |  |  |  |  |
| 0 | Vitvi14g02732\_t001 |  |  |  |  |  |  |  |  |  |  |  |  |
| 0 | Vitvi14g02733\_t001 |  |  |  |  |  |  |  |  |  |  |  |  |
| 0 | Vitvi14g04266\_t001 |  |  |  |  |  |  |  |  |  |  |  |  |
| 0 | Vitvi14g00629\_t001 |  |  |  |  |  |  |  |  |  |  |  |  |
| 0 | Vitvi14g04267\_t001 |  |  |  |  |  |  |  |  |  |  |  |  |
| 0 | Vitvi14g04268\_t001 |  |  |  |  |  |  |  |  |  |  |  |  |
| 0 | Vitvi14g04269\_t001 |  |  |  |  |  |  |  |  |  |  |  |  |
| 0 | Vitvi14g00632\_t001 |  |  |  |  |  |  |  |  |  |  |  |  |
| 0 | Vitvi14g04270\_t001 |  |  |  |  |  |  |  |  |  |  |  |  |
| 0 | Vitvi14g00637\_t001 |  |  |  |  |  |  |  |  |  |  |  |  |
| 0 | Vitvi14g00638\_t001 |  |  |  |  |  |  |  |  |  |  |  |  |
| 0 | Vitvi14g00640\_t001 |  |  |  |  |  |  |  |  |  |  |  |  |
| 0 | Vitvi14g00643\_t001 |  |  |  |  |  |  |  |  |  |  |  |  |
| 0 | Vitvi14g04271\_t001 |  |  |  |  |  |  |  |  |  |  |  |  |
| 0 | Vitvi14g00647\_t001 |  |  |  |  |  |  |  |  |  |  |  |  |
| 0 | Vitvi14g00648\_t001 |  |  |  |  |  |  |  |  |  |  |  |  |
| 0 | Vitvi14g02737\_t001 |  |  |  |  |  |  |  |  |  |  |  |  |
| 0 | Vitvi14g04272\_t001 |  |  |  |  |  |  |  |  |  |  |  |  |
| 0 | Vitvi14g04273\_t001 |  |  |  |  |  |  |  |  |  |  |  |  |
| 0 | Vitvi14g04274\_t001 |  |  |  |  |  |  |  |  |  |  |  |  |
| 0 | Vitvi14g04275\_t001 |  |  |  |  |  |  |  |  |  |  |  |  |
| 0 | Vitvi14g04276\_t001 |  |  |  |  |  |  |  |  |  |  |  |  |
| 0 | Vitvi14g00651\_t001 |  |  |  |  |  |  |  |  |  |  |  |  |
| 0 | Vitvi14g02739\_t001 |  |  |  |  |  |  |  |  |  |  |  |  |
| 0 | Vitvi14g00654\_t001 |  |  |  |  |  |  |  |  |  |  |  |  |
| 0 | Vitvi14g00656\_t001 |  |  |  |  |  |  |  |  |  |  |  |  |
| 0 | Vitvi14g04277\_t001 |  |  |  |  |  |  |  |  |  |  |  |  |
| 0 | Vitvi14g00658\_t001 |  |  |  |  |  |  |  |  |  |  |  |  |
| 0 | Vitvi14g00663\_t001 |  |  |  |  |  |  |  |  |  |  |  |  |
| 0 | Vitvi14g04278\_t001 |  |  |  |  |  |  |  |  |  |  |  |  |
| 0 | Vitvi14g04279\_t001 |  |  |  |  |  |  |  |  |  |  |  |  |
| 0 | Vitvi14g04280\_t001 |  |  |  |  |  |  |  |  |  |  |  |  |
| 0 | Vitvi14g00666\_t001 |  |  |  |  |  |  |  |  |  |  |  |  |
| 0 | Vitvi14g00667\_t001 |  |  |  |  |  |  |  |  |  |  |  |  |
| 0 | Vitvi14g00668\_t001 |  |  |  |  |  |  |  |  |  |  |  |  |
| 0 | Vitvi14g04281\_t001 |  |  |  |  |  |  |  |  |  |  |  |  |
| 0 | Vitvi14g00669\_t001 |  |  |  |  |  |  |  |  |  |  |  |  |
| 0 | Vitvi14g04282\_t001 |  |  |  |  |  |  |  |  |  |  |  |  |
| 0 | Vitvi14g04283\_t001 |  |  |  |  |  |  |  |  |  |  |  |  |
| 0 | Vitvi14g00672\_t001 |  |  |  |  |  |  |  |  |  |  |  |  |
| 0 | Vitvi14g04284\_t001 |  |  |  |  |  |  |  |  |  |  |  |  |
| 0 | Vitvi14g00674\_t001 |  |  |  |  |  |  |  |  |  |  |  |  |
| 0 | Vitvi14g00675\_t001 |  |  |  |  |  |  |  |  |  |  |  |  |
| 0 | Vitvi14g00676\_t001 |  |  |  |  |  |  |  |  |  |  |  |  |
| 0 | Vitvi14g00679\_t001 |  |  |  |  |  |  |  |  |  |  |  |  |
| 0 | Vitvi14g04285\_t001 |  |  |  |  |  |  |  |  |  |  |  |  |
| 0 | Vitvi14g04286\_t001 |  |  |  |  |  |  |  |  |  |  |  |  |
| 0 | Vitvi14g04287\_t001 |  |  |  |  |  |  |  |  |  |  |  |  |
| 0 | Vitvi14g00685\_t001 |  |  |  |  |  |  |  |  |  |  |  |  |
| 0 | Vitvi14g04288\_t001 |  |  |  |  |  |  |  |  |  |  |  |  |
| 0 | Vitvi14g04289\_t001 |  |  |  |  |  |  |  |  |  |  |  |  |
| 0 | Vitvi14g04290\_t001 |  |  |  |  |  |  |  |  |  |  |  |  |
| 0 | Vitvi14g00695\_t001 |  |  |  |  |  |  |  |  |  |  |  |  |
| 0 | Vitvi14g00696\_t001 |  |  |  |  |  |  |  |  |  |  |  |  |
| 0 | Vitvi14g04291\_t001 |  |  |  |  |  |  |  |  |  |  |  |  |
| 0 | Vitvi14g04292\_t001 |  |  |  |  |  |  |  |  |  |  |  |  |
| 0 | Vitvi14g00700\_t001 |  |  |  |  |  |  |  |  |  |  |  |  |
| 0 | Vitvi14g04293\_t001 |  |  |  |  |  |  |  |  |  |  |  |  |
| 0 | Vitvi14g00701\_t001 |  |  |  |  |  |  |  |  |  |  |  |  |
| 0 | Vitvi14g02748\_t001 |  |  |  |  |  |  |  |  |  |  |  |  |
| 0 | Vitvi14g02749\_t001 |  |  |  |  |  |  |  |  |  |  |  |  |
| 0 | Vitvi14g04294\_t001 |  |  |  |  |  |  |  |  |  |  |  |  |
| 0 | Vitvi14g00707\_t001 |  |  |  |  |  |  |  |  |  |  |  |  |
| 0 | Vitvi14g04295\_t001 |  |  |  |  |  |  |  |  |  |  |  |  |
| 0 | Vitvi14g00708\_t001 |  |  |  |  |  |  |  |  |  |  |  |  |
| 1 | Vitvi14g04296\_t001 |  | Vitvi14g02608\_t001 |  |  |  |  |  |  |  |  |  |  |
| 1 | Vitvi14g00712\_t001 |  | \| \| |  |  |  |  |  |  |  |  |  |  |
| 1 | Vitvi14g04297\_t001 |  | \| \| |  |  |  |  |  |  |  |  |  |  |
| 1 | Vitvi14g00715\_t001 |  | \| \| |  |  |  |  |  |  |  |  |  |  |
| 1 | Vitvi14g00716\_t001 |  | \| \| |  |  |  |  |  |  |  |  |  |  |
| 1 | Vitvi14g04298\_t001 |  | \| \| |  |  |  |  |  |  |  |  |  |  |
| 1 | Vitvi14g04299\_t001 |  | Vitvi14g02617\_t001 |  |  |  |  |  |  |  |  |  |  |
| 2 | Vitvi14g04300\_t001 |  | Vitvi14g00363\_t001 |  | Vitvi14g02633\_t001 |  |  |  |  |  |  |  |  |
| 2 | Vitvi14g02754\_t001 |  | \| \| |  | \| \| |  |  |  |  |  |  |  |  |
| 2 | Vitvi14g02755\_t001 |  | \| \| |  | \| \| |  |  |  |  |  |  |  |  |
| 2 | Vitvi14g04301\_t001 |  | \| \| |  | Vitvi14g04153\_t001 |  |  |  |  |  |  |  |  |
| 2 | Vitvi14g02757\_t001 |  | \| \| |  | \| \| |  |  |  |  |  |  |  |  |
| 2 | Vitvi14g02758\_t001 |  | \| \| |  | Vitvi14g00374\_t001 |  |  |  |  |  |  |  |  |
| 2 | Vitvi14g04302\_t001 |  | \| \| |  | \| \| |  |  |  |  |  |  |  |  |
| 2 | Vitvi14g00729\_t001 |  | Vitvi14g04151\_t001 |  | Vitvi14g04151\_t001 |  |  |  |  |  |  |  |  |
| 2 | Vitvi14g04303\_t001 |  | \| \| |  | \| \| |  |  |  |  |  |  |  |  |
| 2 | Vitvi14g02761\_t001 |  | Vitvi14g04153\_t001 |  | \| \| |  |  |  |  |  |  |  |  |
| 2 | Vitvi14g02762\_t001 |  | \| \| |  | Vitvi14g04148\_t001 |  |  |  |  |  |  |  |  |
| 2 | Vitvi14g04304\_t001 |  | \| \| |  | \| \| |  |  |  |  |  |  |  |  |
| 2 | Vitvi14g04306\_t001 |  | \| \| |  | \| \| |  |  |  |  |  |  |  |  |
| 2 | Vitvi14g00735\_t001 |  | \| \| |  | \| \| |  |  |  |  |  |  |  |  |
| 2 | Vitvi14g00736\_t001 |  | \| \| |  | \| \| |  |  |  |  |  |  |  |  |
| 2 | Vitvi14g00737\_t001 |  | \| \| |  | \| \| |  |  |  |  |  |  |  |  |
| 2 | Vitvi14g00738\_t001 |  | \| \| |  | \| \| |  |  |  |  |  |  |  |  |
| 2 | Vitvi14g00742\_t001 |  | \| \| |  | \| \| |  |  |  |  |  |  |  |  |
| 2 | Vitvi14g00743\_t003 |  | \| \| |  | \| \| |  |  |  |  |  |  |  |  |
| 2 | Vitvi14g00746\_t001 |  | \| \| |  | \| \| |  |  |  |  |  |  |  |  |
| 2 | Vitvi14g00747\_t001 |  | \| \| |  | \| \| |  |  |  |  |  |  |  |  |
| 2 | Vitvi14g04307\_t001 |  | \| \| |  | \| \| |  |  |  |  |  |  |  |  |
| 2 | Vitvi14g04308\_t001 |  | Vitvi14g00377\_t001 |  | Vitvi14g00366\_t001 |  |  |  |  |  |  |  |  |
| 0 | Vitvi14g04309\_t001 |  |  |  |  |  |  |  |  |  |  |  |  |
| 0 | Vitvi14g04310\_t001 |  |  |  |  |  |  |  |  |  |  |  |  |
| 0 | Vitvi14g04311\_t001 |  |  |  |  |  |  |  |  |  |  |  |  |
| 0 | Vitvi14g00753\_t001 |  |  |  |  |  |  |  |  |  |  |  |  |
| 0 | Vitvi14g00754\_t001 |  |  |  |  |  |  |  |  |  |  |  |  |
| 0 | Vitvi14g00755\_t001 |  |  |  |  |  |  |  |  |  |  |  |  |
| 0 | Vitvi14g04312\_t001 |  |  |  |  |  |  |  |  |  |  |  |  |
| 0 | Vitvi14g04313\_t001 |  |  |  |  |  |  |  |  |  |  |  |  |
| 0 | Vitvi14g04314\_t001 |  |  |  |  |  |  |  |  |  |  |  |  |
| 0 | Vitvi14g04315\_t001 |  |  |  |  |  |  |  |  |  |  |  |  |
| 0 | Vitvi14g04316\_t001 |  |  |  |  |  |  |  |  |  |  |  |  |
| 0 | Vitvi14g00760\_t001 |  |  |  |  |  |  |  |  |  |  |  |  |
| 0 | Vitvi14g00762\_t001 |  |  |  |  |  |  |  |  |  |  |  |  |
| 0 | Vitvi14g00763\_t001 |  |  |  |  |  |  |  |  |  |  |  |  |
| 0 | Vitvi14g00768\_t001 |  |  |  |  |  |  |  |  |  |  |  |  |
| 0 | Vitvi14g04317\_t001 |  |  |  |  |  |  |  |  |  |  |  |  |
| 0 | Vitvi14g04318\_t001 |  |  |  |  |  |  |  |  |  |  |  |  |
| 0 | Vitvi14g00770\_t001 |  |  |  |  |  |  |  |  |  |  |  |  |
| 0 | Vitvi14g00772\_t001 |  |  |  |  |  |  |  |  |  |  |  |  |
| 0 | Vitvi14g04319\_t001 |  |  |  |  |  |  |  |  |  |  |  |  |
| 0 | Vitvi14g02773\_t001 |  |  |  |  |  |  |  |  |  |  |  |  |
| 0 | Vitvi14g04320\_t001 |  |  |  |  |  |  |  |  |  |  |  |  |
| 0 | Vitvi14g00774\_t001 |  |  |  |  |  |  |  |  |  |  |  |  |
| 0 | Vitvi14g00775\_t001 |  |  |  |  |  |  |  |  |  |  |  |  |
| 0 | Vitvi14g04321\_t001 |  |  |  |  |  |  |  |  |  |  |  |  |
| 0 | Vitvi14g04322\_t001 |  |  |  |  |  |  |  |  |  |  |  |  |
| 0 | Vitvi14g04323\_t001 |  |  |  |  |  |  |  |  |  |  |  |  |
| 0 | Vitvi14g04324\_t001 |  |  |  |  |  |  |  |  |  |  |  |  |
| 0 | Vitvi14g00783\_t001 |  |  |  |  |  |  |  |  |  |  |  |  |
| 0 | Vitvi14g00786\_t001 |  |  |  |  |  |  |  |  |  |  |  |  |
| 0 | Vitvi14g00789\_t001 |  |  |  |  |  |  |  |  |  |  |  |  |
| 0 | Vitvi14g00790\_t001 |  |  |  |  |  |  |  |  |  |  |  |  |
| 0 | Vitvi14g00791\_t001 |  |  |  |  |  |  |  |  |  |  |  |  |
| 0 | Vitvi14g00793\_t001 |  |  |  |  |  |  |  |  |  |  |  |  |
| 0 | Vitvi14g04325\_t001 |  |  |  |  |  |  |  |  |  |  |  |  |
| 0 | Vitvi14g00796\_t001 |  |  |  |  |  |  |  |  |  |  |  |  |
| 0 | Vitvi14g00797\_t001 |  |  |  |  |  |  |  |  |  |  |  |  |
| 0 | Vitvi14g04326\_t001 |  |  |  |  |  |  |  |  |  |  |  |  |
| 0 | Vitvi14g00798\_t001 |  |  |  |  |  |  |  |  |  |  |  |  |
| 0 | Vitvi14g04327\_t001 |  |  |  |  |  |  |  |  |  |  |  |  |
| 0 | Vitvi14g02784\_t001 |  |  |  |  |  |  |  |  |  |  |  |  |
| 0 | Vitvi14g00800\_t001 |  |  |  |  |  |  |  |  |  |  |  |  |
| 0 | Vitvi14g00802\_t001 |  |  |  |  |  |  |  |  |  |  |  |  |
| 0 | Vitvi14g00803\_t001 |  |  |  |  |  |  |  |  |  |  |  |  |
| 0 | Vitvi14g02430\_t001 |  |  |  |  |  |  |  |  |  |  |  |  |
| 0 | Vitvi14g04328\_t001 |  |  |  |  |  |  |  |  |  |  |  |  |
| 0 | Vitvi14g04329\_t001 |  |  |  |  |  |  |  |  |  |  |  |  |
| 0 | Vitvi14g00821\_t001 |  |  |  |  |  |  |  |  |  |  |  |  |
| 0 | Vitvi14g00827\_t001 |  |  |  |  |  |  |  |  |  |  |  |  |
| 0 | Vitvi14g04330\_t001 |  |  |  |  |  |  |  |  |  |  |  |  |
| 0 | Vitvi14g00831\_t001 |  |  |  |  |  |  |  |  |  |  |  |  |
| 0 | Vitvi14g04331\_t001 |  |  |  |  |  |  |  |  |  |  |  |  |
| 0 | Vitvi14g04332\_t001 |  |  |  |  |  |  |  |  |  |  |  |  |
| 0 | Vitvi14g04333\_t001 |  |  |  |  |  |  |  |  |  |  |  |  |
| 0 | Vitvi14g04334\_t001 |  |  |  |  |  |  |  |  |  |  |  |  |
| 0 | Vitvi14g04335\_t001 |  |  |  |  |  |  |  |  |  |  |  |  |
| 0 | Vitvi14g04336\_t001 |  |  |  |  |  |  |  |  |  |  |  |  |
| 0 | Vitvi14g00840\_t001 |  |  |  |  |  |  |  |  |  |  |  |  |
| 0 | Vitvi14g00841\_t001 |  |  |  |  |  |  |  |  |  |  |  |  |
| 0 | Vitvi14g00842\_t001 |  |  |  |  |  |  |  |  |  |  |  |  |
| 0 | Vitvi14g00845\_t001 |  |  |  |  |  |  |  |  |  |  |  |  |
| 0 | Vitvi14g00846\_t001 |  |  |  |  |  |  |  |  |  |  |  |  |
| 0 | Vitvi14g04337\_t001 |  |  |  |  |  |  |  |  |  |  |  |  |
| 0 | Vitvi14g04338\_t001 |  |  |  |  |  |  |  |  |  |  |  |  |
| 0 | Vitvi14g04339\_t001 |  |  |  |  |  |  |  |  |  |  |  |  |
| 0 | Vitvi14g04340\_t001 |  |  |  |  |  |  |  |  |  |  |  |  |
| 0 | Vitvi14g04341\_t001 |  |  |  |  |  |  |  |  |  |  |  |  |
| 0 | Vitvi14g00862\_t001 |  |  |  |  |  |  |  |  |  |  |  |  |
| 0 | Vitvi14g04342\_t001 |  |  |  |  |  |  |  |  |  |  |  |  |
| 0 | Vitvi14g04343\_t001 |  |  |  |  |  |  |  |  |  |  |  |  |
| 0 | Vitvi14g04344\_t001 |  |  |  |  |  |  |  |  |  |  |  |  |
| 0 | Vitvi14g04345\_t001 |  |  |  |  |  |  |  |  |  |  |  |  |
| 0 | Vitvi14g00871\_t001 |  |  |  |  |  |  |  |  |  |  |  |  |
| 0 | Vitvi14g04346\_t001 |  |  |  |  |  |  |  |  |  |  |  |  |
| 0 | Vitvi14g04347\_t001 |  |  |  |  |  |  |  |  |  |  |  |  |
| 0 | Vitvi14g00873\_t001 |  |  |  |  |  |  |  |  |  |  |  |  |
| 0 | Vitvi14g00874\_t001 |  |  |  |  |  |  |  |  |  |  |  |  |
| 0 | Vitvi14g04348\_t001 |  |  |  |  |  |  |  |  |  |  |  |  |
| 0 | Vitvi14g04349\_t001 |  |  |  |  |  |  |  |  |  |  |  |  |
| 0 | Vitvi14g00875\_t001 |  |  |  |  |  |  |  |  |  |  |  |  |
| 0 | Vitvi14g00877\_t001 |  |  |  |  |  |  |  |  |  |  |  |  |
| 0 | Vitvi14g04350\_t001 |  |  |  |  |  |  |  |  |  |  |  |  |
| 0 | Vitvi14g00879\_t001 |  |  |  |  |  |  |  |  |  |  |  |  |
| 0 | Vitvi14g00881\_t001 |  |  |  |  |  |  |  |  |  |  |  |  |
| 0 | Vitvi14g04351\_t001 |  |  |  |  |  |  |  |  |  |  |  |  |
| 0 | Vitvi14g00883\_t001 |  |  |  |  |  |  |  |  |  |  |  |  |
| 0 | Vitvi14g02810\_t001 |  |  |  |  |  |  |  |  |  |  |  |  |
| 0 | Vitvi14g04352\_t001 |  |  |  |  |  |  |  |  |  |  |  |  |
| 0 | Vitvi14g04353\_t001 |  |  |  |  |  |  |  |  |  |  |  |  |
| 0 | Vitvi14g00887\_t001 |  |  |  |  |  |  |  |  |  |  |  |  |
| 0 | Vitvi14g00888\_t003 |  |  |  |  |  |  |  |  |  |  |  |  |
| 0 | Vitvi14g04354\_t001 |  |  |  |  |  |  |  |  |  |  |  |  |
| 0 | Vitvi14g04355\_t001 |  |  |  |  |  |  |  |  |  |  |  |  |
| 0 | Vitvi14g04356\_t001 |  |  |  |  |  |  |  |  |  |  |  |  |
| 0 | Vitvi14g00891\_t001 |  |  |  |  |  |  |  |  |  |  |  |  |
| 0 | Vitvi14g04357\_t001 |  |  |  |  |  |  |  |  |  |  |  |  |
| 0 | Vitvi14g04358\_t001 |  |  |  |  |  |  |  |  |  |  |  |  |
| 0 | Vitvi14g00893\_t001 |  |  |  |  |  |  |  |  |  |  |  |  |
| 0 | Vitvi14g00894\_t001 |  |  |  |  |  |  |  |  |  |  |  |  |
| 0 | Vitvi14g02812\_t001 |  |  |  |  |  |  |  |  |  |  |  |  |
| 0 | Vitvi14g00896\_t001 |  |  |  |  |  |  |  |  |  |  |  |  |
| 0 | Vitvi14g04359\_t001 |  |  |  |  |  |  |  |  |  |  |  |  |
| 0 | Vitvi14g00900\_t001 |  |  |  |  |  |  |  |  |  |  |  |  |
| 0 | Vitvi14g00901\_t001 |  |  |  |  |  |  |  |  |  |  |  |  |
| 0 | Vitvi14g04360\_t001 |  |  |  |  |  |  |  |  |  |  |  |  |
| 0 | Vitvi14g04361\_t001 |  |  |  |  |  |  |  |  |  |  |  |  |
| 0 | Vitvi14g04362\_t001 |  |  |  |  |  |  |  |  |  |  |  |  |
| 0 | Vitvi14g04363\_t001 |  |  |  |  |  |  |  |  |  |  |  |  |
| 0 | Vitvi14g00902\_t001 |  |  |  |  |  |  |  |  |  |  |  |  |
| 0 | Vitvi14g04364\_t001 |  |  |  |  |  |  |  |  |  |  |  |  |
| 0 | Vitvi14g00907\_t001 |  |  |  |  |  |  |  |  |  |  |  |  |
| 0 | Vitvi14g04365\_t001 |  |  |  |  |  |  |  |  |  |  |  |  |
| 0 | Vitvi14g04366\_t001 |  |  |  |  |  |  |  |  |  |  |  |  |
| 0 | Vitvi14g00910\_t001 |  |  |  |  |  |  |  |  |  |  |  |  |
| 0 | Vitvi14g02815\_t001 |  |  |  |  |  |  |  |  |  |  |  |  |
| 0 | Vitvi14g00914\_t001 |  |  |  |  |  |  |  |  |  |  |  |  |
| 0 | Vitvi14g04367\_t001 |  |  |  |  |  |  |  |  |  |  |  |  |
| 0 | Vitvi14g00919\_t001 |  |  |  |  |  |  |  |  |  |  |  |  |
| 0 | Vitvi14g04368\_t001 |  |  |  |  |  |  |  |  |  |  |  |  |
| 0 | Vitvi14g00920\_t001 |  |  |  |  |  |  |  |  |  |  |  |  |
| 0 | Vitvi14g00925\_t001 |  |  |  |  |  |  |  |  |  |  |  |  |
| 0 | Vitvi14g00930\_t001 |  |  |  |  |  |  |  |  |  |  |  |  |
| 0 | Vitvi14g04369\_t001 |  |  |  |  |  |  |  |  |  |  |  |  |
| 0 | Vitvi14g04370\_t001 |  |  |  |  |  |  |  |  |  |  |  |  |
| 0 | Vitvi14g04371\_t001 |  |  |  |  |  |  |  |  |  |  |  |  |
| 0 | Vitvi14g04372\_t001 |  |  |  |  |  |  |  |  |  |  |  |  |
| 0 | Vitvi14g04373\_t001 |  |  |  |  |  |  |  |  |  |  |  |  |
| 0 | Vitvi14g00940\_t001 |  |  |  |  |  |  |  |  |  |  |  |  |
| 0 | Vitvi14g04374\_t001 |  |  |  |  |  |  |  |  |  |  |  |  |
| 0 | Vitvi14g04375\_t001 |  |  |  |  |  |  |  |  |  |  |  |  |
| 0 | Vitvi14g04376\_t001 |  |  |  |  |  |  |  |  |  |  |  |  |
| 0 | Vitvi14g04377\_t001 |  |  |  |  |  |  |  |  |  |  |  |  |
| 0 | Vitvi14g04378\_t001 |  |  |  |  |  |  |  |  |  |  |  |  |
| 0 | Vitvi14g04379\_t001 |  |  |  |  |  |  |  |  |  |  |  |  |
| 0 | Vitvi14g00948\_t001 |  |  |  |  |  |  |  |  |  |  |  |  |
| 0 | Vitvi14g02821\_t001 |  |  |  |  |  |  |  |  |  |  |  |  |
| 0 | Vitvi14g00949\_t001 |  |  |  |  |  |  |  |  |  |  |  |  |
| 0 | Vitvi14g00950\_t001 |  |  |  |  |  |  |  |  |  |  |  |  |
| 0 | Vitvi14g00952\_t001 |  |  |  |  |  |  |  |  |  |  |  |  |
| 0 | Vitvi14g02823\_t001 |  |  |  |  |  |  |  |  |  |  |  |  |
| 0 | Vitvi14g00953\_t001 |  |  |  |  |  |  |  |  |  |  |  |  |
| 0 | Vitvi14g00954\_t002 |  |  |  |  |  |  |  |  |  |  |  |  |
| 0 | Vitvi14g04380\_t001 |  |  |  |  |  |  |  |  |  |  |  |  |
| 0 | Vitvi14g04381\_t001 |  |  |  |  |  |  |  |  |  |  |  |  |
| 0 | Vitvi14g00956\_t001 |  |  |  |  |  |  |  |  |  |  |  |  |
| 0 | Vitvi14g00957\_t001 |  |  |  |  |  |  |  |  |  |  |  |  |
| 0 | Vitvi14g00958\_t001 |  |  |  |  |  |  |  |  |  |  |  |  |
| 0 | Vitvi14g04382\_t001 |  |  |  |  |  |  |  |  |  |  |  |  |
| 0 | Vitvi14g04383\_t001 |  |  |  |  |  |  |  |  |  |  |  |  |
| 0 | Vitvi14g00961\_t001 |  |  |  |  |  |  |  |  |  |  |  |  |
| 0 | Vitvi14g00964\_t001 |  |  |  |  |  |  |  |  |  |  |  |  |
| 0 | Vitvi14g04384\_t001 |  |  |  |  |  |  |  |  |  |  |  |  |
| 0 | Vitvi14g04385\_t001 |  |  |  |  |  |  |  |  |  |  |  |  |
| 0 | Vitvi14g00966\_t001 |  |  |  |  |  |  |  |  |  |  |  |  |
| 0 | Vitvi14g04386\_t001 |  |  |  |  |  |  |  |  |  |  |  |  |
| 0 | Vitvi14g00969\_t001 |  |  |  |  |  |  |  |  |  |  |  |  |
| 1 | Vitvi14g02829\_t001 |  | Vitvi01g01971\_t001 |  |  |  |  |  |  |  |  |  |  |
| 1 | Vitvi14g04387\_t001 |  | \| \| |  |  |  |  |  |  |  |  |  |  |
| 1 | Vitvi14g00972\_t001 |  | \| \| |  |  |  |  |  |  |  |  |  |  |
| 1 | Vitvi14g00973\_t001 |  | Vitvi01g00403\_t001 |  |  |  |  |  |  |  |  |  |  |
| 1 | Vitvi14g04388\_t001 |  | \| \| |  |  |  |  |  |  |  |  |  |  |
| 1 | Vitvi14g00974\_t001 |  | Vitvi01g00401\_t001 |  |  |  |  |  |  |  |  |  |  |
| 1 | Vitvi14g00977\_t001 |  | \| \| |  |  |  |  |  |  |  |  |  |  |
| 1 | Vitvi14g00980\_t001 |  | \| \| |  |  |  |  |  |  |  |  |  |  |
| 1 | Vitvi14g04389\_t001 |  | \| \| |  |  |  |  |  |  |  |  |  |  |
| 1 | Vitvi14g00986\_t001 |  | \| \| |  |  |  |  |  |  |  |  |  |  |
| 1 | Vitvi14g02834\_t001 |  | \| \| |  |  |  |  |  |  |  |  |  |  |
| 1 | Vitvi14g00987\_t001 |  | \| \| |  |  |  |  |  |  |  |  |  |  |
| 1 | Vitvi14g04390\_t001 |  | \| \| |  |  |  |  |  |  |  |  |  |  |
| 1 | Vitvi14g00992\_t001 |  | \| \| |  |  |  |  |  |  |  |  |  |  |
| 1 | Vitvi14g00994\_t001 |  | \| \| |  |  |  |  |  |  |  |  |  |  |
| 1 | Vitvi14g00995\_t001 |  | Vitvi01g00394\_t001 |  |  |  |  |  |  |  |  |  |  |
| 1 | Vitvi14g00996\_t001 |  | \| \| |  |  |  |  |  |  |  |  |  |  |
| 1 | Vitvi14g00997\_t001 |  | Vitvi01g00393\_t001 |  |  |  |  |  |  |  |  |  |  |
| 1 | Vitvi14g02836\_t001 |  | \| \| |  |  |  |  |  |  |  |  |  |  |
| 1 | Vitvi14g00998\_t001 |  | \| \| |  |  |  |  |  |  |  |  |  |  |
| 1 | Vitvi14g04391\_t001 |  | \| \| |  |  |  |  |  |  |  |  |  |  |
| 1 | Vitvi14g00999\_t001 |  | \| \| |  |  |  |  |  |  |  |  |  |  |
| 1 | Vitvi14g04392\_t001 |  | \| \| |  |  |  |  |  |  |  |  |  |  |
| 1 | Vitvi14g01002\_t001 |  | \| \| |  |  |  |  |  |  |  |  |  |  |
| 1 | Vitvi14g01003\_t002 |  | \| \| |  |  |  |  |  |  |  |  |  |  |
| 1 | Vitvi14g01004\_t001 |  | \| \| |  |  |  |  |  |  |  |  |  |  |
| 1 | Vitvi14g01005\_t001 |  | \| \| |  |  |  |  |  |  |  |  |  |  |
| 1 | Vitvi14g01006\_t001 |  | \| \| |  |  |  |  |  |  |  |  |  |  |
| 1 | Vitvi14g01007\_t001 |  | \| \| |  |  |  |  |  |  |  |  |  |  |
| 1 | Vitvi14g04393\_t001 |  | \| \| |  |  |  |  |  |  |  |  |  |  |
| 1 | Vitvi14g01011\_t002 |  | \| \| |  |  |  |  |  |  |  |  |  |  |
| 1 | Vitvi14g02841\_t001 |  | Vitvi01g00388\_t001 |  |  |  |  |  |  |  |  |  |  |
| 1 | Vitvi14g01012\_t001 |  | \| \| |  |  |  |  |  |  |  |  |  |  |
| 1 | Vitvi14g02844\_t001 |  | \| \| |  |  |  |  |  |  |  |  |  |  |
| 1 | Vitvi14g02845\_t001 |  | \| \| |  |  |  |  |  |  |  |  |  |  |
| 1 | Vitvi14g04394\_t001 |  | \| \| |  |  |  |  |  |  |  |  |  |  |
| 1 | Vitvi14g01018\_t001 |  | \| \| |  |  |  |  |  |  |  |  |  |  |
| 1 | Vitvi14g01023\_t003 |  | Vitvi01g00386\_t001 |  |  |  |  |  |  |  |  |  |  |
| 1 | Vitvi14g04395\_t001 |  | \| \| |  |  |  |  |  |  |  |  |  |  |
| 1 | Vitvi14g04396\_t001 |  | \| \| |  |  |  |  |  |  |  |  |  |  |
| 1 | Vitvi14g04397\_t001 |  | \| \| |  |  |  |  |  |  |  |  |  |  |
| 1 | Vitvi14g01025\_t001 |  | Vitvi01g00381\_t002 |  |  |  |  |  |  |  |  |  |  |
| 1 | Vitvi14g01026\_t001 |  | \| \| |  |  |  |  |  |  |  |  |  |  |
| 1 | Vitvi14g04398\_t001 |  | \| \| |  |  |  |  |  |  |  |  |  |  |
| 1 | Vitvi14g04399\_t001 |  | \| \| |  |  |  |  |  |  |  |  |  |  |
| 1 | Vitvi14g01031\_t001 |  | \| \| |  |  |  |  |  |  |  |  |  |  |
| 1 | Vitvi14g01032\_t001 |  | \| \| |  |  |  |  |  |  |  |  |  |  |
| 1 | Vitvi14g01033\_t001 |  | \| \| |  |  |  |  |  |  |  |  |  |  |
| 1 | Vitvi14g02849\_t001 |  | \| \| |  |  |  |  |  |  |  |  |  |  |
| 1 | Vitvi14g02850\_t001 |  | Vitvi01g01967\_t001 |  |  |  |  |  |  |  |  |  |  |
| 1 | Vitvi14g02851\_t001 |  | \| \| |  |  |  |  |  |  |  |  |  |  |
| 1 | Vitvi14g02852\_t001 |  | \| \| |  |  |  |  |  |  |  |  |  |  |
| 1 | Vitvi14g01035\_t001 |  | Vitvi01g00380\_t001 |  |  |  |  |  |  |  |  |  |  |
| 1 | Vitvi14g01037\_t001 |  | Vitvi01g00379\_t001 |  |  |  |  |  |  |  |  |  |  |
| 1 | Vitvi14g01040\_t001 |  | \| \| |  |  |  |  |  |  |  |  |  |  |
| 1 | Vitvi14g01042\_t001 |  | \| \| |  |  |  |  |  |  |  |  |  |  |
| 1 | Vitvi14g04400\_t001 |  | \| \| |  |  |  |  |  |  |  |  |  |  |
| 1 | Vitvi14g04401\_t001 |  | \| \| |  |  |  |  |  |  |  |  |  |  |
| 1 | Vitvi14g01044\_t001 |  | \| \| |  |  |  |  |  |  |  |  |  |  |
| 1 | Vitvi14g01046\_t001 |  | \| \| |  |  |  |  |  |  |  |  |  |  |
| 1 | Vitvi14g04402\_t001 |  | \| \| |  |  |  |  |  |  |  |  |  |  |
| 1 | Vitvi14g01048\_t001 |  | \| \| |  |  |  |  |  |  |  |  |  |  |
| 1 | Vitvi14g04403\_t001 |  | \| \| |  |  |  |  |  |  |  |  |  |  |
| 1 | Vitvi14g01050\_t001 |  | \| \| |  |  |  |  |  |  |  |  |  |  |
| 1 | Vitvi14g04404\_t001 |  | \| \| |  |  |  |  |  |  |  |  |  |  |
| 2 | Vitvi14g01051\_t001 |  | \| \| |  | Vitvi17g01378\_t001 |  |  |  |  |  |  |  |  |
| 2 | Vitvi14g01052\_t001 |  | \| \| |  | \| \| |  |  |  |  |  |  |  |  |
| 2 | Vitvi14g02857\_t001 |  | \| \| |  | \| \| |  |  |  |  |  |  |  |  |
| 2 | Vitvi14g01054\_t001 |  | \| \| |  | \| \| |  |  |  |  |  |  |  |  |
| 2 | Vitvi14g01055\_t001 |  | \| \| |  | \| \| |  |  |  |  |  |  |  |  |
| 2 | Vitvi14g01057\_t001 |  | Vitvi01g00372\_t001 |  | \| \| |  |  |  |  |  |  |  |  |
| 2 | Vitvi14g04405\_t001 |  | \| \| |  | \| \| |  |  |  |  |  |  |  |  |
| 2 | Vitvi14g01060\_t001 |  | \| \| |  | \| \| |  |  |  |  |  |  |  |  |
| 2 | Vitvi14g01061\_t002 |  | \| \| |  | \| \| |  |  |  |  |  |  |  |  |
| 2 | Vitvi14g04406\_t001 |  | \| \| |  | \| \| |  |  |  |  |  |  |  |  |
| 2 | Vitvi14g02858\_t001 |  | \| \| |  | \| \| |  |  |  |  |  |  |  |  |
| 2 | Vitvi14g01062\_t001 |  | Vitvi01g00370\_t001 |  | Vitvi17g00258\_t002 |  |  |  |  |  |  |  |  |
| 2 | Vitvi14g01064\_t001 |  | \| \| |  | \| \| |  |  |  |  |  |  |  |  |
| 2 | Vitvi14g01066\_t001 |  | \| \| |  | \| \| |  |  |  |  |  |  |  |  |
| 2 | Vitvi14g04407\_t001 |  | \| \| |  | \| \| |  |  |  |  |  |  |  |  |
| 2 | Vitvi14g01067\_t001 |  | \| \| |  | \| \| |  |  |  |  |  |  |  |  |
| 2 | Vitvi14g01068\_t001 |  | \| \| |  | \| \| |  |  |  |  |  |  |  |  |
| 2 | Vitvi14g04408\_t001 |  | \| \| |  | \| \| |  |  |  |  |  |  |  |  |
| 2 | Vitvi14g04409\_t001 |  | \| \| |  | \| \| |  |  |  |  |  |  |  |  |
| 2 | Vitvi14g04410\_t001 |  | \| \| |  | \| \| |  |  |  |  |  |  |  |  |
| 2 | Vitvi14g01114\_t001 |  | Vitvi01g00347\_t001 |  | \| \| |  |  |  |  |  |  |  |  |
| 2 | Vitvi14g02866\_t001 |  | Vitvi01g01958\_t001 |  | Vitvi17g04071\_t001 |  |  |  |  |  |  |  |  |
| 2 | Vitvi14g01118\_t001 |  | \| \| |  | \| \| |  |  |  |  |  |  |  |  |
| 2 | Vitvi14g01119\_t001 |  | Vitvi01g01956\_t001 |  | Vitvi17g00273\_t001 |  |  |  |  |  |  |  |  |
| 2 | Vitvi14g01120\_t002 |  | Vitvi01g01955\_t001 |  | Vitvi17g00274\_t001 |  |  |  |  |  |  |  |  |
| 2 | Vitvi14g04411\_t001 |  | \| \| |  | \| \| |  |  |  |  |  |  |  |  |
| 2 | Vitvi14g04412\_t001 |  | \| \| |  | \| \| |  |  |  |  |  |  |  |  |
| 2 | Vitvi14g01122\_t001 |  | \| \| |  | \| \| |  |  |  |  |  |  |  |  |
| 2 | Vitvi14g04413\_t001 |  | \| \| |  | \| \| |  |  |  |  |  |  |  |  |
| 2 | Vitvi14g01124\_t001 |  | \| \| |  | \| \| |  |  |  |  |  |  |  |  |
| 2 | Vitvi14g01125\_t001 |  | \| \| |  | \| \| |  |  |  |  |  |  |  |  |
| 2 | Vitvi14g02869\_t001 |  | Vitvi01g00331\_t001 |  | Vitvi17g00283\_t001 |  |  |  |  |  |  |  |  |
| 2 | Vitvi14g04414\_t001 |  | \| \| |  | \| \| |  |  |  |  |  |  |  |  |
| 2 | Vitvi14g01128\_t001 |  | \| \| |  | \| \| |  |  |  |  |  |  |  |  |
| 2 | Vitvi14g04415\_t001 |  | \| \| |  | \| \| |  |  |  |  |  |  |  |  |
| 2 | Vitvi14g04416\_t001 |  | \| \| |  | \| \| |  |  |  |  |  |  |  |  |
| 2 | Vitvi14g04417\_t001 |  | \| \| |  | \| \| |  |  |  |  |  |  |  |  |
| 2 | Vitvi14g04418\_t001 |  | \| \| |  | \| \| |  |  |  |  |  |  |  |  |
| 2 | Vitvi14g01133\_t001 |  | \| \| |  | \| \| |  |  |  |  |  |  |  |  |
| 2 | Vitvi14g04419\_t001 |  | Vitvi01g04083\_t002 |  | Vitvi17g00289\_t001 |  |  |  |  |  |  |  |  |
| 2 | Vitvi14g01139\_t001 |  | Vitvi01g01949\_t001 |  | \| \| |  |  |  |  |  |  |  |  |
| 2 | Vitvi14g01141\_t001 |  | \| \| |  | \| \| |  |  |  |  |  |  |  |  |
| 2 | Vitvi14g01143\_t001 |  | \| \| |  | \| \| |  |  |  |  |  |  |  |  |
| 2 | Vitvi14g01144\_t001 |  | \| \| |  | \| \| |  |  |  |  |  |  |  |  |
| 2 | Vitvi14g04420\_t001 |  | Vitvi01g04079\_t001 |  | \| \| |  |  |  |  |  |  |  |  |
| 2 | Vitvi14g04421\_t001 |  | \| \| |  | \| \| |  |  |  |  |  |  |  |  |
| 2 | Vitvi14g04422\_t001 |  | \| \| |  | \| \| |  |  |  |  |  |  |  |  |
| 2 | Vitvi14g04423\_t001 |  | \| \| |  | \| \| |  |  |  |  |  |  |  |  |
| 2 | Vitvi14g04424\_t001 |  | \| \| |  | \| \| |  |  |  |  |  |  |  |  |
| 2 | Vitvi14g04425\_t001 |  | \| \| |  | Vitvi17g04080\_t001 |  |  |  |  |  |  |  |  |
| 2 | Vitvi14g04426\_t001 |  | \| \| |  | \| \| |  |  |  |  |  |  |  |  |
| 2 | Vitvi14g01151\_t001 |  | \| \| |  | \| \| |  |  |  |  |  |  |  |  |
| 2 | Vitvi14g01152\_t001 |  | Vitvi01g00319\_t002 |  | \| \| |  |  |  |  |  |  |  |  |
| 2 | Vitvi14g01156\_t001 |  | Vitvi01g00315\_t001 |  | Vitvi17g00294\_t001 |  |  |  |  |  |  |  |  |
| 2 | Vitvi14g02871\_t001 |  | \| \| |  | \| \| |  |  |  |  |  |  |  |  |
| 2 | Vitvi14g01157\_t001 |  | \| \| |  | \| \| |  |  |  |  |  |  |  |  |
| 2 | Vitvi14g02872\_t001 |  | Vitvi01g00309\_t001 |  | \| \| |  |  |  |  |  |  |  |  |
| 2 | Vitvi14g01158\_t001 |  | \| \| |  | \| \| |  |  |  |  |  |  |  |  |
| 2 | Vitvi14g04427\_t001 |  | \| \| |  | \| \| |  |  |  |  |  |  |  |  |
| 2 | Vitvi14g01160\_t001 |  | \| \| |  | \| \| |  |  |  |  |  |  |  |  |
| 2 | Vitvi14g01161\_t001 |  | \| \| |  | \| \| |  |  |  |  |  |  |  |  |
| 2 | Vitvi14g01162\_t001 |  | \| \| |  | \| \| |  |  |  |  |  |  |  |  |
| 2 | Vitvi14g04428\_t001 |  | \| \| |  | \| \| |  |  |  |  |  |  |  |  |
| 2 | Vitvi14g01164\_t001 |  | Vitvi01g00305\_t001 |  | Vitvi17g00300\_t001 |  |  |  |  |  |  |  |  |
| 2 | Vitvi14g01165\_t001 |  | \| \| |  | \| \| |  |  |  |  |  |  |  |  |
| 2 | Vitvi14g01166\_t001 |  | \| \| |  | \| \| |  |  |  |  |  |  |  |  |
| 2 | Vitvi14g02874\_t001 |  | \| \| |  | \| \| |  |  |  |  |  |  |  |  |
| 2 | Vitvi14g01170\_t001 |  | \| \| |  | \| \| |  |  |  |  |  |  |  |  |
| 2 | Vitvi14g01171\_t001 |  | \| \| |  | \| \| |  |  |  |  |  |  |  |  |
| 2 | Vitvi14g01174\_t001 |  | \| \| |  | Vitvi17g00303\_t001 |  |  |  |  |  |  |  |  |
| 2 | Vitvi14g01176\_t001 |  | \| \| |  | Vitvi17g00304\_t001 |  |  |  |  |  |  |  |  |
| 2 | Vitvi14g01177\_t001 |  | \| \| |  | \| \| |  |  |  |  |  |  |  |  |
| 2 | Vitvi14g01178\_t001 |  | Vitvi01g04074\_t001 |  | \| \| |  |  |  |  |  |  |  |  |
| 2 | Vitvi14g01179\_t001 |  | \| \| |  | Vitvi17g00305\_t001 |  |  |  |  |  |  |  |  |
| 2 | Vitvi14g01180\_t001 |  | \| \| |  | \| \| |  |  |  |  |  |  |  |  |
| 2 | Vitvi14g01181\_t001 |  | Vitvi01g01946\_t001 |  | Vitvi17g00311\_t001 |  |  |  |  |  |  |  |  |
| 2 | Vitvi14g04429\_t001 |  | \| \| |  | \| \| |  |  |  |  |  |  |  |  |
| 2 | Vitvi14g02875\_t001 |  | \| \| |  | \| \| |  |  |  |  |  |  |  |  |
| 2 | Vitvi14g01184\_t001 |  | \| \| |  | \| \| |  |  |  |  |  |  |  |  |
| 2 | Vitvi14g01185\_t001 |  | \| \| |  | \| \| |  |  |  |  |  |  |  |  |
| 2 | Vitvi14g04430\_t001 |  | \| \| |  | \| \| |  |  |  |  |  |  |  |  |
| 2 | Vitvi14g01186\_t001 |  | \| \| |  | Vitvi17g00313\_t001 |  |  |  |  |  |  |  |  |
| 2 | Vitvi14g01187\_t001 |  | Vitvi01g00295\_t001 |  | \| \| |  |  |  |  |  |  |  |  |
| 2 | Vitvi14g01188\_t001 |  | Vitvi01g01938\_t001 |  | Vitvi17g04085\_t001 |  |  |  |  |  |  |  |  |
| 2 | Vitvi14g02876\_t001 |  | \| \| |  | \| \| |  |  |  |  |  |  |  |  |
| 2 | Vitvi14g04431\_t001 |  | \| \| |  | \| \| |  |  |  |  |  |  |  |  |
| 2 | Vitvi14g04432\_t001 |  | \| \| |  | \| \| |  |  |  |  |  |  |  |  |
| 2 | Vitvi14g04433\_t001 |  | \| \| |  | \| \| |  |  |  |  |  |  |  |  |
| 2 | Vitvi14g01193\_t001 |  | Vitvi01g00290\_t001 |  | Vitvi17g00325\_t001 |  |  |  |  |  |  |  |  |
| 1 | Vitvi14g01194\_t001 |  | \| \| |  |  |  |  |  |  |  |  |  |  |
| 1 | Vitvi14g01195\_t001 |  | \| \| |  |  |  |  |  |  |  |  |  |  |
| 1 | Vitvi14g04434\_t001 |  | \| \| |  |  |  |  |  |  |  |  |  |  |
| 1 | Vitvi14g01198\_t001 |  | Vitvi01g00284\_t001 |  |  |  |  |  |  |  |  |  |  |
| 1 | Vitvi14g01201\_t001 |  | \| \| |  |  |  |  |  |  |  |  |  |  |
| 1 | Vitvi14g04435\_t001 |  | \| \| |  |  |  |  |  |  |  |  |  |  |
| 1 | Vitvi14g01208\_t001 |  | Vitvi01g00278\_t001 |  |  |  |  |  |  |  |  |  |  |
| 1 | Vitvi14g04436\_t001 |  | \| \| |  |  |  |  |  |  |  |  |  |  |
| 1 | Vitvi14g04437\_t001 |  | \| \| |  |  |  |  |  |  |  |  |  |  |
| 1 | Vitvi14g01211\_t001 |  | Vitvi01g00271\_t001 |  |  |  |  |  |  |  |  |  |  |
| 1 | Vitvi14g01212\_t001 |  | Vitvi01g00268\_t001 |  |  |  |  |  |  |  |  |  |  |
| 1 | Vitvi14g01213\_t001 |  | \| \| |  |  |  |  |  |  |  |  |  |  |
| 1 | Vitvi14g04438\_t001 |  | \| \| |  |  |  |  |  |  |  |  |  |  |
| 1 | Vitvi14g04439\_t001 |  | \| \| |  |  |  |  |  |  |  |  |  |  |
| 1 | Vitvi14g04440\_t001 |  | \| \| |  |  |  |  |  |  |  |  |  |  |
| 1 | Vitvi14g01218\_t001 |  | \| \| |  |  |  |  |  |  |  |  |  |  |
| 1 | Vitvi14g02886\_t001 |  | \| \| |  |  |  |  |  |  |  |  |  |  |
| 1 | Vitvi14g01221\_t001 |  | \| \| |  |  |  |  |  |  |  |  |  |  |
| 1 | Vitvi14g01222\_t001 |  | Vitvi01g00264\_t001 |  |  |  |  |  |  |  |  |  |  |
| 1 | Vitvi14g02887\_t001 |  | Vitvi01g00262\_t001 |  |  |  |  |  |  |  |  |  |  |
| 1 | Vitvi14g01224\_t001 |  | \| \| |  |  |  |  |  |  |  |  |  |  |
| 1 | Vitvi14g02888\_t001 |  | \| \| |  |  |  |  |  |  |  |  |  |  |
| 1 | Vitvi14g02889\_t001 |  | \| \| |  |  |  |  |  |  |  |  |  |  |
| 1 | Vitvi14g01226\_t001 |  | \| \| |  |  |  |  |  |  |  |  |  |  |
| 1 | Vitvi14g01227\_t002 |  | \| \| |  |  |  |  |  |  |  |  |  |  |
| 1 | Vitvi14g02890\_t001 |  | \| \| |  |  |  |  |  |  |  |  |  |  |
| 1 | Vitvi14g01228\_t001 |  | \| \| |  |  |  |  |  |  |  |  |  |  |
| 1 | Vitvi14g04441\_t001 |  | \| \| |  |  |  |  |  |  |  |  |  |  |
| 1 | Vitvi14g01230\_t001 |  | \| \| |  |  |  |  |  |  |  |  |  |  |
| 1 | Vitvi14g01232\_t001 |  | \| \| |  |  |  |  |  |  |  |  |  |  |
| 1 | Vitvi14g04442\_t001 |  | \| \| |  |  |  |  |  |  |  |  |  |  |
| 1 | Vitvi14g04443\_t001 |  | \| \| |  |  |  |  |  |  |  |  |  |  |
| 1 | Vitvi14g04444\_t001 |  | \| \| |  |  |  |  |  |  |  |  |  |  |
| 1 | Vitvi14g01237\_t001 |  | \| \| |  |  |  |  |  |  |  |  |  |  |
| 1 | Vitvi14g01238\_t001 |  | \| \| |  |  |  |  |  |  |  |  |  |  |
| 1 | Vitvi14g01239\_t002 |  | \| \| |  |  |  |  |  |  |  |  |  |  |
| 1 | Vitvi14g01240\_t001 |  | \| \| |  |  |  |  |  |  |  |  |  |  |
| 1 | Vitvi14g01245\_t001 |  | \| \| |  |  |  |  |  |  |  |  |  |  |
| 1 | Vitvi14g01242\_t001 |  | \| \| |  |  |  |  |  |  |  |  |  |  |
| 1 | Vitvi14g04445\_t001 |  | \| \| |  |  |  |  |  |  |  |  |  |  |
| 1 | Vitvi14g01246\_t001 |  | Vitvi01g00246\_t001 |  |  |  |  |  |  |  |  |  |  |
| 1 | Vitvi14g01247\_t001 |  | \| \| |  |  |  |  |  |  |  |  |  |  |
| 1 | Vitvi14g01248\_t001 |  | Vitvi01g00244\_t001 |  |  |  |  |  |  |  |  |  |  |
| 1 | Vitvi14g01253\_t001 |  | Vitvi01g00228\_t001 |  |  |  |  |  |  |  |  |  |  |
| 1 | Vitvi14g01254\_t001 |  | \| \| |  |  |  |  |  |  |  |  |  |  |
| 1 | Vitvi14g01255\_t001 |  | \| \| |  |  |  |  |  |  |  |  |  |  |
| 1 | Vitvi14g01256\_t001 |  | Vitvi01g00222\_t001 |  |  |  |  |  |  |  |  |  |  |
| 1 | Vitvi14g04446\_t001 |  | \| \| |  |  |  |  |  |  |  |  |  |  |
| 1 | Vitvi14g04447\_t001 |  | \| \| |  |  |  |  |  |  |  |  |  |  |
| 1 | Vitvi14g01258\_t001 |  | \| \| |  |  |  |  |  |  |  |  |  |  |
| 1 | Vitvi14g01259\_t001 |  | Vitvi01g04058\_t001 |  |  |  |  |  |  |  |  |  |  |
| 0 | Vitvi14g01260\_t001 |  |  |  |  |  |  |  |  |  |  |  |  |
| 0 | Vitvi14g02893\_t001 |  |  |  |  |  |  |  |  |  |  |  |  |
| 0 | Vitvi14g04448\_t001 |  |  |  |  |  |  |  |  |  |  |  |  |
| 0 | Vitvi14g04449\_t001 |  |  |  |  |  |  |  |  |  |  |  |  |
| 0 | Vitvi14g04450\_t001 |  |  |  |  |  |  |  |  |  |  |  |  |
| 0 | Vitvi14g01262\_t001 |  |  |  |  |  |  |  |  |  |  |  |  |
| 0 | Vitvi14g01263\_t001 |  |  |  |  |  |  |  |  |  |  |  |  |
| 0 | Vitvi14g01264\_t002 |  |  |  |  |  |  |  |  |  |  |  |  |
| 0 | Vitvi14g01265\_t001 |  |  |  |  |  |  |  |  |  |  |  |  |
| 0 | Vitvi14g02897\_t001 |  |  |  |  |  |  |  |  |  |  |  |  |
| 0 | Vitvi14g04451\_t001 |  |  |  |  |  |  |  |  |  |  |  |  |
| 0 | Vitvi14g01267\_t001 |  |  |  |  |  |  |  |  |  |  |  |  |
| 0 | Vitvi14g01268\_t001 |  |  |  |  |  |  |  |  |  |  |  |  |
| 0 | Vitvi14g02898\_t001 |  |  |  |  |  |  |  |  |  |  |  |  |
| 0 | Vitvi14g02899\_t001 |  |  |  |  |  |  |  |  |  |  |  |  |
| 0 | Vitvi14g01269\_t001 |  |  |  |  |  |  |  |  |  |  |  |  |
| 0 | Vitvi14g04452\_t001 |  |  |  |  |  |  |  |  |  |  |  |  |
| 0 | Vitvi14g01271\_t001 |  |  |  |  |  |  |  |  |  |  |  |  |
| 0 | Vitvi14g01272\_t001 |  |  |  |  |  |  |  |  |  |  |  |  |
| 0 | Vitvi14g04453\_t001 |  |  |  |  |  |  |  |  |  |  |  |  |
| 1 | Vitvi14g01274\_t001 |  | Vitvi17g00412\_t001 |  |  |  |  |  |  |  |  |  |  |
| 1 | Vitvi14g04454\_t001 |  | \| \| |  |  |  |  |  |  |  |  |  |  |
| 1 | Vitvi14g01275\_t001 |  | \| \| |  |  |  |  |  |  |  |  |  |  |
| 1 | Vitvi14g01276\_t001 |  | \| \| |  |  |  |  |  |  |  |  |  |  |
| 1 | Vitvi14g04455\_t001 |  | \| \| |  |  |  |  |  |  |  |  |  |  |
| 1 | Vitvi14g02900\_t001 |  | \| \| |  |  |  |  |  |  |  |  |  |  |
| 1 | Vitvi14g01278\_t001 |  | \| \| |  |  |  |  |  |  |  |  |  |  |
| 1 | Vitvi14g01279\_t001 |  | \| \| |  |  |  |  |  |  |  |  |  |  |
| 1 | Vitvi14g01280\_t001 |  | \| \| |  |  |  |  |  |  |  |  |  |  |
| 1 | Vitvi14g01281\_t001 |  | \| \| |  |  |  |  |  |  |  |  |  |  |
| 1 | Vitvi14g04456\_t001 |  | \| \| |  |  |  |  |  |  |  |  |  |  |
| 1 | Vitvi14g04457\_t001 |  | \| \| |  |  |  |  |  |  |  |  |  |  |
| 1 | Vitvi14g02903\_t001 |  | \| \| |  |  |  |  |  |  |  |  |  |  |
| 1 | Vitvi14g01282\_t001 |  | Vitvi17g01427\_t001 |  |  |  |  |  |  |  |  |  |  |
| 1 | Vitvi14g01283\_t001 |  | \| \| |  |  |  |  |  |  |  |  |  |  |
| 1 | Vitvi14g04458\_t001 |  | \| \| |  |  |  |  |  |  |  |  |  |  |
| 1 | Vitvi14g01286\_t001 |  | \| \| |  |  |  |  |  |  |  |  |  |  |
| 1 | Vitvi14g01287\_t002 |  | \| \| |  |  |  |  |  |  |  |  |  |  |
| 1 | Vitvi14g01288\_t001 |  | \| \| |  |  |  |  |  |  |  |  |  |  |
| 1 | Vitvi14g04459\_t001 |  | \| \| |  |  |  |  |  |  |  |  |  |  |
| 1 | Vitvi14g04460\_t001 |  | \| \| |  |  |  |  |  |  |  |  |  |  |
| 1 | Vitvi14g01289\_t001 |  | \| \| |  |  |  |  |  |  |  |  |  |  |
| 1 | Vitvi14g01290\_t001 |  | \| \| |  |  |  |  |  |  |  |  |  |  |
| 1 | Vitvi14g01291\_t001 |  | \| \| |  |  |  |  |  |  |  |  |  |  |
| 1 | Vitvi14g01292\_t001 |  | \| \| |  |  |  |  |  |  |  |  |  |  |
| 1 | Vitvi14g04461\_t001 |  | \| \| |  |  |  |  |  |  |  |  |  |  |
| 2 | Vitvi14g01294\_t001 |  | \| \| |  | Vitvi01g01351\_t001 |  |  |  |  |  |  |  |  |
| 2 | Vitvi14g01295\_t001 |  | \| \| |  | Vitvi01g01352\_t001 |  |  |  |  |  |  |  |  |
| 2 | Vitvi14g01296\_t001 |  | \| \| |  | \| \| |  |  |  |  |  |  |  |  |
| 2 | Vitvi14g04462\_t001 |  | \| \| |  | \| \| |  |  |  |  |  |  |  |  |
| 2 | Vitvi14g04463\_t001 |  | \| \| |  | \| \| |  |  |  |  |  |  |  |  |
| 2 | Vitvi14g02908\_t001 |  | \| \| |  | \| \| |  |  |  |  |  |  |  |  |
| 2 | Vitvi14g02909\_t001 |  | Vitvi17g01428\_t001 |  | \| \| |  |  |  |  |  |  |  |  |
| 2 | Vitvi14g04464\_t001 |  | \| \| |  | \| \| |  |  |  |  |  |  |  |  |
| 2 | Vitvi14g04465\_t001 |  | \| \| |  | \| \| |  |  |  |  |  |  |  |  |
| 2 | Vitvi14g02910\_t001 |  | \| \| |  | \| \| |  |  |  |  |  |  |  |  |
| 2 | Vitvi14g02911\_t001 |  | Vitvi17g01429\_t001 |  | \| \| |  |  |  |  |  |  |  |  |
| 2 | Vitvi14g01301\_t002 |  | \| \| |  | \| \| |  |  |  |  |  |  |  |  |
| 2 | Vitvi14g01302\_t001 |  | Vitvi17g00434\_t001 |  | Vitvi01g01342\_t001 |  |  |  |  |  |  |  |  |
| 2 | Vitvi14g01303\_t001 |  | Vitvi17g00435\_t001 |  | \| \| |  |  |  |  |  |  |  |  |
| 2 | Vitvi14g04466\_t001 |  | \| \| |  | \| \| |  |  |  |  |  |  |  |  |
| 2 | Vitvi14g02912\_t001 |  | \| \| |  | \| \| |  |  |  |  |  |  |  |  |
| 2 | Vitvi14g01305\_t001 |  | \| \| |  | \| \| |  |  |  |  |  |  |  |  |
| 2 | Vitvi14g01306\_t003 |  | \| \| |  | Vitvi01g01317\_t001 |  |  |  |  |  |  |  |  |
| 2 | Vitvi14g04467\_t001 |  | \| \| |  | \| \| |  |  |  |  |  |  |  |  |
| 2 | Vitvi14g01307\_t001 |  | \| \| |  | \| \| |  |  |  |  |  |  |  |  |
| 2 | Vitvi14g01308\_t001 |  | \| \| |  | \| \| |  |  |  |  |  |  |  |  |
| 2 | Vitvi14g01309\_t001 |  | \| \| |  | \| \| |  |  |  |  |  |  |  |  |
| 2 | Vitvi14g01310\_t001 |  | \| \| |  | \| \| |  |  |  |  |  |  |  |  |
| 2 | Vitvi14g01311\_t001 |  | \| \| |  | Vitvi01g01312\_t001 |  |  |  |  |  |  |  |  |
| 2 | Vitvi14g04468\_t001 |  | \| \| |  | \| \| |  |  |  |  |  |  |  |  |
| 2 | Vitvi14g01312\_t001 |  | \| \| |  | Vitvi01g01307\_t001 |  |  |  |  |  |  |  |  |
| 2 | Vitvi14g01313\_t001 |  | \| \| |  | \| \| |  |  |  |  |  |  |  |  |
| 2 | Vitvi14g01314\_t001 |  | \| \| |  | \| \| |  |  |  |  |  |  |  |  |
| 2 | Vitvi14g01315\_t001 |  | \| \| |  | \| \| |  |  |  |  |  |  |  |  |
| 2 | Vitvi14g01316\_t001 |  | \| \| |  | Vitvi01g01304\_t001 |  |  |  |  |  |  |  |  |
| 2 | Vitvi14g01317\_t001 |  | \| \| |  | \| \| |  |  |  |  |  |  |  |  |
| 2 | Vitvi14g01318\_t001 |  | \| \| |  | \| \| |  |  |  |  |  |  |  |  |
| 2 | Vitvi14g01319\_t001 |  | \| \| |  | \| \| |  |  |  |  |  |  |  |  |
| 2 | Vitvi14g01321\_t001 |  | Vitvi17g00441\_t001 |  | Vitvi01g01299\_t001 |  |  |  |  |  |  |  |  |
| 2 | Vitvi14g01322\_t001 |  | \| \| |  | \| \| |  |  |  |  |  |  |  |  |
| 2 | Vitvi14g04469\_t001 |  | \| \| |  | \| \| |  |  |  |  |  |  |  |  |
| 2 | Vitvi14g01323\_t001 |  | \| \| |  | \| \| |  |  |  |  |  |  |  |  |
| 2 | Vitvi14g01325\_t001 |  | \| \| |  | \| \| |  |  |  |  |  |  |  |  |
| 2 | Vitvi14g04470\_t001 |  | \| \| |  | \| \| |  |  |  |  |  |  |  |  |
| 2 | Vitvi14g01326\_t001 |  | Vitvi17g00442\_t001 |  | Vitvi01g01287\_t001 |  |  |  |  |  |  |  |  |
| 1 | Vitvi14g01327\_t001 |  | Vitvi17g00443\_t001 |  |  |  |  |  |  |  |  |  |  |
| 1 | Vitvi14g01329\_t001 |  | \| \| |  |  |  |  |  |  |  |  |  |  |
| 1 | Vitvi14g01330\_t001 |  | \| \| |  |  |  |  |  |  |  |  |  |  |
| 1 | Vitvi14g01332\_t001 |  | Vitvi17g00457\_t001 |  |  |  |  |  |  |  |  |  |  |
| 1 | Vitvi14g01333\_t001 |  | \| \| |  |  |  |  |  |  |  |  |  |  |
| 1 | Vitvi14g04471\_t001 |  | \| \| |  |  |  |  |  |  |  |  |  |  |
| 1 | Vitvi14g02915\_t001 |  | \| \| |  |  |  |  |  |  |  |  |  |  |
| 1 | Vitvi14g01336\_t001 |  | \| \| |  |  |  |  |  |  |  |  |  |  |
| 1 | Vitvi14g01337\_t001 |  | \| \| |  |  |  |  |  |  |  |  |  |  |
| 1 | Vitvi14g01338\_t001 |  | \| \| |  |  |  |  |  |  |  |  |  |  |
| 1 | Vitvi14g04472\_t001 |  | \| \| |  |  |  |  |  |  |  |  |  |  |
| 1 | Vitvi14g01339\_t001 |  | \| \| |  |  |  |  |  |  |  |  |  |  |
| 1 | Vitvi14g01340\_t002 |  | \| \| |  |  |  |  |  |  |  |  |  |  |
| 1 | Vitvi14g01341\_t001 |  | Vitvi17g00470\_t001 |  |  |  |  |  |  |  |  |  |  |
| 1 | Vitvi14g04473\_t001 |  | \| \| |  |  |  |  |  |  |  |  |  |  |
| 1 | Vitvi14g01344\_t001 |  | \| \| |  |  |  |  |  |  |  |  |  |  |
| 1 | Vitvi14g04474\_t001 |  | \| \| |  |  |  |  |  |  |  |  |  |  |
| 1 | Vitvi14g01346\_t001 |  | \| \| |  |  |  |  |  |  |  |  |  |  |
| 1 | Vitvi14g04475\_t001 |  | \| \| |  |  |  |  |  |  |  |  |  |  |
| 1 | Vitvi14g01348\_t001 |  | \| \| |  |  |  |  |  |  |  |  |  |  |
| 1 | Vitvi14g01349\_t001 |  | \| \| |  |  |  |  |  |  |  |  |  |  |
| 1 | Vitvi14g01350\_t001 |  | Vitvi17g01440\_t001 |  |  |  |  |  |  |  |  |  |  |
| 1 | Vitvi14g01351\_t001 |  | \| \| |  |  |  |  |  |  |  |  |  |  |
| 1 | Vitvi14g01352\_t001 |  | \| \| |  |  |  |  |  |  |  |  |  |  |
| 1 | Vitvi14g01353\_t001 |  | \| \| |  |  |  |  |  |  |  |  |  |  |
| 1 | Vitvi14g01354\_t001 |  | \| \| |  |  |  |  |  |  |  |  |  |  |
| 1 | Vitvi14g04476\_t001 |  | \| \| |  |  |  |  |  |  |  |  |  |  |
| 1 | Vitvi14g01357\_t001 |  | Vitvi17g04133\_t001 |  |  |  |  |  |  |  |  |  |  |
| 0 | Vitvi14g01358\_t001 |  |  |  |  |  |  |  |  |  |  |  |  |
| 0 | Vitvi14g01360\_t001 |  |  |  |  |  |  |  |  |  |  |  |  |
| 0 | Vitvi14g01361\_t001 |  |  |  |  |  |  |  |  |  |  |  |  |
| 0 | Vitvi14g01362\_t001 |  |  |  |  |  |  |  |  |  |  |  |  |
| 0 | Vitvi14g04477\_t001 |  |  |  |  |  |  |  |  |  |  |  |  |
| 0 | Vitvi13g01753\_t001 |  |  |  |  |  |  |  |  |  |  |  |  |
| 0 | Vitvi14g04478\_t001 |  |  |  |  |  |  |  |  |  |  |  |  |
| 0 | Vitvi13g02515\_t001 |  |  |  |  |  |  |  |  |  |  |  |  |
| 2 | Vitvi13g02516\_t001 |  | Vitvi08g02381\_t001 |  | Vitvi06g04056\_t001 |  |  |  |  |  |  |  |  |
| 2 | Vitvi13g02518\_t001 |  | \| \| |  | \| \| |  |  |  |  |  |  |  |  |
| 2 | Vitvi13g01754\_t001 |  | \| \| |  | \| \| |  |  |  |  |  |  |  |  |
| 2 | Vitvi13g01755\_t001 |  | Vitvi08g01864\_t001 |  | Vitvi06g00189\_t003 |  |  |  |  |  |  |  |  |
| 2 | Vitvi14g04479\_t001 |  | \| \| |  | \| \| |  |  |  |  |  |  |  |  |
| 2 | Vitvi13g01756\_t001 |  | \| \| |  | Vitvi06g00186\_t001 |  |  |  |  |  |  |  |  |
| 2 | Vitvi13g01757\_t003 |  | \| \| |  | \| \| |  |  |  |  |  |  |  |  |
| 2 | Vitvi13g01758\_t001 |  | \| \| |  | \| \| |  |  |  |  |  |  |  |  |
| 2 | Vitvi13g01759\_t001 |  | \| \| |  | \| \| |  |  |  |  |  |  |  |  |
| 2 | Vitvi13g01760\_t001 |  | \| \| |  | Vitvi06g00182\_t001 |  |  |  |  |  |  |  |  |
| 2 | Vitvi13g01761\_t001 |  | \| \| |  | \| \| |  |  |  |  |  |  |  |  |
| 2 | Vitvi13g01762\_t002 |  | \| \| |  | \| \| |  |  |  |  |  |  |  |  |
| 2 | Vitvi13g01764\_t001 |  | Vitvi08g01852\_t001 |  | Vitvi06g00181\_t001 |  |  |  |  |  |  |  |  |
| 2 | Vitvi13g01765\_t001 |  | Vitvi08g02377\_t001 |  | \| \| |  |  |  |  |  |  |  |  |
| 2 | Vitvi14g04480\_t001 |  | \| \| |  | \| \| |  |  |  |  |  |  |  |  |
| 2 | Vitvi13g02519\_t001 |  | \| \| |  | \| \| |  |  |  |  |  |  |  |  |
| 2 | Vitvi13g01766\_t001 |  | Vitvi08g02372\_t001 |  | \| \| |  |  |  |  |  |  |  |  |
| 2 | Vitvi13g02520\_t001 |  | \| \| |  | \| \| |  |  |  |  |  |  |  |  |
| 2 | Vitvi13g01767\_t001 |  | \| \| |  | \| \| |  |  |  |  |  |  |  |  |
| 2 | Vitvi14g04481\_t001 |  | \| \| |  | Vitvi06g04052\_t001 |  |  |  |  |  |  |  |  |
| 2 | Vitvi13g01769\_t002 |  | \| \| |  | \| \| |  |  |  |  |  |  |  |  |
| 2 | Vitvi13g01770\_t001 |  | \| \| |  | \| \| |  |  |  |  |  |  |  |  |
| 2 | Vitvi13g01771\_t001 |  | Vitvi08g01844\_t001 |  | \| \| |  |  |  |  |  |  |  |  |
| 1 | Vitvi13g01772\_t001 |  |  |  | \| \| |  |  |  |  |  |  |  |  |
| 1 | Vitvi13g01773\_t001 |  |  |  | Vitvi06g00162\_t001 |  |  |  |  |  |  |  |  |
| 1 | Vitvi13g01774\_t001 |  |  |  | \| \| |  |  |  |  |  |  |  |  |
| 1 | Vitvi13g01775\_t001 |  |  |  | \| \| |  |  |  |  |  |  |  |  |
| 1 | Vitvi14g04482\_t001 |  |  |  | \| \| |  |  |  |  |  |  |  |  |
| 1 | Vitvi13g01777\_t001 |  |  |  | \| \| |  |  |  |  |  |  |  |  |
| 1 | Vitvi13g01778\_t001 |  |  |  | \| \| |  |  |  |  |  |  |  |  |
| 1 | Vitvi13g02522\_t002 |  |  |  | \| \| |  |  |  |  |  |  |  |  |
| 1 | Vitvi14g04483\_t001 |  |  |  | \| \| |  |  |  |  |  |  |  |  |
| 1 | Vitvi14g04484\_t001 |  |  |  | \| \| |  |  |  |  |  |  |  |  |
| 1 | Vitvi14g04485\_t001 |  |  |  | \| \| |  |  |  |  |  |  |  |  |
| 1 | Vitvi14g04486\_t001 |  |  |  | \| \| |  |  |  |  |  |  |  |  |
| 1 | Vitvi14g04487\_t001 |  |  |  | \| \| |  |  |  |  |  |  |  |  |
| 1 | Vitvi13g01779\_t001 |  |  |  | \| \| |  |  |  |  |  |  |  |  |
| 1 | Vitvi14g04488\_t001 |  |  |  | \| \| |  |  |  |  |  |  |  |  |
| 1 | Vitvi14g04489\_t001 |  |  |  | Vitvi06g00155\_t001 |  |  |  |  |  |  |  |  |
| 1 | Vitvi14g04490\_t001 |  |  |  | \| \| |  |  |  |  |  |  |  |  |
| 1 | Vitvi13g01782\_t001 |  |  |  | \| \| |  |  |  |  |  |  |  |  |
| 1 | Vitvi13g01783\_t001 |  |  |  | \| \| |  |  |  |  |  |  |  |  |
| 1 | Vitvi14g04491\_t001 |  |  |  | Vitvi06g00143\_t001 |  |  |  |  |  |  |  |  |
| 1 | Vitvi14g04492\_t001 |  |  |  | Vitvi06g00141\_t001 |  |  |  |  |  |  |  |  |
| 0 | Vitvi14g04493\_t001 |  |  |  |  |  |  |  |  |  |  |  |  |
| 0 | Vitvi13g02526\_t001 |  |  |  |  |  |  |  |  |  |  |  |  |
| 0 | Vitvi13g02530\_t001 |  |  |  |  |  |  |  |  |  |  |  |  |
| 0 | Vitvi14g04494\_t001 |  |  |  |  |  |  |  |  |  |  |  |  |
| 0 | Vitvi14g04495\_t001 |  |  |  |  |  |  |  |  |  |  |  |  |
| 0 | Vitvi13g02538\_t001 |  |  |  |  |  |  |  |  |  |  |  |  |
| 0 | Vitvi13g02541\_t001 |  |  |  |  |  |  |  |  |  |  |  |  |
| 0 | Vitvi13g01792\_t001 |  |  |  |  |  |  |  |  |  |  |  |  |
| 0 | Vitvi14g04496\_t001 |  |  |  |  |  |  |  |  |  |  |  |  |
| 0 | Vitvi14g04497\_t001 |  |  |  |  |  |  |  |  |  |  |  |  |
| 0 | Vitvi14g04498\_t001 |  |  |  |  |  |  |  |  |  |  |  |  |
| 1 | Vitvi14g04499\_t001 |  | Vitvi13g04771\_t001 |  |  |  |  |  |  |  |  |  |  |
| 1 | Vitvi13g01794\_t001 |  | \| \| |  |  |  |  |  |  |  |  |  |  |
| 1 | Vitvi14g04500\_t001 |  | \| \| |  |  |  |  |  |  |  |  |  |  |
| 1 | Vitvi14g04501\_t001 |  | \| \| |  |  |  |  |  |  |  |  |  |  |
| 1 | Vitvi13g01796\_t001 |  | Vitvi13g01813\_t001 |  |  |  |  |  |  |  |  |  |  |
| 1 | Vitvi14g04502\_t001 |  | \| \| |  |  |  |  |  |  |  |  |  |  |
| 1 | Vitvi13g02544\_t001 |  | \| \| |  |  |  |  |  |  |  |  |  |  |
| 1 | Vitvi14g04503\_t001 |  | Vitvi13g01812\_t001 |  |  |  |  |  |  |  |  |  |  |
| 1 | Vitvi14g04504\_t001 |  | Vitvi13g01811\_t001 |  |  |  |  |  |  |  |  |  |  |
| 1 | Vitvi14g04505\_t001 |  | Vitvi13g04785\_t001 |  |  |  |  |  |  |  |  |  |  |
| 1 | Vitvi13g01798\_t001 |  | Vitvi13g04788\_t001 |  |  |  |  |  |  |  |  |  |  |
| 1 | Vitvi13g01799\_t001 |  | \| \| |  |  |  |  |  |  |  |  |  |  |
| 1 | Vitvi14g04506\_t001 |  | \| \| |  |  |  |  |  |  |  |  |  |  |
| 1 | Vitvi14g04507\_t001 |  | \| \| |  |  |  |  |  |  |  |  |  |  |
| 1 | Vitvi14g04508\_t001 |  | \| \| |  |  |  |  |  |  |  |  |  |  |
| 1 | Vitvi13g01800\_t001 |  | Vitvi13g01805\_t001 |  |  |  |  |  |  |  |  |  |  |
| 0 | Vitvi14g04509\_t001 |  |  |  |  |  |  |  |  |  |  |  |  |
| 0 | Vitvi14g04510\_t001 |  |  |  |  |  |  |  |  |  |  |  |  |
| 0 | Vitvi14g04511\_t001 |  |  |  |  |  |  |  |  |  |  |  |  |
| 0 | Vitvi13g01802\_t001 |  |  |  |  |  |  |  |  |  |  |  |  |
| 0 | Vitvi14g04512\_t001 |  |  |  |  |  |  |  |  |  |  |  |  |
| 0 | Vitvi14g04513\_t001 |  |  |  |  |  |  |  |  |  |  |  |  |
| 0 | Vitvi14g04514\_t001 |  |  |  |  |  |  |  |  |  |  |  |  |
| 0 | Vitvi14g01368\_t001 |  |  |  |  |  |  |  |  |  |  |  |  |
| 0 | Vitvi14g01367\_t001 |  |  |  |  |  |  |  |  |  |  |  |  |
| 0 | Vitvi14g01365\_t001 |  |  |  |  |  |  |  |  |  |  |  |  |
| 0 | Vitvi14g02920\_t001 |  |  |  |  |  |  |  |  |  |  |  |  |
| 0 | Vitvi14g04515\_t001 |  |  |  |  |  |  |  |  |  |  |  |  |
| 0 | Vitvi14g01373\_t001 |  |  |  |  |  |  |  |  |  |  |  |  |
| 0 | Vitvi14g01375\_t001 |  |  |  |  |  |  |  |  |  |  |  |  |
| 0 | Vitvi14g04516\_t001 |  |  |  |  |  |  |  |  |  |  |  |  |
| 0 | Vitvi14g02923\_t001 |  |  |  |  |  |  |  |  |  |  |  |  |
| 0 | Vitvi14g02924\_t001 |  |  |  |  |  |  |  |  |  |  |  |  |
| 0 | Vitvi14g01378\_t001 |  |  |  |  |  |  |  |  |  |  |  |  |
| 0 | Vitvi14g04517\_t001 |  |  |  |  |  |  |  |  |  |  |  |  |
| 0 | Vitvi14g02926\_t001 |  |  |  |  |  |  |  |  |  |  |  |  |
| 0 | Vitvi14g02927\_t001 |  |  |  |  |  |  |  |  |  |  |  |  |
| 0 | Vitvi14g04518\_t001 |  |  |  |  |  |  |  |  |  |  |  |  |
| 0 | Vitvi14g01379\_t001 |  |  |  |  |  |  |  |  |  |  |  |  |
| 0 | Vitvi14g02928\_t001 |  |  |  |  |  |  |  |  |  |  |  |  |
| 0 | Vitvi14g01380\_t001 |  |  |  |  |  |  |  |  |  |  |  |  |
| 0 | Vitvi14g04519\_t001 |  |  |  |  |  |  |  |  |  |  |  |  |
| 0 | Vitvi14g01381\_t001 |  |  |  |  |  |  |  |  |  |  |  |  |
| 0 | Vitvi14g02929\_t001 |  |  |  |  |  |  |  |  |  |  |  |  |
| 0 | Vitvi14g02930\_t001 |  |  |  |  |  |  |  |  |  |  |  |  |
| 0 | Vitvi14g04520\_t001 |  |  |  |  |  |  |  |  |  |  |  |  |
| 0 | Vitvi14g04521\_t001 |  |  |  |  |  |  |  |  |  |  |  |  |
| 0 | Vitvi14g04522\_t001 |  |  |  |  |  |  |  |  |  |  |  |  |
| 0 | Vitvi14g04523\_t001 |  |  |  |  |  |  |  |  |  |  |  |  |
| 0 | Vitvi14g02932\_t001 |  |  |  |  |  |  |  |  |  |  |  |  |
| 0 | Vitvi14g04524\_t001 |  |  |  |  |  |  |  |  |  |  |  |  |
| 0 | Vitvi14g04525\_t001 |  |  |  |  |  |  |  |  |  |  |  |  |
| 0 | Vitvi14g02934\_t001 |  |  |  |  |  |  |  |  |  |  |  |  |
| 0 | Vitvi14g02935\_t001 |  |  |  |  |  |  |  |  |  |  |  |  |
| 0 | Vitvi14g01385\_t001 |  |  |  |  |  |  |  |  |  |  |  |  |
| 0 | Vitvi14g04526\_t001 |  |  |  |  |  |  |  |  |  |  |  |  |
| 0 | Vitvi14g01387\_t001 |  |  |  |  |  |  |  |  |  |  |  |  |
| 0 | Vitvi14g01389\_t001 |  |  |  |  |  |  |  |  |  |  |  |  |
| 0 | Vitvi14g01390\_t001 |  |  |  |  |  |  |  |  |  |  |  |  |
| 0 | Vitvi14g04527\_t001 |  |  |  |  |  |  |  |  |  |  |  |  |
| 0 | Vitvi14g01391\_t002 |  |  |  |  |  |  |  |  |  |  |  |  |
| 0 | Vitvi14g02938\_t001 |  |  |  |  |  |  |  |  |  |  |  |  |
| 0 | Vitvi14g01392\_t001 |  |  |  |  |  |  |  |  |  |  |  |  |
| 0 | Vitvi14g01394\_t001 |  |  |  |  |  |  |  |  |  |  |  |  |
| 0 | Vitvi14g04528\_t001 |  |  |  |  |  |  |  |  |  |  |  |  |
| 0 | Vitvi14g01395\_t001 |  |  |  |  |  |  |  |  |  |  |  |  |
| 0 | Vitvi14g04529\_t001 |  |  |  |  |  |  |  |  |  |  |  |  |
| 0 | Vitvi14g01398\_t001 |  |  |  |  |  |  |  |  |  |  |  |  |
| 0 | Vitvi14g04530\_t001 |  |  |  |  |  |  |  |  |  |  |  |  |
| 0 | Vitvi14g01400\_t001 |  |  |  |  |  |  |  |  |  |  |  |  |
| 0 | Vitvi14g01401\_t001 |  |  |  |  |  |  |  |  |  |  |  |  |
| 0 | Vitvi14g01402\_t001 |  |  |  |  |  |  |  |  |  |  |  |  |
| 0 | Vitvi14g01403\_t001 |  |  |  |  |  |  |  |  |  |  |  |  |
| 0 | Vitvi14g01404\_t001 |  |  |  |  |  |  |  |  |  |  |  |  |
| 0 | Vitvi14g01405\_t002 |  |  |  |  |  |  |  |  |  |  |  |  |
| 0 | Vitvi14g01406\_t002 |  |  |  |  |  |  |  |  |  |  |  |  |
| 0 | Vitvi14g01407\_t003 |  |  |  |  |  |  |  |  |  |  |  |  |
| 0 | Vitvi14g01409\_t001 |  |  |  |  |  |  |  |  |  |  |  |  |
| 0 | Vitvi14g02941\_t001 |  |  |  |  |  |  |  |  |  |  |  |  |
| 0 | Vitvi14g02942\_t001 |  |  |  |  |  |  |  |  |  |  |  |  |
| 0 | Vitvi14g02945\_t001 |  |  |  |  |  |  |  |  |  |  |  |  |
| 0 | Vitvi14g04531\_t001 |  |  |  |  |  |  |  |  |  |  |  |  |
| 0 | Vitvi14g02943\_t001 |  |  |  |  |  |  |  |  |  |  |  |  |
| 0 | Vitvi14g02944\_t001 |  |  |  |  |  |  |  |  |  |  |  |  |
| 0 | Vitvi14g02946\_t001 |  |  |  |  |  |  |  |  |  |  |  |  |
| 0 | Vitvi14g04532\_t001 |  |  |  |  |  |  |  |  |  |  |  |  |
| 0 | Vitvi14g01413\_t001 |  |  |  |  |  |  |  |  |  |  |  |  |
| 0 | Vitvi14g04533\_t001 |  |  |  |  |  |  |  |  |  |  |  |  |
| 0 | Vitvi14g01414\_t001 |  |  |  |  |  |  |  |  |  |  |  |  |
| 0 | Vitvi14g01415\_t001 |  |  |  |  |  |  |  |  |  |  |  |  |
| 0 | Vitvi14g01416\_t001 |  |  |  |  |  |  |  |  |  |  |  |  |
| 0 | Vitvi14g04534\_t001 |  |  |  |  |  |  |  |  |  |  |  |  |
| 0 | Vitvi14g01417\_t001 |  |  |  |  |  |  |  |  |  |  |  |  |
| 0 | Vitvi14g01418\_t001 |  |  |  |  |  |  |  |  |  |  |  |  |
| 0 | Vitvi14g01419\_t001 |  |  |  |  |  |  |  |  |  |  |  |  |
| 0 | Vitvi14g01420\_t001 |  |  |  |  |  |  |  |  |  |  |  |  |
| 0 | Vitvi14g01422\_t001 |  |  |  |  |  |  |  |  |  |  |  |  |
| 0 | Vitvi14g01423\_t001 |  |  |  |  |  |  |  |  |  |  |  |  |
| 1 | Vitvi14g01424\_t001 |  | Vitvi01g01807\_t001 |  |  |  |  |  |  |  |  |  |  |
| 1 | Vitvi14g01425\_t001 |  | \| \| |  |  |  |  |  |  |  |  |  |  |
| 1 | Vitvi14g01426\_t001 |  | \| \| |  |  |  |  |  |  |  |  |  |  |
| 1 | Vitvi14g01427\_t002 |  | \| \| |  |  |  |  |  |  |  |  |  |  |
| 2 | Vitvi14g01428\_t001 |  | \| \| |  | Vitvi17g00002\_t001 |  |  |  |  |  |  |  |  |
| 2 | Vitvi14g01429\_t001 |  | Vitvi01g01798\_t001 |  | \| \| |  |  |  |  |  |  |  |  |
| 2 | Vitvi14g02948\_t001 |  | \| \| |  | \| \| |  |  |  |  |  |  |  |  |
| 2 | Vitvi14g02949\_t001 |  | Vitvi01g02300\_t001 |  | \| \| |  |  |  |  |  |  |  |  |
| 2 | Vitvi14g01431\_t001 |  | \| \| |  | \| \| |  |  |  |  |  |  |  |  |
| 2 | Vitvi14g01432\_t001 |  | \| \| |  | \| \| |  |  |  |  |  |  |  |  |
| 2 | Vitvi14g01433\_t001 |  | \| \| |  | \| \| |  |  |  |  |  |  |  |  |
| 2 | Vitvi14g04535\_t001 |  | \| \| |  | \| \| |  |  |  |  |  |  |  |  |
| 2 | Vitvi14g01434\_t001 |  | \| \| |  | \| \| |  |  |  |  |  |  |  |  |
| 2 | Vitvi14g01435\_t001 |  | \| \| |  | \| \| |  |  |  |  |  |  |  |  |
| 2 | Vitvi14g01436\_t001 |  | \| \| |  | \| \| |  |  |  |  |  |  |  |  |
| 2 | Vitvi14g01437\_t001 |  | \| \| |  | \| \| |  |  |  |  |  |  |  |  |
| 2 | Vitvi14g04536\_t001 |  | \| \| |  | \| \| |  |  |  |  |  |  |  |  |
| 2 | Vitvi14g01439\_t001 |  | \| \| |  | \| \| |  |  |  |  |  |  |  |  |
| 2 | Vitvi14g01440\_t001 |  | \| \| |  | \| \| |  |  |  |  |  |  |  |  |
| 2 | Vitvi14g04537\_t001 |  | \| \| |  | \| \| |  |  |  |  |  |  |  |  |
| 2 | Vitvi14g01441\_t001 |  | \| \| |  | \| \| |  |  |  |  |  |  |  |  |
| 2 | Vitvi14g04538\_t001 |  | \| \| |  | \| \| |  |  |  |  |  |  |  |  |
| 2 | Vitvi14g01442\_t001 |  | Vitvi01g01793\_t002 |  | \| \| |  |  |  |  |  |  |  |  |
| 2 | Vitvi14g01444\_t001 |  | Vitvi01g01792\_t001 |  | \| \| |  |  |  |  |  |  |  |  |
| 2 | Vitvi14g01445\_t001 |  | \| \| |  | Vitvi17g00012\_t001 |  |  |  |  |  |  |  |  |
| 2 | Vitvi14g01446\_t001 |  | \| \| |  | \| \| |  |  |  |  |  |  |  |  |
| 2 | Vitvi14g01447\_t001 |  | \| \| |  | \| \| |  |  |  |  |  |  |  |  |
| 2 | Vitvi14g01449\_t003 |  | \| \| |  | \| \| |  |  |  |  |  |  |  |  |
| 2 | Vitvi14g04539\_t001 |  | \| \| |  | \| \| |  |  |  |  |  |  |  |  |
| 2 | Vitvi14g01450\_t001 |  | \| \| |  | \| \| |  |  |  |  |  |  |  |  |
| 2 | Vitvi14g01451\_t001 |  | \| \| |  | \| \| |  |  |  |  |  |  |  |  |
| 2 | Vitvi14g04540\_t001 |  | \| \| |  | \| \| |  |  |  |  |  |  |  |  |
| 2 | Vitvi14g02950\_t001 |  | \| \| |  | \| \| |  |  |  |  |  |  |  |  |
| 2 | Vitvi14g01452\_t001 |  | \| \| |  | \| \| |  |  |  |  |  |  |  |  |
| 2 | Vitvi14g01454\_t001 |  | \| \| |  | \| \| |  |  |  |  |  |  |  |  |
| 2 | Vitvi14g01455\_t003 |  | \| \| |  | \| \| |  |  |  |  |  |  |  |  |
| 2 | Vitvi14g04541\_t001 |  | \| \| |  | \| \| |  |  |  |  |  |  |  |  |
| 2 | Vitvi14g04542\_t001 |  | \| \| |  | \| \| |  |  |  |  |  |  |  |  |
| 2 | Vitvi14g01456\_t001 |  | \| \| |  | \| \| |  |  |  |  |  |  |  |  |
| 2 | Vitvi14g01457\_t001 |  | Vitvi01g01776\_t001 |  | \| \| |  |  |  |  |  |  |  |  |
| 2 | Vitvi14g04543\_t001 |  | \| \| |  | \| \| |  |  |  |  |  |  |  |  |
| 2 | Vitvi14g04544\_t001 |  | \| \| |  | \| \| |  |  |  |  |  |  |  |  |
| 2 | Vitvi14g04545\_t001 |  | \| \| |  | \| \| |  |  |  |  |  |  |  |  |
| 2 | Vitvi14g01458\_t001 |  | Vitvi01g01826\_t001 |  | Vitvi17g00025\_t001 |  |  |  |  |  |  |  |  |
| 2 | Vitvi14g01459\_t001 |  | \| \| |  | \| \| |  |  |  |  |  |  |  |  |
| 2 | Vitvi14g01460\_t001 |  | Vitvi01g01770\_t001 |  | \| \| |  |  |  |  |  |  |  |  |
| 2 | Vitvi14g01462\_t001 |  | Vitvi01g01765\_t001 |  | \| \| |  |  |  |  |  |  |  |  |
| 2 | Vitvi14g04546\_t001 |  | \| \| |  | \| \| |  |  |  |  |  |  |  |  |
| 2 | Vitvi14g01463\_t001 |  | Vitvi01g01763\_t001 |  | \| \| |  |  |  |  |  |  |  |  |
| 2 | Vitvi14g01465\_t002 |  | \| \| |  | Vitvi17g00031\_t001 |  |  |  |  |  |  |  |  |
| 2 | Vitvi14g01466\_t001 |  | \| \| |  | \| \| |  |  |  |  |  |  |  |  |
| 2 | Vitvi14g01467\_t001 |  | \| \| |  | \| \| |  |  |  |  |  |  |  |  |
| 2 | Vitvi14g01468\_t001 |  | \| \| |  | \| \| |  |  |  |  |  |  |  |  |
| 2 | Vitvi14g01469\_t001 |  | Vitvi01g02296\_t001 |  | \| \| |  |  |  |  |  |  |  |  |
| 2 | Vitvi14g01470\_t001 |  | \| \| |  | \| \| |  |  |  |  |  |  |  |  |
| 2 | Vitvi14g01471\_t001 |  | \| \| |  | \| \| |  |  |  |  |  |  |  |  |
| 2 | Vitvi14g01472\_t001 |  | Vitvi01g01758\_t001 |  | \| \| |  |  |  |  |  |  |  |  |
| 2 | Vitvi14g04547\_t001 |  | \| \| |  | \| \| |  |  |  |  |  |  |  |  |
| 2 | Vitvi14g01473\_t001 |  | Vitvi01g01757\_t001 |  | Vitvi17g00037\_t001 |  |  |  |  |  |  |  |  |
| 2 | Vitvi14g01474\_t001 |  | \| \| |  | \| \| |  |  |  |  |  |  |  |  |
| 2 | Vitvi14g01475\_t001 |  | \| \| |  | Vitvi17g00038\_t001 |  |  |  |  |  |  |  |  |
| 2 | Vitvi14g04548\_t001 |  | \| \| |  | \| \| |  |  |  |  |  |  |  |  |
| 2 | Vitvi14g04549\_t001 |  | \| \| |  | \| \| |  |  |  |  |  |  |  |  |
| 2 | Vitvi14g04550\_t001 |  | \| \| |  | \| \| |  |  |  |  |  |  |  |  |
| 2 | Vitvi14g01476\_t001 |  | \| \| |  | \| \| |  |  |  |  |  |  |  |  |
| 2 | Vitvi14g04551\_t001 |  | \| \| |  | \| \| |  |  |  |  |  |  |  |  |
| 2 | Vitvi14g01477\_t001 |  | \| \| |  | \| \| |  |  |  |  |  |  |  |  |
| 2 | Vitvi14g02951\_t001 |  | \| \| |  | \| \| |  |  |  |  |  |  |  |  |
| 2 | Vitvi14g01478\_t002 |  | \| \| |  | Vitvi17g00044\_t001 |  |  |  |  |  |  |  |  |
| 2 | Vitvi14g04552\_t001 |  | \| \| |  | \| \| |  |  |  |  |  |  |  |  |
| 2 | Vitvi14g01479\_t001 |  | \| \| |  | Vitvi17g04009\_t001 |  |  |  |  |  |  |  |  |
| 2 | Vitvi14g01480\_t001 |  | Vitvi01g01743\_t001 |  | \| \| |  |  |  |  |  |  |  |  |
| 2 | Vitvi14g01481\_t001 |  | \| \| |  | Vitvi17g00051\_t001 |  |  |  |  |  |  |  |  |
| 2 | Vitvi14g02954\_t001 |  | Vitvi01g02291\_t001 |  | \| \| |  |  |  |  |  |  |  |  |
| 2 | Vitvi14g01482\_t001 |  | Vitvi01g01738\_t001 |  | \| \| |  |  |  |  |  |  |  |  |
| 2 | Vitvi14g04553\_t002 |  | \| \| |  | \| \| |  |  |  |  |  |  |  |  |
| 2 | Vitvi14g01483\_t001 |  | \| \| |  | \| \| |  |  |  |  |  |  |  |  |
| 2 | Vitvi14g01485\_t001 |  | Vitvi01g01734\_t001 |  | \| \| |  |  |  |  |  |  |  |  |
| 2 | Vitvi14g04554\_t002 |  | \| \| |  | \| \| |  |  |  |  |  |  |  |  |
| 2 | Vitvi14g01487\_t001 |  | Vitvi01g01729\_t001 |  | \| \| |  |  |  |  |  |  |  |  |
| 2 | Vitvi14g01488\_t001 |  | \| \| |  | \| \| |  |  |  |  |  |  |  |  |
| 2 | Vitvi14g01489\_t001 |  | \| \| |  | Vitvi17g00053\_t001 |  |  |  |  |  |  |  |  |
| 2 | Vitvi14g01491\_t001 |  | \| \| |  | \| \| |  |  |  |  |  |  |  |  |
| 2 | Vitvi14g01493\_t001 |  | \| \| |  | \| \| |  |  |  |  |  |  |  |  |
| 2 | Vitvi14g01494\_t001 |  | \| \| |  | \| \| |  |  |  |  |  |  |  |  |
| 2 | Vitvi14g01495\_t001 |  | \| \| |  | \| \| |  |  |  |  |  |  |  |  |
| 2 | Vitvi14g02957\_t001 |  | \| \| |  | \| \| |  |  |  |  |  |  |  |  |
| 2 | Vitvi14g04555\_t001 |  | \| \| |  | \| \| |  |  |  |  |  |  |  |  |
| 2 | Vitvi14g01496\_t003 |  | \| \| |  | Vitvi17g00054\_t001 |  |  |  |  |  |  |  |  |
| 2 | Vitvi14g04556\_t001 |  | \| \| |  | \| \| |  |  |  |  |  |  |  |  |
| 2 | Vitvi14g01497\_t001 |  | \| \| |  | \| \| |  |  |  |  |  |  |  |  |
| 2 | Vitvi14g01498\_t002 |  | Vitvi01g01726\_t002 |  | \| \| |  |  |  |  |  |  |  |  |
| 2 | Vitvi14g02958\_t001 |  | \| \| |  | \| \| |  |  |  |  |  |  |  |  |
| 2 | Vitvi14g01499\_t001 |  | Vitvi01g01722\_t001 |  | Vitvi17g00066\_t001 |  |  |  |  |  |  |  |  |
| 2 | Vitvi14g04557\_t001 |  | \| \| |  | \| \| |  |  |  |  |  |  |  |  |
| 2 | Vitvi14g01500\_t001 |  | \| \| |  | \| \| |  |  |  |  |  |  |  |  |
| 2 | Vitvi14g01501\_t001 |  | \| \| |  | Vitvi17g00067\_t001 |  |  |  |  |  |  |  |  |
| 2 | Vitvi14g01502\_t001 |  | \| \| |  | \| \| |  |  |  |  |  |  |  |  |
| 2 | Vitvi14g01503\_t001 |  | \| \| |  | Vitvi17g00068\_t001 |  |  |  |  |  |  |  |  |
| 2 | Vitvi14g04558\_t001 |  | \| \| |  | \| \| |  |  |  |  |  |  |  |  |
| 2 | Vitvi14g01504\_t001 |  | Vitvi01g01718\_t001 |  | \| \| |  |  |  |  |  |  |  |  |
| 2 | Vitvi14g01505\_t001 |  | \| \| |  | Vitvi17g00071\_t001 |  |  |  |  |  |  |  |  |
| 2 | Vitvi14g01506\_t002 |  | \| \| |  | Vitvi17g00072\_t001 |  |  |  |  |  |  |  |  |
| 2 | Vitvi14g04559\_t001 |  | \| \| |  | \| \| |  |  |  |  |  |  |  |  |
| 2 | Vitvi14g01507\_t001 |  | Vitvi01g02287\_t001 |  | \| \| |  |  |  |  |  |  |  |  |
| 2 | Vitvi14g01508\_t001 |  | \| \| |  | \| \| |  |  |  |  |  |  |  |  |
| 2 | Vitvi14g04560\_t001 |  | \| \| |  | \| \| |  |  |  |  |  |  |  |  |
| 2 | Vitvi14g01509\_t001 |  | \| \| |  | Vitvi17g01331\_t001 |  |  |  |  |  |  |  |  |
| 2 | Vitvi14g04561\_t001 |  | \| \| |  | \| \| |  |  |  |  |  |  |  |  |
| 2 | Vitvi14g01511\_t001 |  | Vitvi01g01714\_t001 |  | \| \| |  |  |  |  |  |  |  |  |
| 2 | Vitvi14g04562\_t001 |  | \| \| |  | \| \| |  |  |  |  |  |  |  |  |
| 2 | Vitvi14g01513\_t001 |  | \| \| |  | \| \| |  |  |  |  |  |  |  |  |
| 2 | Vitvi14g01514\_t001 |  | \| \| |  | Vitvi17g00080\_t001 |  |  |  |  |  |  |  |  |
| 2 | Vitvi14g01515\_t002 |  | Vitvi01g01707\_t001 |  | \| \| |  |  |  |  |  |  |  |  |
| 2 | Vitvi14g04563\_t001 |  | \| \| |  | \| \| |  |  |  |  |  |  |  |  |
| 2 | Vitvi14g04564\_t001 |  | \| \| |  | \| \| |  |  |  |  |  |  |  |  |
| 2 | Vitvi14g01516\_t001 |  | \| \| |  | \| \| |  |  |  |  |  |  |  |  |
| 2 | Vitvi14g01517\_t001 |  | Vitvi01g01704\_t001 |  | Vitvi17g00082\_t001 |  |  |  |  |  |  |  |  |
| 2 | Vitvi14g01519\_t001 |  | \| \| |  | \| \| |  |  |  |  |  |  |  |  |
| 2 | Vitvi14g02960\_t001 |  | \| \| |  | \| \| |  |  |  |  |  |  |  |  |
| 2 | Vitvi14g01520\_t001 |  | \| \| |  | \| \| |  |  |  |  |  |  |  |  |
| 2 | Vitvi14g02961\_t001 |  | Vitvi01g01684\_t001 |  | \| \| |  |  |  |  |  |  |  |  |
| 2 | Vitvi14g01521\_t001 |  | \| \| |  | \| \| |  |  |  |  |  |  |  |  |
| 2 | Vitvi14g04565\_t001 |  | \| \| |  | \| \| |  |  |  |  |  |  |  |  |
| 2 | Vitvi14g01522\_t001 |  | \| \| |  | \| \| |  |  |  |  |  |  |  |  |
| 2 | Vitvi14g01523\_t001 |  | Vitvi01g01680\_t001 |  | \| \| |  |  |  |  |  |  |  |  |
| 2 | Vitvi14g01524\_t001 |  | Vitvi01g01678\_t001 |  | \| \| |  |  |  |  |  |  |  |  |
| 2 | Vitvi14g01525\_t001 |  | Vitvi01g04450\_t001 |  | \| \| |  |  |  |  |  |  |  |  |
| 2 | Vitvi14g01526\_t001 |  | Vitvi01g01673\_t001 |  | \| \| |  |  |  |  |  |  |  |  |
| 2 | Vitvi14g02964\_t001 |  | Vitvi01g04445\_t001 |  | \| \| |  |  |  |  |  |  |  |  |
| 2 | Vitvi14g02965\_t001 |  | \| \| |  | \| \| |  |  |  |  |  |  |  |  |
| 2 | Vitvi14g04566\_t001 |  | \| \| |  | \| \| |  |  |  |  |  |  |  |  |
| 2 | Vitvi14g01527\_t001 |  | \| \| |  | \| \| |  |  |  |  |  |  |  |  |
| 2 | Vitvi14g01530\_t004 |  | \| \| |  | Vitvi17g00091\_t001 |  |  |  |  |  |  |  |  |
| 2 | Vitvi14g04567\_t001 |  | \| \| |  | \| \| |  |  |  |  |  |  |  |  |
| 2 | Vitvi14g04568\_t001 |  | \| \| |  | \| \| |  |  |  |  |  |  |  |  |
| 2 | Vitvi14g01532\_t001 |  | \| \| |  | \| \| |  |  |  |  |  |  |  |  |
| 2 | Vitvi14g01533\_t001 |  | \| \| |  | \| \| |  |  |  |  |  |  |  |  |
| 2 | Vitvi14g01534\_t001 |  | \| \| |  | Vitvi17g01346\_t001 |  |  |  |  |  |  |  |  |
| 2 | Vitvi14g01535\_t001 |  | \| \| |  | \| \| |  |  |  |  |  |  |  |  |
| 2 | Vitvi14g01537\_t001 |  | \| \| |  | Vitvi17g00104\_t001 |  |  |  |  |  |  |  |  |
| 2 | Vitvi14g01540\_t001 |  | Vitvi01g01655\_t001 |  | Vitvi17g00106\_t001 |  |  |  |  |  |  |  |  |
| 1 | Vitvi14g02971\_t001 |  |  |  | \| \| |  |  |  |  |  |  |  |  |
| 1 | Vitvi14g01542\_t001 |  |  |  | \| \| |  |  |  |  |  |  |  |  |
| 1 | Vitvi14g02972\_t001 |  |  |  | \| \| |  |  |  |  |  |  |  |  |
| 1 | Vitvi14g01543\_t001 |  |  |  | \| \| |  |  |  |  |  |  |  |  |
| 1 | Vitvi14g01544\_t001 |  |  |  | \| \| |  |  |  |  |  |  |  |  |
| 1 | Vitvi14g01545\_t001 |  |  |  | \| \| |  |  |  |  |  |  |  |  |
| 1 | Vitvi14g01546\_t001 |  |  |  | \| \| |  |  |  |  |  |  |  |  |
| 1 | Vitvi14g04569\_t001 |  |  |  | \| \| |  |  |  |  |  |  |  |  |
| 1 | Vitvi14g01548\_t001 |  |  |  | \| \| |  |  |  |  |  |  |  |  |
| 1 | Vitvi14g01549\_t001 |  |  |  | \| \| |  |  |  |  |  |  |  |  |
| 1 | Vitvi14g01550\_t001 |  |  |  | \| \| |  |  |  |  |  |  |  |  |
| 1 | Vitvi14g01551\_t001 |  |  |  | \| \| |  |  |  |  |  |  |  |  |
| 1 | Vitvi14g01552\_t001 |  |  |  | Vitvi17g00119\_t001 |  |  |  |  |  |  |  |  |
| 1 | Vitvi14g01553\_t001 |  |  |  | \| \| |  |  |  |  |  |  |  |  |
| 1 | Vitvi14g01554\_t001 |  |  |  | Vitvi17g00120\_t001 |  |  |  |  |  |  |  |  |
| 0 | Vitvi14g01555\_t002 |  |  |  |  |  |  |  |  |  |  |  |  |
| 0 | Vitvi14g04570\_t001 |  |  |  |  |  |  |  |  |  |  |  |  |
| 0 | Vitvi14g01558\_t001 |  |  |  |  |  |  |  |  |  |  |  |  |
| 0 | Vitvi14g01560\_t001 |  |  |  |  |  |  |  |  |  |  |  |  |
| 0 | Vitvi14g01561\_t001 |  |  |  |  |  |  |  |  |  |  |  |  |
| 0 | Vitvi14g04571\_t001 |  |  |  |  |  |  |  |  |  |  |  |  |
| 0 | Vitvi14g04572\_t001 |  |  |  |  |  |  |  |  |  |  |  |  |
| 0 | Vitvi14g01563\_t001 |  |  |  |  |  |  |  |  |  |  |  |  |
| 0 | Vitvi14g04573\_t001 |  |  |  |  |  |  |  |  |  |  |  |  |
| 0 | Vitvi14g04574\_t001 |  |  |  |  |  |  |  |  |  |  |  |  |
| 0 | Vitvi14g02406\_t001 |  |  |  |  |  |  |  |  |  |  |  |  |
| 0 | Vitvi14g04575\_t001 |  |  |  |  |  |  |  |  |  |  |  |  |
| 0 | Vitvi14g04576\_t001 |  |  |  |  |  |  |  |  |  |  |  |  |
| 0 | Vitvi14g04577\_t001 |  |  |  |  |  |  |  |  |  |  |  |  |
| 0 | Vitvi14g04578\_t001 |  |  |  |  |  |  |  |  |  |  |  |  |
| 0 | Vitvi14g04579\_t001 |  |  |  |  |  |  |  |  |  |  |  |  |
| 0 | Vitvi14g04580\_t001 |  |  |  |  |  |  |  |  |  |  |  |  |
| 0 | Vitvi14g04581\_t001 |  |  |  |  |  |  |  |  |  |  |  |  |
| 0 | Vitvi14g04582\_t001 |  |  |  |  |  |  |  |  |  |  |  |  |
| 0 | Vitvi14g04583\_t001 |  |  |  |  |  |  |  |  |  |  |  |  |
| 0 | Vitvi14g04584\_t001 |  |  |  |  |  |  |  |  |  |  |  |  |
| 0 | Vitvi14g04585\_t001 |  |  |  |  |  |  |  |  |  |  |  |  |
| 0 | Vitvi14g04586\_t001 |  |  |  |  |  |  |  |  |  |  |  |  |
| 1 | Vitvi14g03121\_t001 |  | Vitvi01g02254\_t001 |  |  |  |  |  |  |  |  |  |  |
| 1 | Vitvi14g02982\_t001 |  | \| \| |  |  |  |  |  |  |  |  |  |  |
| 1 | Vitvi14g02411\_t001 |  | \| \| |  |  |  |  |  |  |  |  |  |  |
| 1 | Vitvi14g02983\_t001 |  | \| \| |  |  |  |  |  |  |  |  |  |  |
| 1 | Vitvi14g04587\_t001 |  | \| \| |  |  |  |  |  |  |  |  |  |  |
| 1 | Vitvi14g04588\_t001 |  | \| \| |  |  |  |  |  |  |  |  |  |  |
| 2 | Vitvi14g02984\_t001 |  | \| \| |  | Vitvi17g00127\_t001 |  |  |  |  |  |  |  |  |
| 2 | Vitvi14g01568\_t001 |  | \| \| |  | \| \| |  |  |  |  |  |  |  |  |
| 2 | Vitvi14g01569\_t001 |  | \| \| |  | \| \| |  |  |  |  |  |  |  |  |
| 2 | Vitvi14g02985\_t001 |  | Vitvi01g01591\_t001 |  | \| \| |  |  |  |  |  |  |  |  |
| 2 | Vitvi14g01571\_t001 |  | \| \| |  | \| \| |  |  |  |  |  |  |  |  |
| 2 | Vitvi14g04589\_t001 |  | \| \| |  | \| \| |  |  |  |  |  |  |  |  |
| 2 | Vitvi14g02422\_t001 |  | \| \| |  | \| \| |  |  |  |  |  |  |  |  |
| 2 | Vitvi14g01573\_t001 |  | Vitvi01g02228\_t001 |  | \| \| |  |  |  |  |  |  |  |  |
| 2 | Vitvi14g01574\_t001 |  | \| \| |  | Vitvi17g00137\_t001 |  |  |  |  |  |  |  |  |
| 2 | Vitvi14g01575\_t001 |  | Vitvi01g01567\_t001 |  | \| \| |  |  |  |  |  |  |  |  |
| 2 | Vitvi14g04590\_t001 |  | \| \| |  | \| \| |  |  |  |  |  |  |  |  |
| 2 | Vitvi14g01576\_t001 |  | \| \| |  | Vitvi17g00139\_t001 |  |  |  |  |  |  |  |  |
| 2 | Vitvi14g04591\_t001 |  | \| \| |  | \| \| |  |  |  |  |  |  |  |  |
| 2 | Vitvi14g04592\_t001 |  | \| \| |  | \| \| |  |  |  |  |  |  |  |  |
| 2 | Vitvi14g01579\_t001 |  | \| \| |  | \| \| |  |  |  |  |  |  |  |  |
| 2 | Vitvi14g04593\_t001 |  | \| \| |  | \| \| |  |  |  |  |  |  |  |  |
| 2 | Vitvi14g02986\_t001 |  | \| \| |  | \| \| |  |  |  |  |  |  |  |  |
| 2 | Vitvi14g01581\_t001 |  | Vitvi01g01566\_t001 |  | \| \| |  |  |  |  |  |  |  |  |
| 2 | Vitvi14g01582\_t001 |  | \| \| |  | \| \| |  |  |  |  |  |  |  |  |
| 2 | Vitvi14g04594\_t001 |  | \| \| |  | \| \| |  |  |  |  |  |  |  |  |
| 2 | Vitvi14g02428\_t001 |  | \| \| |  | \| \| |  |  |  |  |  |  |  |  |
| 2 | Vitvi14g02987\_t001 |  | \| \| |  | \| \| |  |  |  |  |  |  |  |  |
| 2 | Vitvi14g01584\_t001 |  | \| \| |  | \| \| |  |  |  |  |  |  |  |  |
| 2 | Vitvi14g01585\_t001 |  | \| \| |  | \| \| |  |  |  |  |  |  |  |  |
| 2 | Vitvi14g01586\_t001 |  | \| \| |  | \| \| |  |  |  |  |  |  |  |  |
| 2 | Vitvi14g04595\_t001 |  | \| \| |  | \| \| |  |  |  |  |  |  |  |  |
| 2 | Vitvi14g01588\_t001 |  | Vitvi01g01561\_t001 |  | Vitvi17g00148\_t001 |  |  |  |  |  |  |  |  |
| 2 | Vitvi14g01589\_t001 |  | \| \| |  | \| \| |  |  |  |  |  |  |  |  |
| 2 | Vitvi14g01590\_t001 |  | \| \| |  | \| \| |  |  |  |  |  |  |  |  |
| 2 | Vitvi14g01591\_t001 |  | \| \| |  | \| \| |  |  |  |  |  |  |  |  |
| 2 | Vitvi14g04596\_t001 |  | \| \| |  | \| \| |  |  |  |  |  |  |  |  |
| 2 | Vitvi14g02988\_t001 |  | \| \| |  | \| \| |  |  |  |  |  |  |  |  |
| 2 | Vitvi14g04597\_t001 |  | \| \| |  | \| \| |  |  |  |  |  |  |  |  |
| 2 | Vitvi14g02990\_t001 |  | \| \| |  | Vitvi17g00152\_t001 |  |  |  |  |  |  |  |  |
| 2 | Vitvi14g01593\_t001 |  | \| \| |  | \| \| |  |  |  |  |  |  |  |  |
| 2 | Vitvi14g01594\_t002 |  | \| \| |  | Vitvi17g00153\_t002 |  |  |  |  |  |  |  |  |
| 1 | Vitvi14g01595\_t001 |  | Vitvi01g01558\_t001 |  |  |  |  |  |  |  |  |  |  |
| 1 | Vitvi14g01596\_t001 |  | Vitvi01g01555\_t001 |  |  |  |  |  |  |  |  |  |  |
| 1 | Vitvi14g04598\_t001 |  | \| \| |  |  |  |  |  |  |  |  |  |  |
| 1 | Vitvi14g01597\_t001 |  | Vitvi01g01540\_t001 |  |  |  |  |  |  |  |  |  |  |
| 1 | Vitvi14g01598\_t002 |  | \| \| |  |  |  |  |  |  |  |  |  |  |
| 1 | Vitvi14g02991\_t001 |  | \| \| |  |  |  |  |  |  |  |  |  |  |
| 2 | Vitvi14g01600\_t001 |  | Vitvi01g01538\_t001 |  | Vitvi17g01598\_t002 |  |  |  |  |  |  |  |  |
| 2 | Vitvi14g01602\_t001 |  | Vitvi01g01533\_t002 |  | Vitvi17g01050\_t001 |  |  |  |  |  |  |  |  |
| 2 | Vitvi14g01603\_t001 |  | \| \| |  | \| \| |  |  |  |  |  |  |  |  |
| 2 | Vitvi14g01604\_t001 |  | Vitvi01g01529\_t001 |  | \| \| |  |  |  |  |  |  |  |  |
| 2 | Vitvi14g01605\_t005 |  | \| \| |  | \| \| |  |  |  |  |  |  |  |  |
| 2 | Vitvi14g01606\_t002 |  | \| \| |  | \| \| |  |  |  |  |  |  |  |  |
| 2 | Vitvi14g04599\_t001 |  | \| \| |  | \| \| |  |  |  |  |  |  |  |  |
| 2 | Vitvi14g01607\_t002 |  | \| \| |  | \| \| |  |  |  |  |  |  |  |  |
| 2 | Vitvi14g04600\_t001 |  | \| \| |  | \| \| |  |  |  |  |  |  |  |  |
| 2 | Vitvi14g04601\_t001 |  | Vitvi01g01521\_t001 |  | \| \| |  |  |  |  |  |  |  |  |
| 2 | Vitvi14g04602\_t001 |  | \| \| |  | \| \| |  |  |  |  |  |  |  |  |
| 2 | Vitvi14g01610\_t001 |  | Vitvi01g01508\_t001 |  | \| \| |  |  |  |  |  |  |  |  |
| 2 | Vitvi14g04603\_t001 |  | \| \| |  | \| \| |  |  |  |  |  |  |  |  |
| 2 | Vitvi14g04604\_t001 |  | \| \| |  | \| \| |  |  |  |  |  |  |  |  |
| 2 | Vitvi14g01611\_t001 |  | \| \| |  | \| \| |  |  |  |  |  |  |  |  |
| 2 | Vitvi14g01614\_t001 |  | \| \| |  | \| \| |  |  |  |  |  |  |  |  |
| 2 | Vitvi14g01615\_t001 |  | \| \| |  | \| \| |  |  |  |  |  |  |  |  |
| 2 | Vitvi14g01616\_t001 |  | \| \| |  | \| \| |  |  |  |  |  |  |  |  |
| 2 | Vitvi14g01617\_t001 |  | \| \| |  | Vitvi17g01024\_t001 |  |  |  |  |  |  |  |  |
| 2 | Vitvi14g01620\_t001 |  | \| \| |  | \| \| |  |  |  |  |  |  |  |  |
| 2 | Vitvi14g01621\_t001 |  | Vitvi01g01507\_t001 |  | \| \| |  |  |  |  |  |  |  |  |
| 1 | Vitvi14g01622\_t001 |  |  |  | \| \| |  |  |  |  |  |  |  |  |
| 1 | Vitvi14g01623\_t001 |  |  |  | \| \| |  |  |  |  |  |  |  |  |
| 1 | Vitvi14g01624\_t003 |  |  |  | \| \| |  |  |  |  |  |  |  |  |
| 1 | Vitvi14g01626\_t001 |  |  |  | \| \| |  |  |  |  |  |  |  |  |
| 1 | Vitvi14g04605\_t001 |  |  |  | \| \| |  |  |  |  |  |  |  |  |
| 1 | Vitvi14g01627\_t001 |  |  |  | \| \| |  |  |  |  |  |  |  |  |
| 1 | Vitvi14g01628\_t001 |  |  |  | Vitvi17g01003\_t001 |  |  |  |  |  |  |  |  |
| 1 | Vitvi14g01629\_t001 |  |  |  | Vitvi17g01002\_t001 |  |  |  |  |  |  |  |  |
| 1 | Vitvi14g01630\_t001 |  |  |  | \| \| |  |  |  |  |  |  |  |  |
| 1 | Vitvi14g01631\_t001 |  |  |  | \| \| |  |  |  |  |  |  |  |  |
| 1 | Vitvi14g04606\_t001 |  |  |  | \| \| |  |  |  |  |  |  |  |  |
| 1 | Vitvi14g01632\_t001 |  |  |  | \| \| |  |  |  |  |  |  |  |  |
| 1 | Vitvi14g01633\_t001 |  |  |  | \| \| |  |  |  |  |  |  |  |  |
| 1 | Vitvi14g01634\_t001 |  |  |  | \| \| |  |  |  |  |  |  |  |  |
| 2 | Vitvi14g01635\_t001 |  | Vitvi01g00593\_t001 |  | Vitvi17g00977\_t001 |  |  |  |  |  |  |  |  |
| 2 | Vitvi14g02994\_t001 |  | \| \| |  | \| \| |  |  |  |  |  |  |  |  |
| 2 | Vitvi14g01636\_t001 |  | \| \| |  | \| \| |  |  |  |  |  |  |  |  |
| 2 | Vitvi14g02995\_t001 |  | \| \| |  | \| \| |  |  |  |  |  |  |  |  |
| 2 | Vitvi14g01637\_t001 |  | Vitvi01g00602\_t001 |  | \| \| |  |  |  |  |  |  |  |  |
| 2 | Vitvi14g01639\_t001 |  | \| \| |  | \| \| |  |  |  |  |  |  |  |  |
| 2 | Vitvi14g01640\_t001 |  | \| \| |  | Vitvi17g00969\_t001 |  |  |  |  |  |  |  |  |
| 2 | Vitvi14g02996\_t001 |  | Vitvi01g02022\_t001 |  | \| \| |  |  |  |  |  |  |  |  |
| 2 | Vitvi14g01641\_t001 |  | Vitvi01g00609\_t001 |  | Vitvi17g00963\_t001 |  |  |  |  |  |  |  |  |
| 2 | Vitvi14g01642\_t001 |  | \| \| |  | \| \| |  |  |  |  |  |  |  |  |
| 2 | Vitvi14g01646\_t002 |  | \| \| |  | \| \| |  |  |  |  |  |  |  |  |
| 2 | Vitvi14g04607\_t001 |  | \| \| |  | \| \| |  |  |  |  |  |  |  |  |
| 2 | Vitvi14g01650\_t001 |  | \| \| |  | \| \| |  |  |  |  |  |  |  |  |
| 2 | Vitvi14g02997\_t001 |  | \| \| |  | \| \| |  |  |  |  |  |  |  |  |
| 2 | Vitvi14g04608\_t001 |  | \| \| |  | \| \| |  |  |  |  |  |  |  |  |
| 2 | Vitvi14g01653\_t001 |  | Vitvi01g00611\_t001 |  | \| \| |  |  |  |  |  |  |  |  |
| 2 | Vitvi14g01655\_t001 |  | \| \| |  | Vitvi17g00958\_t001 |  |  |  |  |  |  |  |  |
| 2 | Vitvi14g02998\_t001 |  | \| \| |  | \| \| |  |  |  |  |  |  |  |  |
| 2 | Vitvi14g01656\_t001 |  | \| \| |  | \| \| |  |  |  |  |  |  |  |  |
| 2 | Vitvi14g01657\_t001 |  | \| \| |  | \| \| |  |  |  |  |  |  |  |  |
| 2 | Vitvi14g01658\_t001 |  | \| \| |  | \| \| |  |  |  |  |  |  |  |  |
| 2 | Vitvi14g04609\_t001 |  | \| \| |  | \| \| |  |  |  |  |  |  |  |  |
| 2 | Vitvi14g01660\_t001 |  | \| \| |  | \| \| |  |  |  |  |  |  |  |  |
| 2 | Vitvi14g01661\_t001 |  | \| \| |  | \| \| |  |  |  |  |  |  |  |  |
| 3 | Vitvi14g01662\_t001 |  | \| \| |  | \| \| |  | Vitvi17g00928\_t001 |  |  |  |  |  |  |
| 4 | Vitvi14g04610\_t001 |  | \| \| |  | \| \| |  | \| \| |  | Vitvi01g00634\_t001 |  |  |  |  |
| 4 | Vitvi14g04611\_t001 |  | \| \| |  | \| \| |  | \| \| |  | \| \| |  |  |  |  |
| 4 | Vitvi14g04612\_t001 |  | \| \| |  | \| \| |  | \| \| |  | Vitvi01g00635\_t001 |  |  |  |  |
| 4 | Vitvi14g01664\_t001 |  | \| \| |  | \| \| |  | \| \| |  | Vitvi01g00637\_t001 |  |  |  |  |
| 4 | Vitvi14g01665\_t001 |  | \| \| |  | \| \| |  | \| \| |  | \| \| |  |  |  |  |
| 4 | Vitvi14g01666\_t001 |  | \| \| |  | \| \| |  | \| \| |  | \| \| |  |  |  |  |
| 4 | Vitvi14g01667\_t001 |  | \| \| |  | \| \| |  | \| \| |  | \| \| |  |  |  |  |
| 4 | Vitvi14g01668\_t001 |  | \| \| |  | \| \| |  | \| \| |  | \| \| |  |  |  |  |
| 4 | Vitvi14g04613\_t001 |  | \| \| |  | \| \| |  | \| \| |  | \| \| |  |  |  |  |
| 4 | Vitvi14g04614\_t001 |  | \| \| |  | \| \| |  | \| \| |  | \| \| |  |  |  |  |
| 4 | Vitvi14g01671\_t001 |  | Vitvi01g00614\_t001 |  | Vitvi17g00954\_t001 |  | \| \| |  | \| \| |  |  |  |  |
| 2 | Vitvi14g03000\_t001 |  |  |  |  |  | \| \| |  | \| \| |  |  |  |  |
| 2 | Vitvi14g01672\_t001 |  |  |  |  |  | \| \| |  | Vitvi01g00641\_t001 |  |  |  |  |
| 2 | Vitvi14g01673\_t001 |  |  |  |  |  | \| \| |  | \| \| |  |  |  |  |
| 2 | Vitvi14g01674\_t001 |  |  |  |  |  | \| \| |  | \| \| |  |  |  |  |
| 2 | Vitvi14g01675\_t001 |  |  |  |  |  | \| \| |  | \| \| |  |  |  |  |
| 2 | Vitvi14g01676\_t002 |  |  |  |  |  | \| \| |  | Vitvi01g00642\_t001 |  |  |  |  |
| 2 | Vitvi14g01677\_t001 |  |  |  |  |  | \| \| |  | \| \| |  |  |  |  |
| 2 | Vitvi14g01678\_t001 |  |  |  |  |  | \| \| |  | \| \| |  |  |  |  |
| 2 | Vitvi14g01679\_t001 |  |  |  |  |  | Vitvi17g00917\_t001 |  | \| \| |  |  |  |  |
| 2 | Vitvi14g04615\_t001 |  |  |  |  |  | \| \| |  | \| \| |  |  |  |  |
| 2 | Vitvi14g01682\_t001 |  |  |  |  |  | \| \| |  | \| \| |  |  |  |  |
| 2 | Vitvi14g01683\_t001 |  |  |  |  |  | \| \| |  | \| \| |  |  |  |  |
| 2 | Vitvi14g01684\_t001.2.6037826b |  |  |  |  |  | \| \| |  | \| \| |  |  |  |  |
| 2 | Vitvi14g01685\_t001 |  |  |  |  |  | \| \| |  | \| \| |  |  |  |  |
| 2 | Vitvi14g01686\_t001 |  |  |  |  |  | \| \| |  | \| \| |  |  |  |  |
| 2 | Vitvi14g01687\_t001 |  |  |  |  |  | \| \| |  | \| \| |  |  |  |  |
| 2 | Vitvi14g01688\_t001 |  |  |  |  |  | \| \| |  | \| \| |  |  |  |  |
| 2 | Vitvi14g01689\_t001 |  |  |  |  |  | \| \| |  | \| \| |  |  |  |  |
| 2 | Vitvi14g01690\_t001 |  |  |  |  |  | \| \| |  | \| \| |  |  |  |  |
| 2 | Vitvi14g01691\_t001 |  |  |  |  |  | \| \| |  | \| \| |  |  |  |  |
| 2 | Vitvi14g01692\_t001 |  |  |  |  |  | \| \| |  | \| \| |  |  |  |  |
| 2 | Vitvi14g01693\_t002 |  |  |  |  |  | \| \| |  | \| \| |  |  |  |  |
| 2 | Vitvi14g03001\_t001 |  |  |  |  |  | \| \| |  | \| \| |  |  |  |  |
| 2 | Vitvi14g01694\_t001 |  |  |  |  |  | \| \| |  | \| \| |  |  |  |  |
| 2 | Vitvi14g01695\_t001 |  |  |  |  |  | \| \| |  | \| \| |  |  |  |  |
| 2 | Vitvi14g01696\_t001 |  |  |  |  |  | \| \| |  | Vitvi01g00650\_t001 |  |  |  |  |
| 2 | Vitvi14g04616\_t001 |  |  |  |  |  | \| \| |  | \| \| |  |  |  |  |
| 2 | Vitvi14g01698\_t001 |  |  |  |  |  | Vitvi17g00899\_t001 |  | Vitvi01g04157\_t001 |  |  |  |  |
| 2 | Vitvi14g01699\_t001 |  |  |  |  |  | \| \| |  | \| \| |  |  |  |  |
| 2 | Vitvi14g01700\_t001 |  |  |  |  |  | \| \| |  | \| \| |  |  |  |  |
| 2 | Vitvi14g03003\_t001 |  |  |  |  |  | \| \| |  | Vitvi01g02039\_t001 |  |  |  |  |
| 2 | Vitvi14g01701\_t001 |  |  |  |  |  | \| \| |  | \| \| |  |  |  |  |
| 2 | Vitvi14g01702\_t001 |  |  |  |  |  | \| \| |  | \| \| |  |  |  |  |
| 2 | Vitvi14g03004\_t001 |  |  |  |  |  | \| \| |  | \| \| |  |  |  |  |
| 2 | Vitvi14g01703\_t002 |  |  |  |  |  | \| \| |  | \| \| |  |  |  |  |
| 2 | Vitvi14g01704\_t001 |  |  |  |  |  | \| \| |  | \| \| |  |  |  |  |
| 2 | Vitvi14g01706\_t001 |  |  |  |  |  | \| \| |  | \| \| |  |  |  |  |
| 2 | Vitvi14g01707\_t001 |  |  |  |  |  | Vitvi17g00890\_t002 |  | \| \| |  |  |  |  |
| 2 | Vitvi14g04617\_t001 |  |  |  |  |  | \| \| |  | \| \| |  |  |  |  |
| 2 | Vitvi14g03008\_t001 |  |  |  |  |  | Vitvi17g01554\_t001 |  | \| \| |  |  |  |  |
| 2 | Vitvi14g01708\_t001 |  |  |  |  |  | \| \| |  | \| \| |  |  |  |  |
| 2 | Vitvi14g03009\_t001 |  |  |  |  |  | \| \| |  | \| \| |  |  |  |  |
| 2 | Vitvi14g01709\_t001 |  |  |  |  |  | \| \| |  | \| \| |  |  |  |  |
| 2 | Vitvi14g04618\_t001 |  |  |  |  |  | \| \| |  | \| \| |  |  |  |  |
| 2 | Vitvi14g04619\_t001 |  |  |  |  |  | \| \| |  | \| \| |  |  |  |  |
| 2 | Vitvi14g03010\_t001 |  |  |  |  |  | \| \| |  | Vitvi01g04159\_t001 |  |  |  |  |
| 2 | Vitvi14g03011\_t001 |  |  |  |  |  | \| \| |  | Vitvi01g04160\_t001 |  |  |  |  |
| 2 | Vitvi14g04620\_t001 |  |  |  |  |  | \| \| |  | \| \| |  |  |  |  |
| 2 | Vitvi14g04621\_t001 |  |  |  |  |  | \| \| |  | \| \| |  |  |  |  |
| 2 | Vitvi14g03012\_t001 |  |  |  |  |  | \| \| |  | \| \| |  |  |  |  |
| 2 | Vitvi14g04622\_t001 |  |  |  |  |  | \| \| |  | \| \| |  |  |  |  |
| 2 | Vitvi14g03013\_t001 |  |  |  |  |  | \| \| |  | \| \| |  |  |  |  |
| 2 | Vitvi14g03015\_t001 |  |  |  |  |  | \| \| |  | \| \| |  |  |  |  |
| 2 | Vitvi14g04623\_t001 |  |  |  |  |  | \| \| |  | \| \| |  |  |  |  |
| 2 | Vitvi14g01714\_t001 |  |  |  |  |  | \| \| |  | \| \| |  |  |  |  |
| 2 | Vitvi14g01715\_t001 |  |  |  |  |  | Vitvi17g00886\_t001 |  | \| \| |  |  |  |  |
| 2 | Vitvi14g01717\_t001 |  |  |  |  |  | Vitvi17g00885\_t001 |  | \| \| |  |  |  |  |
| 2 | Vitvi14g01718\_t001 |  |  |  |  |  | \| \| |  | \| \| |  |  |  |  |
| 2 | Vitvi14g01719\_t001 |  |  |  |  |  | \| \| |  | \| \| |  |  |  |  |
| 2 | Vitvi14g03016\_t001 |  |  |  |  |  | \| \| |  | \| \| |  |  |  |  |
| 2 | Vitvi14g01720\_t001 |  |  |  |  |  | \| \| |  | Vitvi01g00682\_t001 |  |  |  |  |
| 2 | Vitvi14g01721\_t001 |  |  |  |  |  | \| \| |  | \| \| |  |  |  |  |
| 2 | Vitvi14g01722\_t001 |  |  |  |  |  | \| \| |  | \| \| |  |  |  |  |
| 2 | Vitvi14g04624\_t001 |  |  |  |  |  | \| \| |  | \| \| |  |  |  |  |
| 2 | Vitvi14g01723\_t001 |  |  |  |  |  | \| \| |  | \| \| |  |  |  |  |
| 2 | Vitvi14g04625\_t001 |  |  |  |  |  | \| \| |  | \| \| |  |  |  |  |
| 2 | Vitvi14g01726\_t001 |  |  |  |  |  | \| \| |  | \| \| |  |  |  |  |
| 2 | Vitvi14g01727\_t001 |  |  |  |  |  | Vitvi17g00863\_t001 |  | Vitvi01g00685\_t001 |  |  |  |  |
| 2 | Vitvi14g04626\_t001 |  |  |  |  |  | \| \| |  | \| \| |  |  |  |  |
| 2 | Vitvi14g01728\_t001 |  |  |  |  |  | \| \| |  | \| \| |  |  |  |  |
| 2 | Vitvi14g01730\_t001 |  |  |  |  |  | \| \| |  | \| \| |  |  |  |  |
| 2 | Vitvi14g04627\_t001 |  |  |  |  |  | \| \| |  | \| \| |  |  |  |  |
| 2 | Vitvi14g01732\_t001 |  |  |  |  |  | \| \| |  | \| \| |  |  |  |  |
| 2 | Vitvi14g01734\_t001 |  |  |  |  |  | Vitvi17g00844\_t001 |  | \| \| |  |  |  |  |
| 2 | Vitvi14g01735\_t001 |  |  |  |  |  | Vitvi17g00843\_t001 |  | \| \| |  |  |  |  |
| 2 | Vitvi14g01736\_t001 |  |  |  |  |  | Vitvi17g00837\_t003 |  | Vitvi01g00701\_t001 |  |  |  |  |
| 2 | Vitvi14g01737\_t001 |  |  |  |  |  | \| \| |  | \| \| |  |  |  |  |
| 2 | Vitvi14g01739\_t001 |  |  |  |  |  | Vitvi17g00833\_t001 |  | \| \| |  |  |  |  |
| 2 | Vitvi14g01740\_t001 |  |  |  |  |  | Vitvi17g00832\_t001 |  | \| \| |  |  |  |  |
| 2 | Vitvi14g01741\_t001 |  |  |  |  |  | \| \| |  | \| \| |  |  |  |  |
| 2 | Vitvi14g01742\_t002 |  |  |  |  |  | \| \| |  | \| \| |  |  |  |  |
| 2 | Vitvi14g01743\_t001 |  |  |  |  |  | \| \| |  | \| \| |  |  |  |  |
| 2 | Vitvi14g01744\_t001 |  |  |  |  |  | \| \| |  | \| \| |  |  |  |  |
| 2 | Vitvi14g01745\_t001 |  |  |  |  |  | \| \| |  | Vitvi01g00710\_t001 |  |  |  |  |
| 2 | Vitvi14g01746\_t001 |  |  |  |  |  | \| \| |  | \| \| |  |  |  |  |
| 2 | Vitvi14g01748\_t001 |  |  |  |  |  | \| \| |  | Vitvi01g04177\_t002 |  |  |  |  |
| 2 | Vitvi14g01750\_t001 |  |  |  |  |  | \| \| |  | \| \| |  |  |  |  |
| 2 | Vitvi14g01751\_t001 |  |  |  |  |  | \| \| |  | \| \| |  |  |  |  |
| 2 | Vitvi14g01754\_t001 |  |  |  |  |  | Vitvi17g00824\_t001 |  | \| \| |  |  |  |  |
| 2 | Vitvi14g01756\_t001 |  |  |  |  |  | \| \| |  | \| \| |  |  |  |  |
| 2 | Vitvi14g03017\_t003 |  |  |  |  |  | \| \| |  | \| \| |  |  |  |  |
| 2 | Vitvi14g03018\_t001 |  |  |  |  |  | \| \| |  | \| \| |  |  |  |  |
| 2 | Vitvi14g01757\_t001 |  |  |  |  |  | \| \| |  | \| \| |  |  |  |  |
| 2 | Vitvi14g03019\_t001 |  |  |  |  |  | \| \| |  | \| \| |  |  |  |  |
| 2 | Vitvi14g01758\_t003 |  |  |  |  |  | \| \| |  | Vitvi01g00730\_t001 |  |  |  |  |
| 2 | Vitvi14g01760\_t001 |  |  |  |  |  | \| \| |  | \| \| |  |  |  |  |
| 2 | Vitvi14g01761\_t001 |  |  |  |  |  | Vitvi17g00815\_t001 |  | Vitvi01g00732\_t001 |  |  |  |  |
| 2 | Vitvi14g01762\_t002 |  |  |  |  |  | \| \| |  | \| \| |  |  |  |  |
| 2 | Vitvi14g01763\_t001 |  |  |  |  |  | \| \| |  | Vitvi01g00735\_t001 |  |  |  |  |
| 2 | Vitvi14g03020\_t001 |  |  |  |  |  | \| \| |  | Vitvi01g00737\_t001 |  |  |  |  |
| 2 | Vitvi14g01764\_t001 |  |  |  |  |  | \| \| |  | \| \| |  |  |  |  |
| 2 | Vitvi14g04628\_t001 |  |  |  |  |  | \| \| |  | \| \| |  |  |  |  |
| 2 | Vitvi14g01765\_t001 |  |  |  |  |  | \| \| |  | \| \| |  |  |  |  |
| 2 | Vitvi14g01766\_t001 |  |  |  |  |  | \| \| |  | \| \| |  |  |  |  |
| 2 | Vitvi14g04629\_t001 |  |  |  |  |  | \| \| |  | \| \| |  |  |  |  |
| 2 | Vitvi14g01769\_t001.1.6037826b |  |  |  |  |  | Vitvi17g00807\_t002 |  | \| \| |  |  |  |  |
| 2 | Vitvi14g01770\_t001 |  |  |  |  |  | \| \| |  | \| \| |  |  |  |  |
| 2 | Vitvi14g01771\_t001 |  |  |  |  |  | Vitvi17g00803\_t001 |  | \| \| |  |  |  |  |
| 2 | Vitvi14g01772\_t001 |  |  |  |  |  | \| \| |  | \| \| |  |  |  |  |
| 2 | Vitvi14g01773\_t001 |  |  |  |  |  | \| \| |  | \| \| |  |  |  |  |
| 2 | Vitvi14g01775\_t001 |  |  |  |  |  | \| \| |  | Vitvi01g00744\_t002 |  |  |  |  |
| 2 | Vitvi14g01776\_t001 |  |  |  |  |  | \| \| |  | \| \| |  |  |  |  |
| 2 | Vitvi14g01777\_t001 |  |  |  |  |  | \| \| |  | \| \| |  |  |  |  |
| 2 | Vitvi14g01778\_t001 |  |  |  |  |  | \| \| |  | \| \| |  |  |  |  |
| 2 | Vitvi14g01780\_t001 |  |  |  |  |  | \| \| |  | Vitvi01g00775\_t003 |  |  |  |  |
| 2 | Vitvi14g01781\_t001 |  |  |  |  |  | Vitvi17g00794\_t001 |  | \| \| |  |  |  |  |
| 2 | Vitvi14g01782\_t001 |  |  |  |  |  | \| \| |  | \| \| |  |  |  |  |
| 2 | Vitvi14g01783\_t001 |  |  |  |  |  | Vitvi17g00791\_t001 |  | \| \| |  |  |  |  |
| 2 | Vitvi14g01785\_t001 |  |  |  |  |  | \| \| |  | \| \| |  |  |  |  |
| 2 | Vitvi14g01786\_t001 |  |  |  |  |  | \| \| |  | \| \| |  |  |  |  |
| 2 | Vitvi14g01787\_t001 |  |  |  |  |  | \| \| |  | Vitvi01g00779\_t001 |  |  |  |  |
| 2 | Vitvi14g04630\_t001 |  |  |  |  |  | \| \| |  | \| \| |  |  |  |  |
| 2 | Vitvi14g01789\_t001 |  |  |  |  |  | Vitvi17g00790\_t001 |  | \| \| |  |  |  |  |
| 2 | Vitvi14g04631\_t001 |  |  |  |  |  | \| \| |  | \| \| |  |  |  |  |
| 2 | Vitvi14g03025\_t001 |  |  |  |  |  | Vitvi17g00788\_t001 |  | \| \| |  |  |  |  |
| 2 | Vitvi14g04632\_t001 |  |  |  |  |  | \| \| |  | \| \| |  |  |  |  |
| 2 | Vitvi14g01792\_t001 |  |  |  |  |  | \| \| |  | \| \| |  |  |  |  |
| 2 | Vitvi14g04633\_t001 |  |  |  |  |  | \| \| |  | \| \| |  |  |  |  |
| 2 | Vitvi14g01794\_t001 |  |  |  |  |  | Vitvi17g04230\_t001 |  | \| \| |  |  |  |  |
| 2 | Vitvi14g01795\_t001 |  |  |  |  |  | \| \| |  | Vitvi01g00781\_t001 |  |  |  |  |
| 2 | Vitvi14g01796\_t001 |  |  |  |  |  | Vitvi17g00786\_t001 |  | Vitvi01g00785\_t001 |  |  |  |  |
| 2 | Vitvi14g01797\_t001 |  |  |  |  |  | Vitvi17g00781\_t001 |  | \| \| |  |  |  |  |
| 2 | Vitvi14g04634\_t001 |  |  |  |  |  | \| \| |  | \| \| |  |  |  |  |
| 2 | Vitvi14g04635\_t001 |  |  |  |  |  | \| \| |  | Vitvi01g04200\_t001 |  |  |  |  |
| 2 | Vitvi14g01798\_t001 |  |  |  |  |  | \| \| |  | Vitvi01g00792\_t002 |  |  |  |  |
| 2 | Vitvi14g01799\_t001 |  |  |  |  |  | \| \| |  | \| \| |  |  |  |  |
| 2 | Vitvi14g04636\_t001 |  |  |  |  |  | \| \| |  | \| \| |  |  |  |  |
| 2 | Vitvi14g01800\_t001 |  |  |  |  |  | Vitvi17g01535\_t001 |  | \| \| |  |  |  |  |
| 2 | Vitvi14g01801\_t001 |  |  |  |  |  | Vitvi17g00775\_t001 |  | Vitvi01g04205\_t001 |  |  |  |  |
| 2 | Vitvi14g04637\_t001 |  |  |  |  |  | \| \| |  | \| \| |  |  |  |  |
| 2 | Vitvi14g01802\_t001 |  |  |  |  |  | Vitvi17g00773\_t001 |  | \| \| |  |  |  |  |
| 2 | Vitvi14g01803\_t001 |  |  |  |  |  | Vitvi17g00767\_t001 |  | \| \| |  |  |  |  |
| 2 | Vitvi14g01804\_t001 |  |  |  |  |  | \| \| |  | \| \| |  |  |  |  |
| 2 | Vitvi14g01805\_t001 |  |  |  |  |  | Vitvi17g00765\_t001 |  | Vitvi01g00803\_t002 |  |  |  |  |
| 2 | Vitvi14g01806\_t001 |  |  |  |  |  | \| \| |  | Vitvi01g00812\_t001 |  |  |  |  |
| 2 | Vitvi14g04638\_t001 |  |  |  |  |  | \| \| |  | \| \| |  |  |  |  |
| 2 | Vitvi14g01807\_t001 |  |  |  |  |  | Vitvi17g00764\_t001 |  | \| \| |  |  |  |  |
| 2 | Vitvi14g01808\_t001 |  |  |  |  |  | \| \| |  | \| \| |  |  |  |  |
| 2 | Vitvi14g01809\_t001 |  |  |  |  |  | \| \| |  | \| \| |  |  |  |  |
| 2 | Vitvi14g04639\_t001 |  |  |  |  |  | \| \| |  | \| \| |  |  |  |  |
| 2 | Vitvi14g01811\_t001 |  |  |  |  |  | \| \| |  | \| \| |  |  |  |  |
| 2 | Vitvi14g01812\_t002 |  |  |  |  |  | Vitvi17g00761\_t001 |  | \| \| |  |  |  |  |
| 2 | Vitvi14g01815\_t001 |  |  |  |  |  | \| \| |  | \| \| |  |  |  |  |
| 2 | Vitvi14g01816\_t001 |  |  |  |  |  | \| \| |  | \| \| |  |  |  |  |
| 2 | Vitvi14g01817\_t001 |  |  |  |  |  | \| \| |  | \| \| |  |  |  |  |
| 2 | Vitvi14g01818\_t002 |  |  |  |  |  | \| \| |  | Vitvi01g04213\_t001 |  |  |  |  |
| 2 | Vitvi14g03029\_t001 |  |  |  |  |  | \| \| |  | \| \| |  |  |  |  |
| 2 | Vitvi14g01819\_t001 |  |  |  |  |  | Vitvi17g00750\_t001 |  | \| \| |  |  |  |  |
| 2 | Vitvi14g04640\_t001 |  |  |  |  |  | \| \| |  | \| \| |  |  |  |  |
| 2 | Vitvi14g01820\_t001 |  |  |  |  |  | \| \| |  | \| \| |  |  |  |  |
| 2 | Vitvi14g03030\_t001 |  |  |  |  |  | \| \| |  | \| \| |  |  |  |  |
| 2 | Vitvi14g03031\_t001 |  |  |  |  |  | \| \| |  | \| \| |  |  |  |  |
| 2 | Vitvi14g04641\_t001 |  |  |  |  |  | \| \| |  | \| \| |  |  |  |  |
| 2 | Vitvi14g04642\_t001 |  |  |  |  |  | \| \| |  | \| \| |  |  |  |  |
| 2 | Vitvi14g03032\_t001 |  |  |  |  |  | \| \| |  | \| \| |  |  |  |  |
| 2 | Vitvi14g04643\_t001 |  |  |  |  |  | \| \| |  | \| \| |  |  |  |  |
| 2 | Vitvi14g04644\_t001 |  |  |  |  |  | \| \| |  | \| \| |  |  |  |  |
| 2 | Vitvi14g03033\_t001 |  |  |  |  |  | \| \| |  | \| \| |  |  |  |  |
| 2 | Vitvi14g01821\_t001 |  |  |  |  |  | Vitvi17g00747\_t001 |  | Vitvi01g00844\_t001 |  |  |  |  |
| 2 | Vitvi14g01822\_t001 |  |  |  |  |  | Vitvi17g01530\_t001 |  | Vitvi01g00845\_t001 |  |  |  |  |
| 2 | Vitvi14g01823\_t001 |  |  |  |  |  | Vitvi17g00743\_t001 |  | \| \| |  |  |  |  |
| 2 | Vitvi14g01824\_t001 |  |  |  |  |  | \| \| |  | \| \| |  |  |  |  |
| 2 | Vitvi14g01825\_t001 |  |  |  |  |  | Vitvi17g00742\_t001 |  | Vitvi01g00846\_t002 |  |  |  |  |
| 1 | Vitvi14g01826\_t001 |  |  |  |  |  |  |  | \| \| |  |  |  |  |
| 1 | Vitvi14g01827\_t002 |  |  |  |  |  |  |  | \| \| |  |  |  |  |
| 1 | Vitvi14g01828\_t001 |  |  |  |  |  |  |  | \| \| |  |  |  |  |
| 1 | Vitvi14g01829\_t001 |  |  |  |  |  |  |  | \| \| |  |  |  |  |
| 1 | Vitvi14g03035\_t001 |  |  |  |  |  |  |  | \| \| |  |  |  |  |
| 1 | Vitvi14g04645\_t001 |  |  |  |  |  |  |  | \| \| |  |  |  |  |
| 1 | Vitvi14g03036\_t001 |  |  |  |  |  |  |  | \| \| |  |  |  |  |
| 1 | Vitvi14g03037\_t001 |  |  |  |  |  |  |  | Vitvi01g00850\_t001 |  |  |  |  |
| 1 | Vitvi14g03038\_t001 |  |  |  |  |  |  |  | \| \| |  |  |  |  |
| 1 | Vitvi14g04646\_t001 |  |  |  |  |  |  |  | \| \| |  |  |  |  |
| 1 | Vitvi14g03039\_t001 |  |  |  |  |  |  |  | \| \| |  |  |  |  |
| 1 | Vitvi14g04647\_t001 |  |  |  |  |  |  |  | \| \| |  |  |  |  |
| 1 | Vitvi14g01831\_t001 |  |  |  |  |  |  |  | \| \| |  |  |  |  |
| 1 | Vitvi14g01832\_t001 |  |  |  |  |  |  |  | \| \| |  |  |  |  |
| 1 | Vitvi14g01833\_t001 |  |  |  |  |  |  |  | \| \| |  |  |  |  |
| 1 | Vitvi14g01835\_t001 |  |  |  |  |  |  |  | \| \| |  |  |  |  |
| 1 | Vitvi14g01837\_t001 |  |  |  |  |  |  |  | \| \| |  |  |  |  |
| 1 | Vitvi14g04648\_t001 |  |  |  |  |  |  |  | \| \| |  |  |  |  |
| 1 | Vitvi14g01838\_t001 |  |  |  |  |  |  |  | \| \| |  |  |  |  |
| 1 | Vitvi14g03041\_t001 |  |  |  |  |  |  |  | \| \| |  |  |  |  |
| 1 | Vitvi14g01840\_t001 |  |  |  |  |  |  |  | \| \| |  |  |  |  |
| 1 | Vitvi14g03042\_t001 |  |  |  |  |  |  |  | \| \| |  |  |  |  |
| 1 | Vitvi14g03043\_t001 |  |  |  |  |  |  |  | \| \| |  |  |  |  |
| 1 | Vitvi14g03044\_t001 |  |  |  |  |  |  |  | \| \| |  |  |  |  |
| 1 | Vitvi14g03045\_t001 |  |  |  |  |  |  |  | \| \| |  |  |  |  |
| 1 | Vitvi14g01841\_t001 |  |  |  |  |  |  |  | \| \| |  |  |  |  |
| 1 | Vitvi14g01842\_t001 |  |  |  |  |  |  |  | \| \| |  |  |  |  |
| 1 | Vitvi14g01843\_t001 |  |  |  |  |  |  |  | \| \| |  |  |  |  |
| 1 | Vitvi14g03047\_t001 |  |  |  |  |  |  |  | \| \| |  |  |  |  |
| 1 | Vitvi14g01845\_t001 |  |  |  |  |  |  |  | \| \| |  |  |  |  |
| 1 | Vitvi14g04649\_t001 |  |  |  |  |  |  |  | \| \| |  |  |  |  |
| 1 | Vitvi14g01846\_t001 |  |  |  |  |  |  |  | \| \| |  |  |  |  |
| 1 | Vitvi14g01847\_t001 |  |  |  |  |  |  |  | \| \| |  |  |  |  |
| 1 | Vitvi14g04650\_t001 |  |  |  |  |  |  |  | Vitvi01g02100\_t001 |  |  |  |  |
| 0 | Vitvi14g04651\_t002 |  |  |  |  |  |  |  |  |  |  |  |  |
| 0 | Vitvi14g01852\_t002 |  |  |  |  |  |  |  |  |  |  |  |  |
| 0 | Vitvi14g01853\_t001 |  |  |  |  |  |  |  |  |  |  |  |  |
| 1 | Vitvi14g01855\_t001 |  | Vitvi17g00724\_t001 |  |  |  |  |  |  |  |  |  |  |
| 1 | Vitvi14g04652\_t001 |  | \| \| |  |  |  |  |  |  |  |  |  |  |
| 1 | Vitvi14g03048\_t001 |  | \| \| |  |  |  |  |  |  |  |  |  |  |
| 1 | Vitvi14g01856\_t001 |  | \| \| |  |  |  |  |  |  |  |  |  |  |
| 1 | Vitvi14g01857\_t001 |  | \| \| |  |  |  |  |  |  |  |  |  |  |
| 1 | Vitvi14g01858\_t001 |  | \| \| |  |  |  |  |  |  |  |  |  |  |
| 1 | Vitvi14g04653\_t001 |  | \| \| |  |  |  |  |  |  |  |  |  |  |
| 1 | Vitvi14g01859\_t001 |  | \| \| |  |  |  |  |  |  |  |  |  |  |
| 1 | Vitvi14g01860\_t001 |  | \| \| |  |  |  |  |  |  |  |  |  |  |
| 1 | Vitvi14g04654\_t001 |  | \| \| |  |  |  |  |  |  |  |  |  |  |
| 1 | Vitvi14g04655\_t001 |  | \| \| |  |  |  |  |  |  |  |  |  |  |
| 1 | Vitvi14g04656\_t001 |  | \| \| |  |  |  |  |  |  |  |  |  |  |
| 1 | Vitvi14g04657\_t001 |  | \| \| |  |  |  |  |  |  |  |  |  |  |
| 1 | Vitvi14g04658\_t001 |  | \| \| |  |  |  |  |  |  |  |  |  |  |
| 1 | Vitvi14g01865\_t001 |  | \| \| |  |  |  |  |  |  |  |  |  |  |
| 2 | Vitvi14g01866\_t001 |  | Vitvi17g01522\_t001 |  | Vitvi01g02098\_t001 |  |  |  |  |  |  |  |  |
| 2 | Vitvi14g01867\_t001 |  | Vitvi17g00730\_t002 |  | \| \| |  |  |  |  |  |  |  |  |
| 2 | Vitvi14g01868\_t001 |  | \| \| |  | \| \| |  |  |  |  |  |  |  |  |
| 2 | Vitvi14g01869\_t001 |  | \| \| |  | \| \| |  |  |  |  |  |  |  |  |
| 2 | Vitvi14g01870\_t001 |  | Vitvi17g00736\_t002 |  | \| \| |  |  |  |  |  |  |  |  |
| 2 | Vitvi14g01871\_t001 |  | \| \| |  | \| \| |  |  |  |  |  |  |  |  |
| 2 | Vitvi14g01872\_t001 |  | \| \| |  | \| \| |  |  |  |  |  |  |  |  |
| 2 | Vitvi14g01873\_t001 |  | Vitvi17g00738\_t001 |  | \| \| |  |  |  |  |  |  |  |  |
| 2 | Vitvi14g01874\_t001 |  | Vitvi17g01525\_t001 |  | \| \| |  |  |  |  |  |  |  |  |
| 1 | Vitvi14g03049\_t001 |  |  |  | \| \| |  |  |  |  |  |  |  |  |
| 2 | Vitvi14g01875\_t001 |  | Vitvi17g01452\_t001 |  | \| \| |  |  |  |  |  |  |  |  |
| 2 | Vitvi14g03050\_t001 |  | \| \| |  | Vitvi01g00885\_t001 |  |  |  |  |  |  |  |  |
| 2 | Vitvi14g01877\_t001 |  | \| \| |  | \| \| |  |  |  |  |  |  |  |  |
| 2 | Vitvi14g04659\_t001 |  | \| \| |  | \| \| |  |  |  |  |  |  |  |  |
| 2 | Vitvi14g01878\_t001 |  | Vitvi17g00520\_t001 |  | \| \| |  |  |  |  |  |  |  |  |
| 2 | Vitvi14g01879\_t001 |  | \| \| |  | Vitvi01g00902\_t004 |  |  |  |  |  |  |  |  |
| 2 | Vitvi14g01881\_t001 |  | \| \| |  | \| \| |  |  |  |  |  |  |  |  |
| 2 | Vitvi14g01882\_t001 |  | Vitvi17g00521\_t001 |  | \| \| |  |  |  |  |  |  |  |  |
| 2 | Vitvi14g01883\_t001 |  | \| \| |  | \| \| |  |  |  |  |  |  |  |  |
| 2 | Vitvi14g03051\_t001 |  | \| \| |  | \| \| |  |  |  |  |  |  |  |  |
| 2 | Vitvi14g01884\_t001 |  | \| \| |  | \| \| |  |  |  |  |  |  |  |  |
| 2 | Vitvi14g01885\_t001 |  | \| \| |  | \| \| |  |  |  |  |  |  |  |  |
| 2 | Vitvi14g01886\_t001 |  | \| \| |  | \| \| |  |  |  |  |  |  |  |  |
| 2 | Vitvi14g01887\_t001 |  | \| \| |  | \| \| |  |  |  |  |  |  |  |  |
| 2 | Vitvi14g01888\_t001 |  | Vitvi17g00527\_t001 |  | \| \| |  |  |  |  |  |  |  |  |
| 2 | Vitvi14g01889\_t001.1.6037826b |  | \| \| |  | Vitvi01g00907\_t001 |  |  |  |  |  |  |  |  |
| 2 | Vitvi14g01890\_t001 |  | \| \| |  | \| \| |  |  |  |  |  |  |  |  |
| 2 | Vitvi14g01891\_t001 |  | \| \| |  | \| \| |  |  |  |  |  |  |  |  |
| 2 | Vitvi14g01892\_t001 |  | Vitvi17g00528\_t001 |  | Vitvi01g00911\_t001 |  |  |  |  |  |  |  |  |
| 2 | Vitvi14g01894\_t001 |  | \| \| |  | \| \| |  |  |  |  |  |  |  |  |
| 2 | Vitvi14g03052\_t001 |  | \| \| |  | \| \| |  |  |  |  |  |  |  |  |
| 2 | Vitvi14g04660\_t001 |  | \| \| |  | \| \| |  |  |  |  |  |  |  |  |
| 2 | Vitvi14g01895\_t001 |  | \| \| |  | \| \| |  |  |  |  |  |  |  |  |
| 2 | Vitvi14g01896\_t001 |  | \| \| |  | \| \| |  |  |  |  |  |  |  |  |
| 2 | Vitvi14g04661\_t001 |  | \| \| |  | \| \| |  |  |  |  |  |  |  |  |
| 2 | Vitvi14g03055\_t001 |  | \| \| |  | \| \| |  |  |  |  |  |  |  |  |
| 2 | Vitvi14g03056\_t001 |  | \| \| |  | \| \| |  |  |  |  |  |  |  |  |
| 2 | Vitvi14g03057\_t001 |  | \| \| |  | \| \| |  |  |  |  |  |  |  |  |
| 2 | Vitvi14g04662\_t001 |  | \| \| |  | \| \| |  |  |  |  |  |  |  |  |
| 2 | Vitvi14g04663\_t001 |  | \| \| |  | \| \| |  |  |  |  |  |  |  |  |
| 2 | Vitvi14g04664\_t001 |  | \| \| |  | \| \| |  |  |  |  |  |  |  |  |
| 2 | Vitvi14g01897\_t001 |  | \| \| |  | \| \| |  |  |  |  |  |  |  |  |
| 2 | Vitvi14g04665\_t001 |  | \| \| |  | \| \| |  |  |  |  |  |  |  |  |
| 2 | Vitvi14g01898\_t001 |  | Vitvi17g00547\_t001 |  | Vitvi01g00930\_t001 |  |  |  |  |  |  |  |  |
| 2 | Vitvi14g01899\_t001 |  | \| \| |  | \| \| |  |  |  |  |  |  |  |  |
| 2 | Vitvi14g01900\_t001 |  | Vitvi17g00550\_t001 |  | Vitvi01g02109\_t001 |  |  |  |  |  |  |  |  |
| 2 | Vitvi14g01901\_t001 |  | \| \| |  | \| \| |  |  |  |  |  |  |  |  |
| 2 | Vitvi14g01902\_t001 |  | \| \| |  | Vitvi01g00934\_t001 |  |  |  |  |  |  |  |  |
| 2 | Vitvi14g01903\_t001 |  | \| \| |  | \| \| |  |  |  |  |  |  |  |  |
| 2 | Vitvi14g03059\_t001 |  | \| \| |  | \| \| |  |  |  |  |  |  |  |  |
| 2 | Vitvi14g01905\_t001 |  | \| \| |  | \| \| |  |  |  |  |  |  |  |  |
| 2 | Vitvi14g01906\_t001 |  | Vitvi17g00554\_t001 |  | \| \| |  |  |  |  |  |  |  |  |
| 2 | Vitvi14g03060\_t001 |  | \| \| |  | \| \| |  |  |  |  |  |  |  |  |
| 2 | Vitvi14g03061\_t001 |  | \| \| |  | \| \| |  |  |  |  |  |  |  |  |
| 2 | Vitvi14g01907\_t001 |  | Vitvi17g00556\_t001 |  | Vitvi01g00940\_t001 |  |  |  |  |  |  |  |  |
| 2 | Vitvi14g01908\_t001 |  | \| \| |  | \| \| |  |  |  |  |  |  |  |  |
| 2 | Vitvi14g04666\_t001 |  | \| \| |  | \| \| |  |  |  |  |  |  |  |  |
| 2 | Vitvi14g01909\_t003 |  | Vitvi17g04153\_t001 |  | Vitvi01g00943\_t002 |  |  |  |  |  |  |  |  |
| 2 | Vitvi14g04667\_t001 |  | \| \| |  | \| \| |  |  |  |  |  |  |  |  |
| 2 | Vitvi14g01910\_t001 |  | Vitvi17g00560\_t001 |  | Vitvi01g04227\_t001 |  |  |  |  |  |  |  |  |
| 2 | Vitvi14g03062\_t001 |  | \| \| |  | Vitvi01g00950\_t001 |  |  |  |  |  |  |  |  |
| 2 | Vitvi14g04668\_t001 |  | \| \| |  | \| \| |  |  |  |  |  |  |  |  |
| 2 | Vitvi14g04669\_t001 |  | \| \| |  | \| \| |  |  |  |  |  |  |  |  |
| 2 | Vitvi14g03065\_t001 |  | \| \| |  | \| \| |  |  |  |  |  |  |  |  |
| 2 | Vitvi14g04670\_t001 |  | \| \| |  | \| \| |  |  |  |  |  |  |  |  |
| 2 | Vitvi14g03068\_t001 |  | \| \| |  | \| \| |  |  |  |  |  |  |  |  |
| 2 | Vitvi14g01911\_t001 |  | \| \| |  | \| \| |  |  |  |  |  |  |  |  |
| 2 | Vitvi14g04671\_t001 |  | \| \| |  | \| \| |  |  |  |  |  |  |  |  |
| 2 | Vitvi14g04672\_t001 |  | \| \| |  | \| \| |  |  |  |  |  |  |  |  |
| 2 | Vitvi14g04673\_t001 |  | \| \| |  | \| \| |  |  |  |  |  |  |  |  |
| 2 | Vitvi14g04674\_t001 |  | \| \| |  | \| \| |  |  |  |  |  |  |  |  |
| 2 | Vitvi14g04675\_t001 |  | \| \| |  | \| \| |  |  |  |  |  |  |  |  |
| 2 | Vitvi14g01915\_t001 |  | \| \| |  | \| \| |  |  |  |  |  |  |  |  |
| 2 | Vitvi14g01916\_t001 |  | Vitvi17g00565\_t001 |  | \| \| |  |  |  |  |  |  |  |  |
| 2 | Vitvi14g04676\_t001 |  | \| \| |  | \| \| |  |  |  |  |  |  |  |  |
| 2 | Vitvi14g03069\_t001 |  | \| \| |  | \| \| |  |  |  |  |  |  |  |  |
| 2 | Vitvi14g04677\_t001 |  | \| \| |  | \| \| |  |  |  |  |  |  |  |  |
| 2 | Vitvi14g01917\_t001 |  | \| \| |  | \| \| |  |  |  |  |  |  |  |  |
| 2 | Vitvi14g03072\_t001 |  | \| \| |  | \| \| |  |  |  |  |  |  |  |  |
| 2 | Vitvi14g03074\_t001 |  | \| \| |  | \| \| |  |  |  |  |  |  |  |  |
| 2 | Vitvi14g01919\_t001 |  | \| \| |  | \| \| |  |  |  |  |  |  |  |  |
| 2 | Vitvi14g03075\_t001 |  | \| \| |  | \| \| |  |  |  |  |  |  |  |  |
| 2 | Vitvi14g01920\_t001 |  | \| \| |  | \| \| |  |  |  |  |  |  |  |  |
| 2 | Vitvi14g01921\_t001 |  | \| \| |  | \| \| |  |  |  |  |  |  |  |  |
| 2 | Vitvi14g01922\_t001 |  | \| \| |  | Vitvi01g00958\_t002 |  |  |  |  |  |  |  |  |
| 2 | Vitvi14g01923\_t001 |  | \| \| |  | \| \| |  |  |  |  |  |  |  |  |
| 2 | Vitvi14g03076\_t001 |  | \| \| |  | Vitvi01g00960\_t003 |  |  |  |  |  |  |  |  |
| 2 | Vitvi14g01926\_t001 |  | Vitvi17g00569\_t001 |  | Vitvi01g00964\_t001 |  |  |  |  |  |  |  |  |
| 2 | Vitvi14g03077\_t001 |  | \| \| |  | \| \| |  |  |  |  |  |  |  |  |
| 2 | Vitvi14g04678\_t001 |  | \| \| |  | \| \| |  |  |  |  |  |  |  |  |
| 2 | Vitvi14g04679\_t001 |  | \| \| |  | \| \| |  |  |  |  |  |  |  |  |
| 2 | Vitvi14g01928\_t001 |  | \| \| |  | \| \| |  |  |  |  |  |  |  |  |
| 2 | Vitvi14g01929\_t001 |  | \| \| |  | \| \| |  |  |  |  |  |  |  |  |
| 2 | Vitvi14g01930\_t001 |  | \| \| |  | \| \| |  |  |  |  |  |  |  |  |
| 2 | Vitvi14g03078\_t001 |  | \| \| |  | \| \| |  |  |  |  |  |  |  |  |
| 2 | Vitvi14g04680\_t001 |  | Vitvi17g04155\_t001 |  | Vitvi01g00971\_t001 |  |  |  |  |  |  |  |  |
| 2 | Vitvi14g01932\_t001 |  | \| \| |  | \| \| |  |  |  |  |  |  |  |  |
| 2 | Vitvi14g03079\_t001 |  | Vitvi17g00578\_t001 |  | \| \| |  |  |  |  |  |  |  |  |
| 2 | Vitvi14g01934\_t001 |  | \| \| |  | Vitvi01g00985\_t001 |  |  |  |  |  |  |  |  |
| 2 | Vitvi14g01935\_t001 |  | \| \| |  | \| \| |  |  |  |  |  |  |  |  |
| 2 | Vitvi14g01936\_t001 |  | Vitvi17g00582\_t001 |  | \| \| |  |  |  |  |  |  |  |  |
| 2 | Vitvi14g01937\_t001 |  | \| \| |  | \| \| |  |  |  |  |  |  |  |  |
| 2 | Vitvi14g03080\_t001 |  | \| \| |  | \| \| |  |  |  |  |  |  |  |  |
| 2 | Vitvi14g01938\_t001 |  | \| \| |  | \| \| |  |  |  |  |  |  |  |  |
| 2 | Vitvi14g01939\_t001 |  | \| \| |  | \| \| |  |  |  |  |  |  |  |  |
| 2 | Vitvi14g01940\_t001 |  | \| \| |  | \| \| |  |  |  |  |  |  |  |  |
| 2 | Vitvi14g01941\_t002 |  | \| \| |  | \| \| |  |  |  |  |  |  |  |  |
| 2 | Vitvi14g01942\_t001 |  | \| \| |  | \| \| |  |  |  |  |  |  |  |  |
| 3 | Vitvi14g01943\_t001 |  | \| \| |  | \| \| |  | Vitvi01g01020\_t001 |  |  |  |  |  |  |
| 3 | Vitvi14g01944\_t001 |  | \| \| |  | \| \| |  | \| \| |  |  |  |  |  |  |
| 3 | Vitvi14g01945\_t001 |  | \| \| |  | \| \| |  | Vitvi01g01019\_t001 |  |  |  |  |  |  |
| 3 | Vitvi14g01946\_t001 |  | \| \| |  | \| \| |  | \| \| |  |  |  |  |  |  |
| 3 | Vitvi14g01947\_t001 |  | \| \| |  | \| \| |  | \| \| |  |  |  |  |  |  |
| 3 | Vitvi14g01949\_t001 |  | \| \| |  | \| \| |  | \| \| |  |  |  |  |  |  |
| 3 | Vitvi14g03082\_t001 |  | \| \| |  | \| \| |  | \| \| |  |  |  |  |  |  |
| 3 | Vitvi14g01950\_t001 |  | \| \| |  | \| \| |  | \| \| |  |  |  |  |  |  |
| 3 | Vitvi14g01951\_t001 |  | \| \| |  | \| \| |  | \| \| |  |  |  |  |  |  |
| 3 | Vitvi14g01952\_t001 |  | \| \| |  | \| \| |  | \| \| |  |  |  |  |  |  |
| 3 | Vitvi14g01953\_t001 |  | \| \| |  | \| \| |  | \| \| |  |  |  |  |  |  |
| 3 | Vitvi14g04681\_t001 |  | \| \| |  | \| \| |  | \| \| |  |  |  |  |  |  |
| 3 | Vitvi14g04682\_t001 |  | \| \| |  | \| \| |  | \| \| |  |  |  |  |  |  |
| 3 | Vitvi14g03084\_t001 |  | Vitvi17g00601\_t001 |  | \| \| |  | \| \| |  |  |  |  |  |  |
| 3 | Vitvi14g01955\_t001 |  | \| \| |  | Vitvi01g01013\_t001 |  | \| \| |  |  |  |  |  |  |
| 3 | Vitvi14g01956\_t001 |  | \| \| |  | \| \| |  | Vitvi01g01012\_t001 |  |  |  |  |  |  |
| 3 | Vitvi14g01959\_t002 |  | \| \| |  | \| \| |  | \| \| |  |  |  |  |  |  |
| 3 | Vitvi14g01960\_t002 |  | \| \| |  | \| \| |  | \| \| |  |  |  |  |  |  |
| 3 | Vitvi14g01961\_t002 |  | \| \| |  | \| \| |  | Vitvi01g01009\_t001 |  |  |  |  |  |  |
| 3 | Vitvi14g04683\_t001 |  | \| \| |  | \| \| |  | Vitvi01g04246\_t001 |  |  |  |  |  |  |
| 3 | Vitvi14g01963\_t001 |  | \| \| |  | \| \| |  | Vitvi01g00999\_t001 |  |  |  |  |  |  |
| 2 | Vitvi14g01964\_t001 |  | Vitvi17g00607\_t001 |  | \| \| |  |  |  |  |  |  |  |  |
| 2 | Vitvi14g04684\_t001 |  | \| \| |  | \| \| |  |  |  |  |  |  |  |  |
| 2 | Vitvi14g01965\_t001 |  | \| \| |  | Vitvi01g01022\_t001 |  |  |  |  |  |  |  |  |
| 2 | Vitvi14g04685\_t001 |  | \| \| |  | \| \| |  |  |  |  |  |  |  |  |
| 2 | Vitvi14g04686\_t001 |  | \| \| |  | \| \| |  |  |  |  |  |  |  |  |
| 2 | Vitvi14g03087\_t001 |  | \| \| |  | \| \| |  |  |  |  |  |  |  |  |
| 2 | Vitvi14g04687\_t001 |  | \| \| |  | \| \| |  |  |  |  |  |  |  |  |
| 2 | Vitvi14g01966\_t001 |  | \| \| |  | \| \| |  |  |  |  |  |  |  |  |
| 2 | Vitvi14g01967\_t001 |  | \| \| |  | \| \| |  |  |  |  |  |  |  |  |
| 2 | Vitvi14g01968\_t001 |  | \| \| |  | \| \| |  |  |  |  |  |  |  |  |
| 2 | Vitvi14g01970\_t001 |  | \| \| |  | \| \| |  |  |  |  |  |  |  |  |
| 2 | Vitvi14g01971\_t001 |  | \| \| |  | Vitvi01g01023\_t001 |  |  |  |  |  |  |  |  |
| 2 | Vitvi14g03088\_t001 |  | \| \| |  | \| \| |  |  |  |  |  |  |  |  |
| 2 | Vitvi14g01972\_t001 |  | \| \| |  | \| \| |  |  |  |  |  |  |  |  |
| 2 | Vitvi14g04688\_t001 |  | \| \| |  | \| \| |  |  |  |  |  |  |  |  |
| 2 | Vitvi14g01973\_t001 |  | Vitvi17g00611\_t001 |  | Vitvi01g01026\_t001 |  |  |  |  |  |  |  |  |
| 2 | Vitvi14g03089\_t001 |  | \| \| |  | \| \| |  |  |  |  |  |  |  |  |
| 2 | Vitvi14g01975\_t001 |  | Vitvi17g01471\_t001 |  | Vitvi01g01027\_t001 |  |  |  |  |  |  |  |  |
| 2 | Vitvi14g01976\_t001 |  | \| \| |  | Vitvi01g01028\_t001 |  |  |  |  |  |  |  |  |
| 2 | Vitvi14g01977\_t001 |  | Vitvi17g00616\_t001 |  | Vitvi01g01030\_t001 |  |  |  |  |  |  |  |  |
| 2 | Vitvi14g01978\_t001 |  | \| \| |  | \| \| |  |  |  |  |  |  |  |  |
| 2 | Vitvi14g01981\_t002 |  | \| \| |  | Vitvi01g01034\_t001 |  |  |  |  |  |  |  |  |
| 2 | Vitvi14g01983\_t001 |  | \| \| |  | \| \| |  |  |  |  |  |  |  |  |
| 2 | Vitvi14g03091\_t001 |  | \| \| |  | \| \| |  |  |  |  |  |  |  |  |
| 2 | Vitvi14g01984\_t001 |  | Vitvi17g01474\_t001 |  | \| \| |  |  |  |  |  |  |  |  |
| 2 | Vitvi14g01985\_t001 |  | Vitvi17g00622\_t001 |  | \| \| |  |  |  |  |  |  |  |  |
| 2 | Vitvi14g03092\_t001 |  | \| \| |  | \| \| |  |  |  |  |  |  |  |  |
| 2 | Vitvi14g04689\_t001 |  | \| \| |  | \| \| |  |  |  |  |  |  |  |  |
| 2 | Vitvi14g01986\_t001 |  | \| \| |  | \| \| |  |  |  |  |  |  |  |  |
| 2 | Vitvi14g01987\_t001 |  | Vitvi17g00623\_t001 |  | Vitvi01g01052\_t001 |  |  |  |  |  |  |  |  |
| 2 | Vitvi14g04690\_t001 |  | \| \| |  | \| \| |  |  |  |  |  |  |  |  |
| 2 | Vitvi14g01989\_t001 |  | \| \| |  | \| \| |  |  |  |  |  |  |  |  |
| 2 | Vitvi14g04691\_t001 |  | \| \| |  | \| \| |  |  |  |  |  |  |  |  |
| 2 | Vitvi14g01990\_t001 |  | \| \| |  | \| \| |  |  |  |  |  |  |  |  |
| 2 | Vitvi14g01991\_t001 |  | \| \| |  | \| \| |  |  |  |  |  |  |  |  |
| 2 | Vitvi14g01992\_t001 |  | \| \| |  | Vitvi01g01077\_t001 |  |  |  |  |  |  |  |  |
| 2 | Vitvi14g03093\_t001 |  | \| \| |  | Vitvi01g01056\_t001 |  |  |  |  |  |  |  |  |
| 1 | Vitvi14g01993\_t001 |  | Vitvi17g00634\_t001 |  |  |  |  |  |  |  |  |  |  |
| 1 | Vitvi14g01994\_t001 |  | \| \| |  |  |  |  |  |  |  |  |  |  |
| 1 | Vitvi14g01995\_t001 |  | \| \| |  |  |  |  |  |  |  |  |  |  |
| 1 | Vitvi14g01996\_t001 |  | \| \| |  |  |  |  |  |  |  |  |  |  |
| 1 | Vitvi14g01997\_t001 |  | \| \| |  |  |  |  |  |  |  |  |  |  |
| 1 | Vitvi14g03095\_t001 |  | \| \| |  |  |  |  |  |  |  |  |  |  |
| 1 | Vitvi14g01999\_t001 |  | Vitvi17g00641\_t001 |  |  |  |  |  |  |  |  |  |  |
| 1 | Vitvi14g02000\_t001 |  | \| \| |  |  |  |  |  |  |  |  |  |  |
| 1 | Vitvi14g02001\_t001 |  | \| \| |  |  |  |  |  |  |  |  |  |  |
| 1 | Vitvi14g02002\_t003 |  | \| \| |  |  |  |  |  |  |  |  |  |  |
| 1 | Vitvi14g04692\_t001 |  | \| \| |  |  |  |  |  |  |  |  |  |  |
| 1 | Vitvi14g02004\_t001 |  | Vitvi17g00645\_t001 |  |  |  |  |  |  |  |  |  |  |
| 1 | Vitvi14g02005\_t001 |  | \| \| |  |  |  |  |  |  |  |  |  |  |
| 1 | Vitvi14g02006\_t001 |  | Vitvi17g00648\_t001 |  |  |  |  |  |  |  |  |  |  |
| 1 | Vitvi14g02007\_t001 |  | \| \| |  |  |  |  |  |  |  |  |  |  |
| 1 | Vitvi14g02008\_t001 |  | \| \| |  |  |  |  |  |  |  |  |  |  |
| 1 | Vitvi14g02009\_t001 |  | \| \| |  |  |  |  |  |  |  |  |  |  |
| 1 | Vitvi14g03096\_t001 |  | \| \| |  |  |  |  |  |  |  |  |  |  |
| 1 | Vitvi14g02010\_t001 |  | \| \| |  |  |  |  |  |  |  |  |  |  |
| 1 | Vitvi14g02011\_t001 |  | \| \| |  |  |  |  |  |  |  |  |  |  |
| 1 | Vitvi14g02012\_t001 |  | \| \| |  |  |  |  |  |  |  |  |  |  |
| 1 | Vitvi14g02013\_t001 |  | \| \| |  |  |  |  |  |  |  |  |  |  |
| 1 | Vitvi14g02014\_t001 |  | Vitvi17g00660\_t001 |  |  |  |  |  |  |  |  |  |  |
| 1 | Vitvi14g02015\_t001 |  | \| \| |  |  |  |  |  |  |  |  |  |  |
| 1 | Vitvi14g03097\_t001 |  | \| \| |  |  |  |  |  |  |  |  |  |  |
| 1 | Vitvi14g02016\_t001 |  | \| \| |  |  |  |  |  |  |  |  |  |  |
| 1 | Vitvi14g04693\_t001 |  | \| \| |  |  |  |  |  |  |  |  |  |  |
| 1 | Vitvi14g04694\_t001 |  | \| \| |  |  |  |  |  |  |  |  |  |  |
| 1 | Vitvi14g02018\_t001 |  | \| \| |  |  |  |  |  |  |  |  |  |  |
| 1 | Vitvi14g04695\_t001 |  | \| \| |  |  |  |  |  |  |  |  |  |  |
| 1 | Vitvi14g02020\_t001 |  | Vitvi17g00665\_t001 |  |  |  |  |  |  |  |  |  |  |
| 1 | Vitvi14g02021\_t001 |  | \| \| |  |  |  |  |  |  |  |  |  |  |
| 1 | Vitvi14g02022\_t001 |  | Vitvi17g00666\_t001 |  |  |  |  |  |  |  |  |  |  |
| 1 | Vitvi14g02023\_t003 |  | \| \| |  |  |  |  |  |  |  |  |  |  |
| 1 | Vitvi14g02024\_t002 |  | \| \| |  |  |  |  |  |  |  |  |  |  |
| 1 | Vitvi14g02025\_t001.1.6037826b |  | \| \| |  |  |  |  |  |  |  |  |  |  |
| 1 | Vitvi14g02026\_t001 |  | \| \| |  |  |  |  |  |  |  |  |  |  |
| 1 | Vitvi14g02027\_t001 |  | \| \| |  |  |  |  |  |  |  |  |  |  |
| 1 | Vitvi14g02028\_t001 |  | \| \| |  |  |  |  |  |  |  |  |  |  |
| 1 | Vitvi14g02029\_t001 |  | \| \| |  |  |  |  |  |  |  |  |  |  |
| 1 | Vitvi14g02030\_t001 |  | \| \| |  |  |  |  |  |  |  |  |  |  |
| 1 | Vitvi14g02031\_t001 |  | \| \| |  |  |  |  |  |  |  |  |  |  |
| 1 | Vitvi14g02032\_t001 |  | \| \| |  |  |  |  |  |  |  |  |  |  |
| 1 | Vitvi14g04696\_t001 |  | Vitvi17g01488\_t001 |  |  |  |  |  |  |  |  |  |  |
| 1 | Vitvi14g02033\_t001 |  | \| \| |  |  |  |  |  |  |  |  |  |  |
| 1 | Vitvi14g02034\_t001 |  | \| \| |  |  |  |  |  |  |  |  |  |  |
| 1 | Vitvi14g03099\_t002 |  | Vitvi17g00682\_t002 |  |  |  |  |  |  |  |  |  |  |
| 0 | Vitvi14g02039\_t001 |  |  |  |  |  |  |  |  |  |  |  |  |
| 0 | Vitvi14g04697\_t001 |  |  |  |  |  |  |  |  |  |  |  |  |
| 0 | Vitvi14g04698\_t001 |  |  |  |  |  |  |  |  |  |  |  |  |
| 0 | Vitvi14g02042\_t001 |  |  |  |  |  |  |  |  |  |  |  |  |
| 0 | Vitvi14g02043\_t001 |  |  |  |  |  |  |  |  |  |  |  |  |
| 0 | Vitvi14g04699\_t001 |  |  |  |  |  |  |  |  |  |  |  |  |
| 0 | Vitvi14g02046\_t001 |  |  |  |  |  |  |  |  |  |  |  |  |
| 0 | Vitvi14g04700\_t001 |  |  |  |  |  |  |  |  |  |  |  |  |
| 0 | Vitvi14g04701\_t001 |  |  |  |  |  |  |  |  |  |  |  |  |
| 0 | Vitvi14g04702\_t001 |  |  |  |  |  |  |  |  |  |  |  |  |
